# Supplementary material for: Carbon neutral hydrogen storage and release cycles based on dual-functional roles of formamides
Source: Nat Commun. 2023 Jun 22;14:3726. doi: 10.1038/s41467-023-39309-4 (PMC10287642; doi:10.1038/s41467-023-39309-4)
Supplement: Supplementary file 1 — Supplementary Information [file 41467_2023_39309_MOESM1_ESM.pdf]

Supplementary Information

Carbon neutral hydrogen storage and release cycles based on dual-functional roles of formamides

Duo Wei,[1,2] Xinzhe Shi,[1,2] Henrik Junge,[2*] Chunyu Du[1*] and Matthias Beller[2*]

[1] School of Chemistry and Chemical Engineering, Harbin Institute of Technology, Harbin 150001, P. R. China.

[2] Leibniz-Institut für Katalyse e.V., 18059 Rostock, Germany.

*Corresponding authors. *Email: henrik.junge@catalysis.de; cydu@hit.edu.cn; matthias.beller@catalysis.de*

Supplementary methods

Materials and characterization methods

Unless otherwise stated, all reactions were conducted under an argon atmosphere and all reagents were purchased from commercial suppliers and used without further purification, including *N*-formylmorpholine (F_1, TCI, >99%), *N*-formylpiperidine (F_2, TCI, >98%), 1-formylpiperazine (F_3, TCI, >98 %), 1,4-diformylpiperazine (F_4, Alfa Aesar, >98%), methanamide (MA, Sigma-Aldrich, >99.5%), potassium hydroxide (Fisher Chemical, 86.4%), potassium bicarbonate (Sigma-Aldrich, 99.5%), potassium formate (Sigma-Aldrich, 99%), formic acid (FA, Sigma-Aldrich, 98%), morpholine (A_1, TCI, >99%), piperidine (A_2, Alfa Aesar, 99%), piperazine (A_3, Alfa Aesar, 99%), L-lysine (Lys, fluorochem, 97%), L-arginine (Arg, Alfa Aesar, >98%), 1,4-diazabicyclo[2.2.2]octane (DABCO, TCI, >98%), 1,8-diazabicyclo[5.4.0]undec-7-ene (DBU, TCI, >98%), 1,1,3,3-tetramethylguanidine (TMG, TCI, >99%), trihexylamine (THA, TCI, >98%), *N*,*N*-dimethyloctylamine (DMOA, Alfa Aesar, 99%), 2-methyltetrahydrofuran (2-MTHF, Alfa Aesar, 99%), ethanol (Acros Organics, 99.5%), triglyme (Alfa Aesar, 99%), dioxane (Acros Organics, >99%), dimethyl sulfoxide (DMSO, Acros Organics, >99.7%), imidazole (fluorochem, 99%) deuterium oxide (Deutero, D-99.9%), tetrahydrofuran-d^8 (Deutero, D-99.5%), H_2 (Air Liquide, grade 5.0), CO_2 (Linde, grade 4.8). Organic solvents *N*,*N*-dimethylformamide (DMF), tetrahydrofuran (THF, without stabilizer), were collected from an SPS machine and stored under argon with molecular sieve. THF and deionized water were used for hydrogenation and dehydrogenation reactions.

Organometallic compounds **Fe-1**,[1] **Fe-2**,[2] **Fe-3**,[1] **Fe-4**,[3,4] **Mn-1**,[5,6] **Mn-2**,[7] **Mn-3**,[7] **Co-1**,[8] **Mo-1**,[9] were synthesized according to the corresponding publications and stored under argon with light exclusion. **Fe-4** was prepared by adding 1.1 equiv. of $HN(CH_2CH_2P(CH(CH_3)_2)_2)_2$ in THF to anhydrous $FeBr_2$ (107.8 mg, 0.5 mmol) in THF (10 mL) and the argon inside the flask was replaced with CO by performing freeze-pump-thaw cycles. The solution was stirred at r.t. (25 °C) for 4 h followed by removal of solvent under vacuum. The residue was washed with pentane 3 times and dried under vacuum to give **Fe-4** as a blue powder. **Fe-3** and **Fe-1** were prepared by adding 1 and 5 equiv. of $NaBH_4$ (18.9 mg, 0.5 mmol and 94.6 mg, 2.5 mmol) respectively into **Fe-4** (274.5 mg, 0.5 mmol) in EtOH (35 mL). The reaction mixture was then stirred for 5 h at r.t. The solvent was removed in vacuo and the residue was dissolved in toluene. The resulting suspension was filtered through a short pad of celite, then concentrated in vacuo and the resulting solid was washed thoroughly with pentane to give yellow powder. **Fe-2** was prepared by adding 1.2 equiv. of *t*BuOK (67.3 mg, 0.6 mmol) into the solution of **Fe-3** (235.1 mg, 0.5 mmol) in THF (20 mL). The suspension was stirred for 0.5 h at r.t. Formic acid (18.9 µL, 0.5 mmol) was added to the solution at r.t. The yellow solution was stirred for 0.5 h at r.t. Then the solvent was evaporated under vacuum. The yellow residue was recrystallized from pentane at -30 °C to give **Fe-2** as a yellow powder. **Mn-1**, **Mn-2**, **Mn-3**, and **Co-1** were prepared by adding $Mn(CO)_5Br$ (137.4 mg, 0.5 mmol) or $CoBr_2$ (109.4 mg, 0.5 mmol) dropwise in THF (10 mL) respectively to 1.1 equiv. of corresponding pincer ligands in THF then stirred and heated at 100 °C for 20 h under argon. The reaction mixture was cooled to r.t. and concentrated in vacuo. The crude mixture was then thoroughly washed with pentane and dried under vacuum affording corresponding complexes as powder (yellow for **Mn-1**, **Mn-2**, **Mn-3**, and purple for **Co-1**). **Mo-1** was prepared by adding 1.1 equiv. of formic acid (20.7 µL, 0.55 mmol) dropwise into a solution of $\{Mo(CH_3CN)(CO)_2[HN(CH_2CH_2P(CH(CH_3)_2)_2)_2]\}$ (250 mg, 0.5 mmol) in toluene (20 mL). The pale-yellow solution was stirred for 1 h at r.t. then filtered through a short pad of celite and concentrated to about 3 mL. Diethyl ether (15 mL) were added to precipitate the desired compound which was filtered and further washed with diethyl ether, then dried in vacuo to give **Mo-1** as a pale-yellow powder.

^1H and ^{13}C spectroscopy were recorded using Bruker AV 300 MHz and Bruker AV 400 MHz spectrometers. All NMR data in the experimental section are expressed as chemical shift in parts per million (ppm). ^1H chemical shifts were determined relative to the internal standard (I.S.) imidazole (7.14 and 7.78 ppm) in D_2O. ^{13}C chemical shifts were determined relative to the internal standard imidazole (122.43 and 136.65 ppm) in D_2O. NMR spectra were interpreted and processed using MestReNova (version 14.0.1-23559). Gas chromatography (Agilent Technologies 7890A GC system, Carboxen / TCD) was used to analyze the content of the gas phase with a CO quantification limit of 10 ppm.

Calculation of the hydrogen volume, mole, yield, selectivity.

The gas evolution was corrected with the blank volume (18 mL) which corresponds to the gas evolution of the same reaction in the absence of catalyst.

H_2 volume V_{H_2} and CO_2 volume V_{CO_2} are calculated with the following equation:

$$V_{H_2} = (V_{obs} - V_{blank}) \times \frac{\%V_{H_2}}{\%V_{H_2} + \%V_{CO_2}}$$

$$V_{CO_2} = (V_{obs} - V_{blank}) \times \frac{\%V_{CO_2}}{\%V_{H_2} + \%V_{CO_2}}$$

Moles of H_2 n_{H_2} and moles of CO_2 n_{CO_2} are calculated with the following equation:

$$n_{H_2} = \frac{V_{H_2}}{V_{m_{H_2\,(25^oC)}}}$$

$$n_{CO_2} = \frac{V_{CO_2}}{V_{m_{CO_2\,(25^oC)}}}$$

Hydrogen yield Y_{H_2} is calculated with the following equation:

$$Y_{H_2} = \frac{n_{H_2}}{n_{Sub}} \times 100\%$$

Hydrogen selectivity S_{H_2} is calculated with the following equation:

$$S_{H_2} = \frac{n_{H_2}}{n_{H_2} + n_{CO_2}} \times 100\%$$

Where:

- V_{obs} is the gas evolution volume of catalytic reaction measured in the manual burettes.
- V_{blank} is the gas evolution volume of the blank reaction measured in the manual burettes.
- $\%V_{H_2}$ and $\%V_{CO_2}$ are the volume ratios of H_2 and CO_2, respectively, determined by GC.
- n_{Sub} is the mole of substrate.
- $V_{m_{H_2\,(25^oC)}}$ and $V_{m_{CO_2\,(25^oC)}}$ are the molar volumes of H_2 and CO_2 at room temperature (25 °C), respectively, calculated with the Van Der Waals equation, see below:

Calculation of H_2 molar volume $V_{m_{H_2\,(25^oC)}}$:	Calculation of CO_2 molar volume $V_{m_{CO_2\,(25^oC)}}$:
$V_{m_{H_2\,(25^oC)}} = \frac{R \times T}{p} + b - \frac{a}{R \times T} = 24.48$<br>Where:<br>• R = 8.3145 $m^3 \cdot Pa \cdot mol^{-1} \cdot K^{-1}$<br>• T = 273.15 + room temperature (°C) K<br>• P = 101325 Pa<br>• a = 24.9×10^{-3} $Pa \cdot m^6 \cdot mol^{-2}$<br>• b = 26.7×10^{-6} $m^3 \cdot mol^{-1}$	$V_{m_{CO_2\,(25^oC)}} = \frac{R \times T}{p} + b - \frac{a}{R \times T} = 24.36$<br>Where:<br>• R = 8.3145 $m^3 \cdot Pa \cdot mol^{-1} \cdot K^{-1}$<br>• T = 273.15 + room temperature (°C) K<br>• P = 101325 Pa<br>• a = 36.5×10^{-2} $Pa \cdot m^6 \cdot mol^{-2}$<br>• b = 42.7×10^{-6} $m^3 \cdot mol^{-1}$

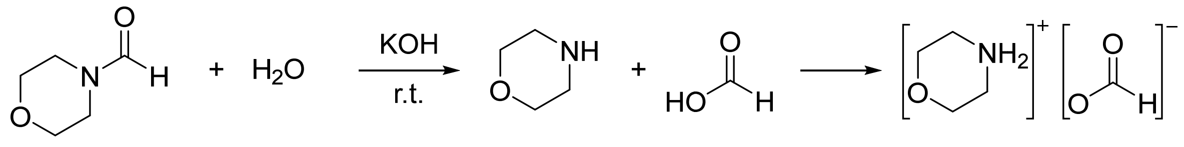

F$_1$ **A$_1$**

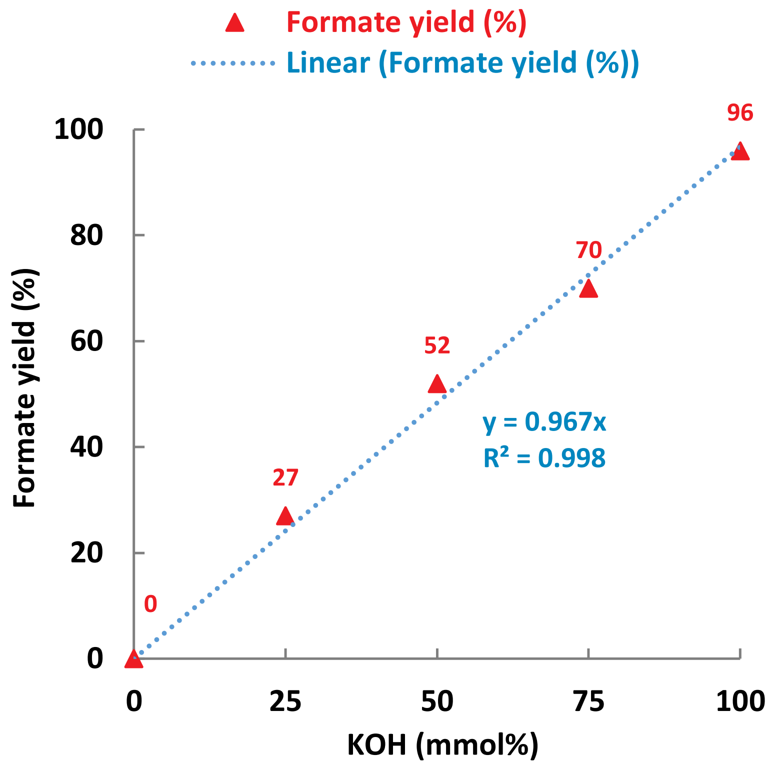

Figure S1. Hydrolysis of *N*-formylmorpholine under alkaline condition. Standard conditions: *N*-formylmorpholine (F_1, 1.0 mmol), KOH (0-100 mol%), H_2O (1 mL), r.t. (25 °C), 0.5 h. The amount of formate was measured by ^{1}H NMR with imidazole (0.25 mmol) as internal standard[10] and the yield of potassium formate is calculated by (mmol potassium formate)/(mmol *N*-formylmorpholine)×100%.

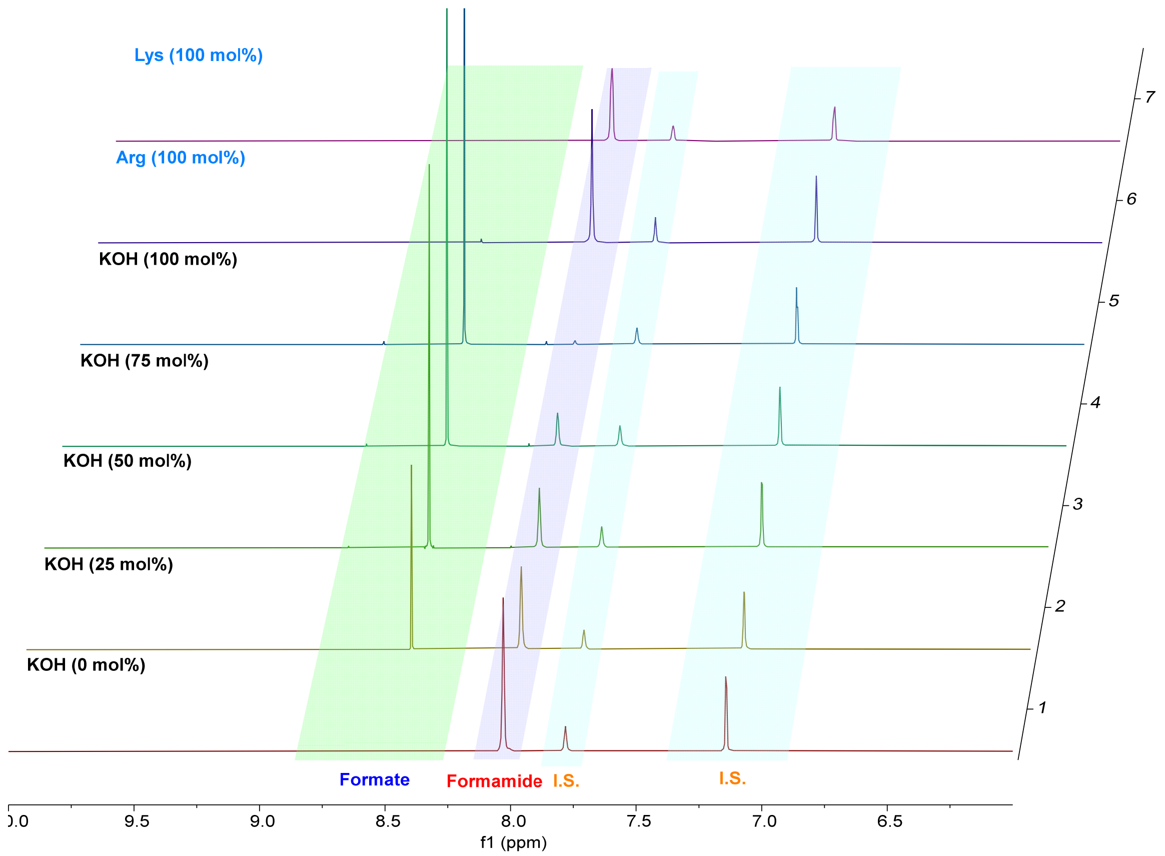

Figure S2. ^{1}H NMR (D_2O) of hydrolysis of *N*-formylmorpholine in the presence of KOH, arginine (Arg), lysine (Lys). Imidazole (0.25 mmol) as internal standard (I.S.).

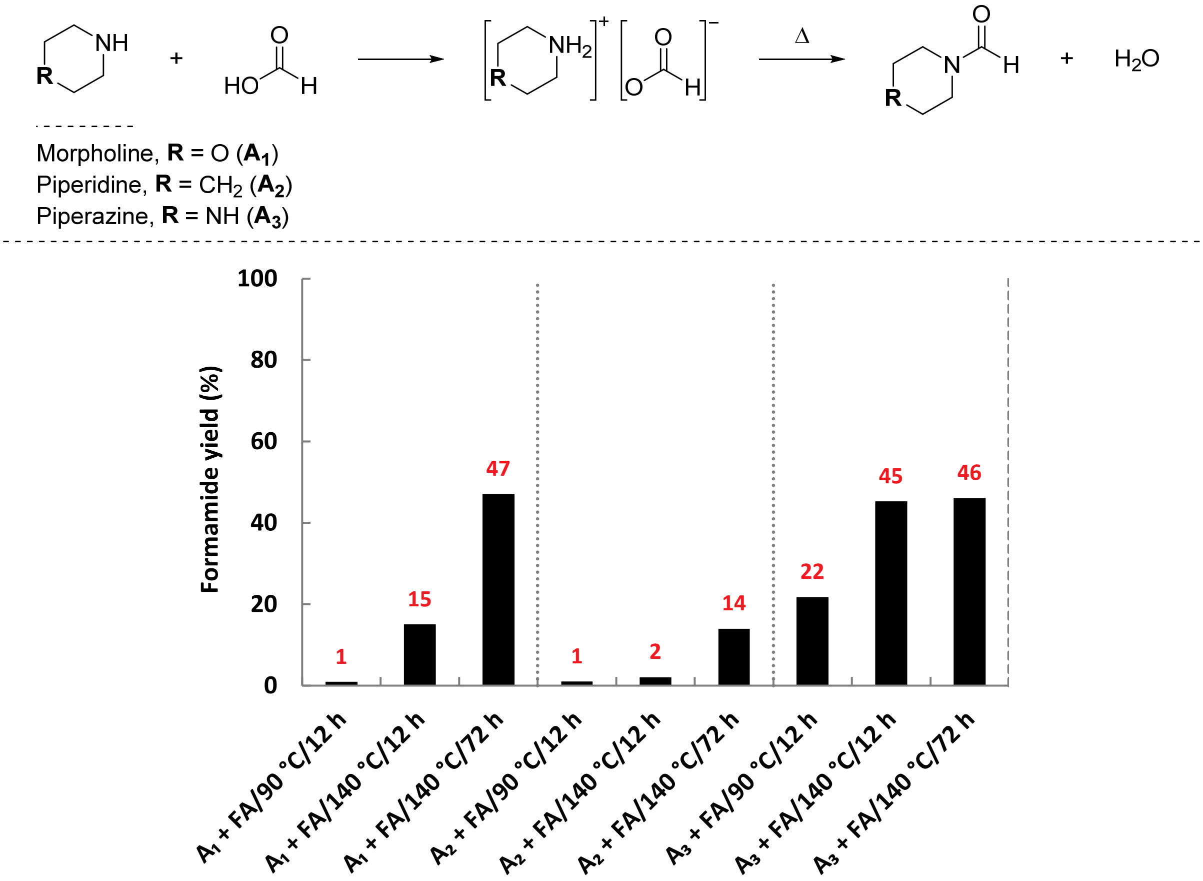

Figure S3. Dehydration condensation between amines and formic acid (FA) to produce formamides. Standard conditions: amine (5 mmol), formic acid (5 mmol), H_2O (5 mL). The amount of formamide were measured by ^{1}H NMR with imidazole (1.25 mmol) as internal standard[10] and the yield of formamide is calculated by (mmol formamide)/(mmol formic acid)×100%. The dotted lines serve as guides to the eye.

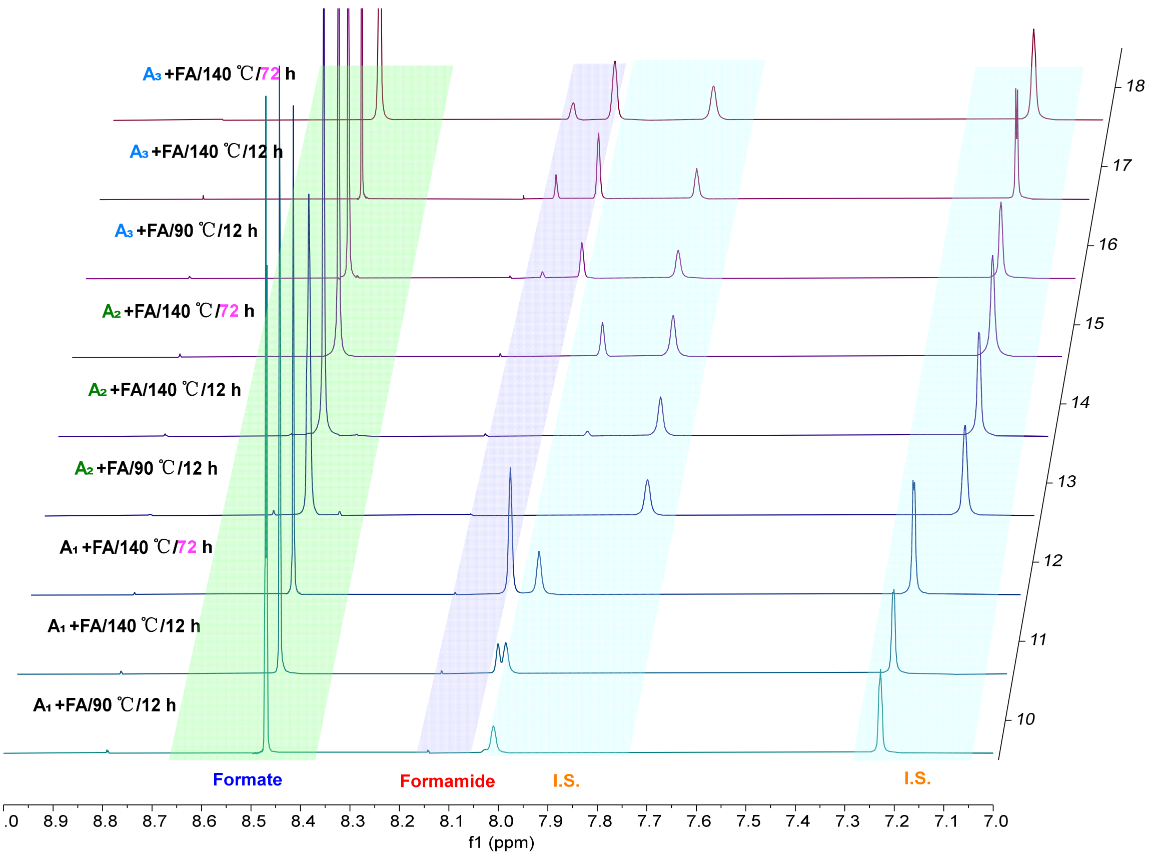

Figure S4. ^{1}H NMR (D_2O) of dehydration condensation between amines and formic acid (FA) to produce formamides. Imidazole (1.25 mmol) as internal standard (I.S.). Chemical shifts were calibrated based on formate (8.43 ppm) in D_2O. The chemical shift of internal standard imidazole is slightly shifted due to presented different pH of the reaction solutions.

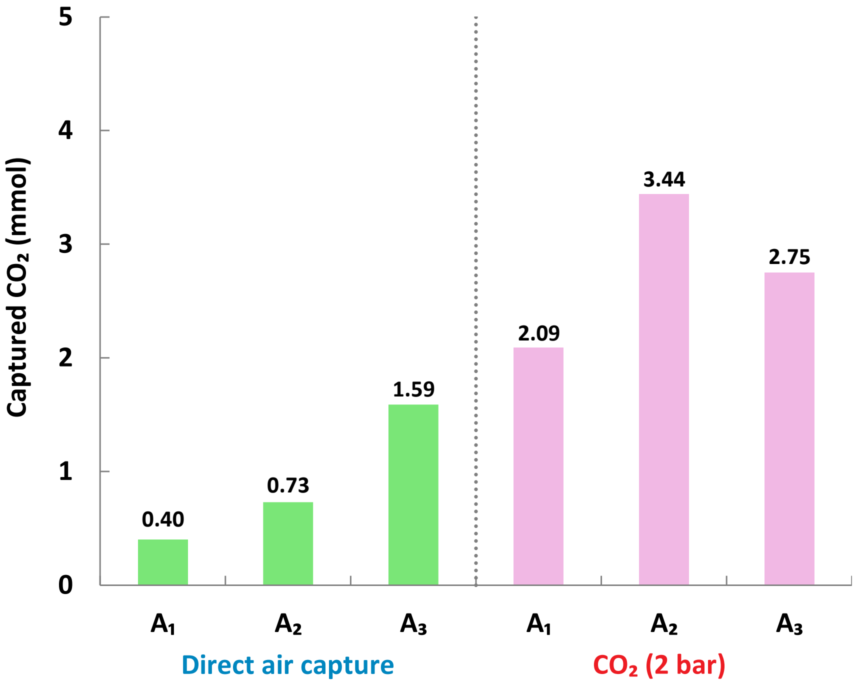

Figure S5. CO_2 capture applying morpholine (A_1), piperidine (A_2) and piperazine (A_3). Standard conditions: morpholine (A_1, 5 mmol), piperidine (A_2, 5 mmol) or piperazine (A_3, 2.5 mmol), CO_2 source (air flow 1.8 L min^{-1}, 36 h or CO_2 2 bar, 30 min), H_2O (1 mL), r.t. (25 °C). The amount of captured CO_2 was measured by ^{13}C-quant NMR with imidazole (1.25 mmol) as internal standard.[10,11] The dotted lines serve as guides to the eye.

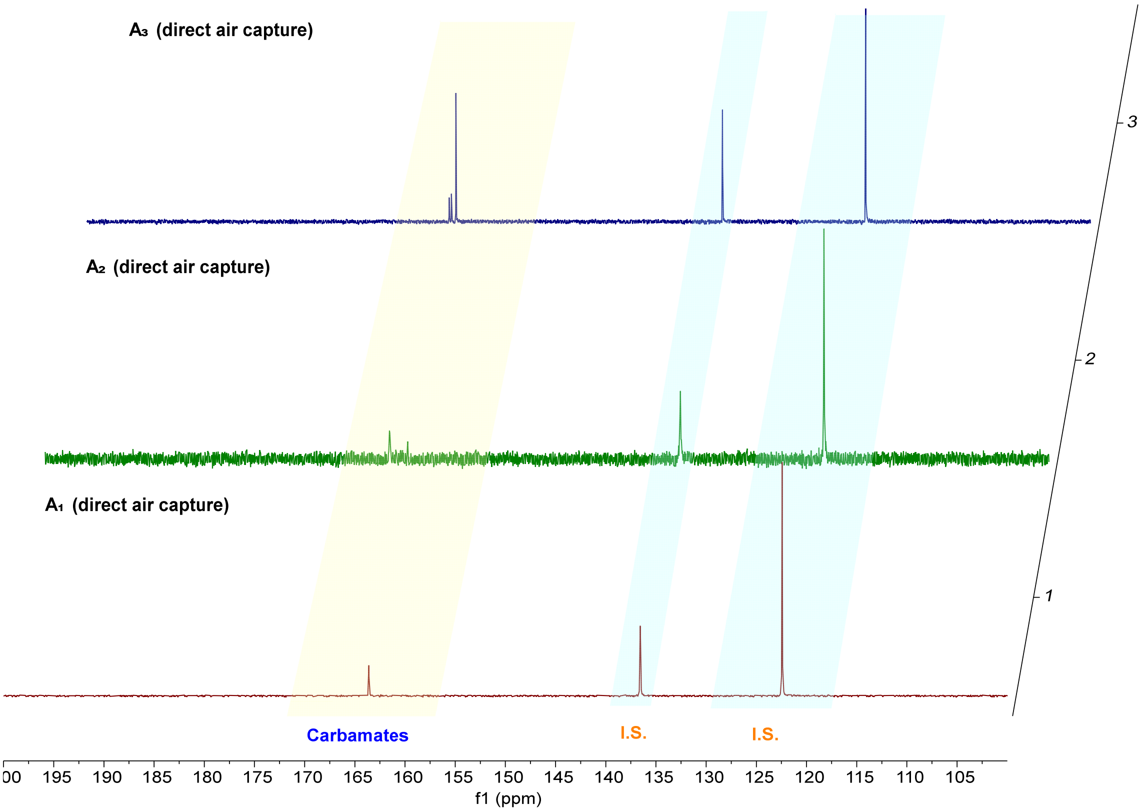

Figure S6. ^{13}C-quant NMR (D_2O) of <u>direct air capture</u> (air flow 1.8 L min^{-1}, 36 h) applying morpholine (A_1, 5 mmol), piperidine (A_2, 5 mmol) and piperazine (A_3, 2.5 mmol). Imidazole (1.25 mmol) as internal standard (I.S.). Carbamate species as products.

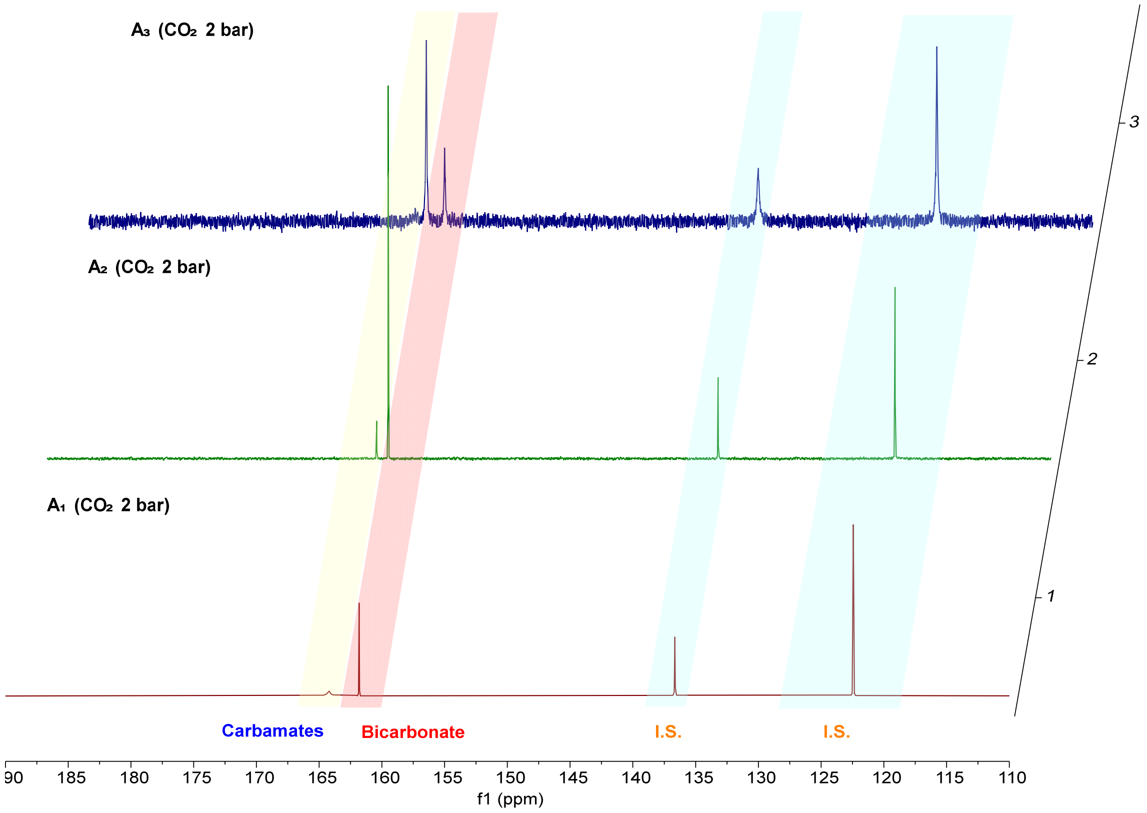

Figure S7. ^{13}C-quant NMR (D_2O) of <u>CO_2 capture</u> (CO_2 2 bar, 30 min) applying morpholine (A_1, 5 mmol), piperidine (A_2, 5 mmol) and piperazine (A_3, 2.5 mmol). Imidazole (1.25 mmol) as internal standard (I.S.). Bicarbonate and carbamate species as products.

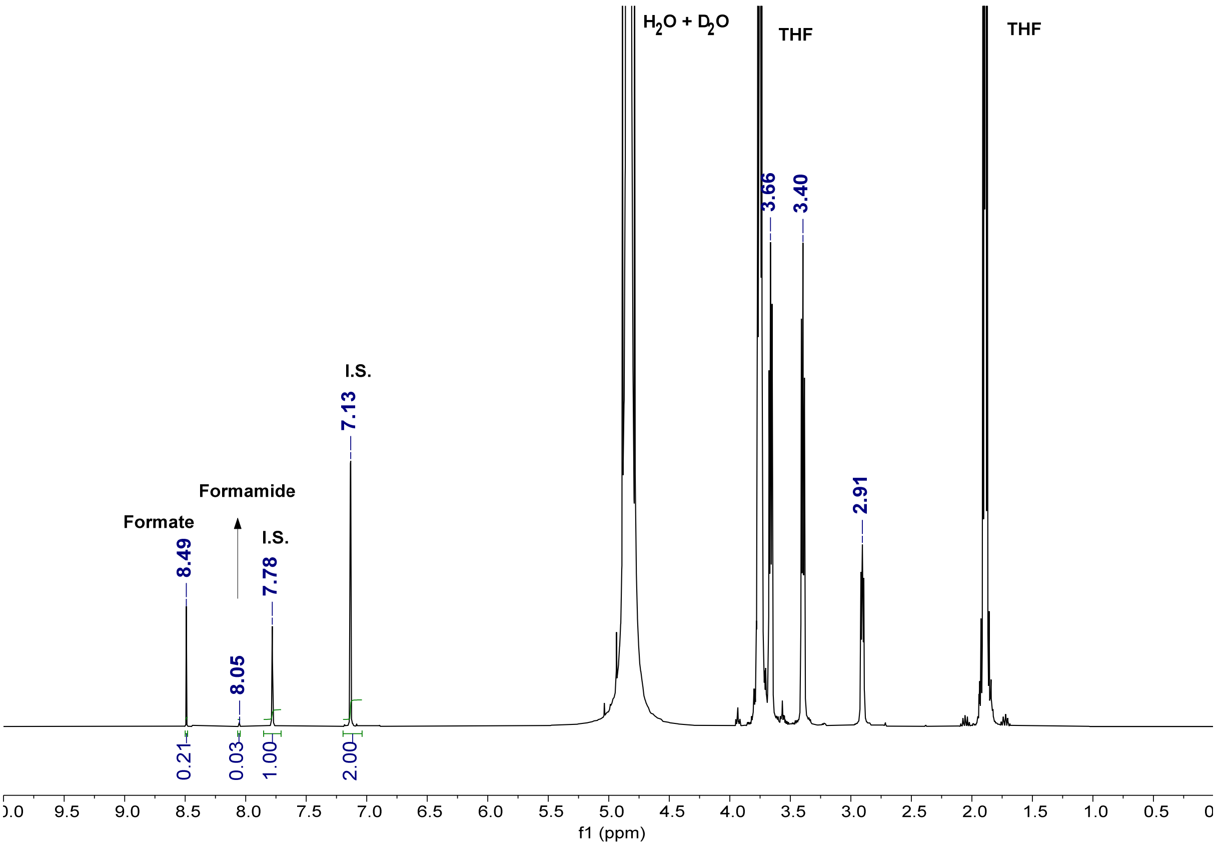

Figure S8. ^{1}H NMR (D_2O) of H_2 production from *N*-formylmorpholine (F_1, 10 mmol), KOH (10 mmol), **Fe-1** (5 µmol). >99% formamide conversion, 99% H_2 yield, >99% H_2 selectivity. Imidazole (2.5 mmol) as internal standard (I.S.).

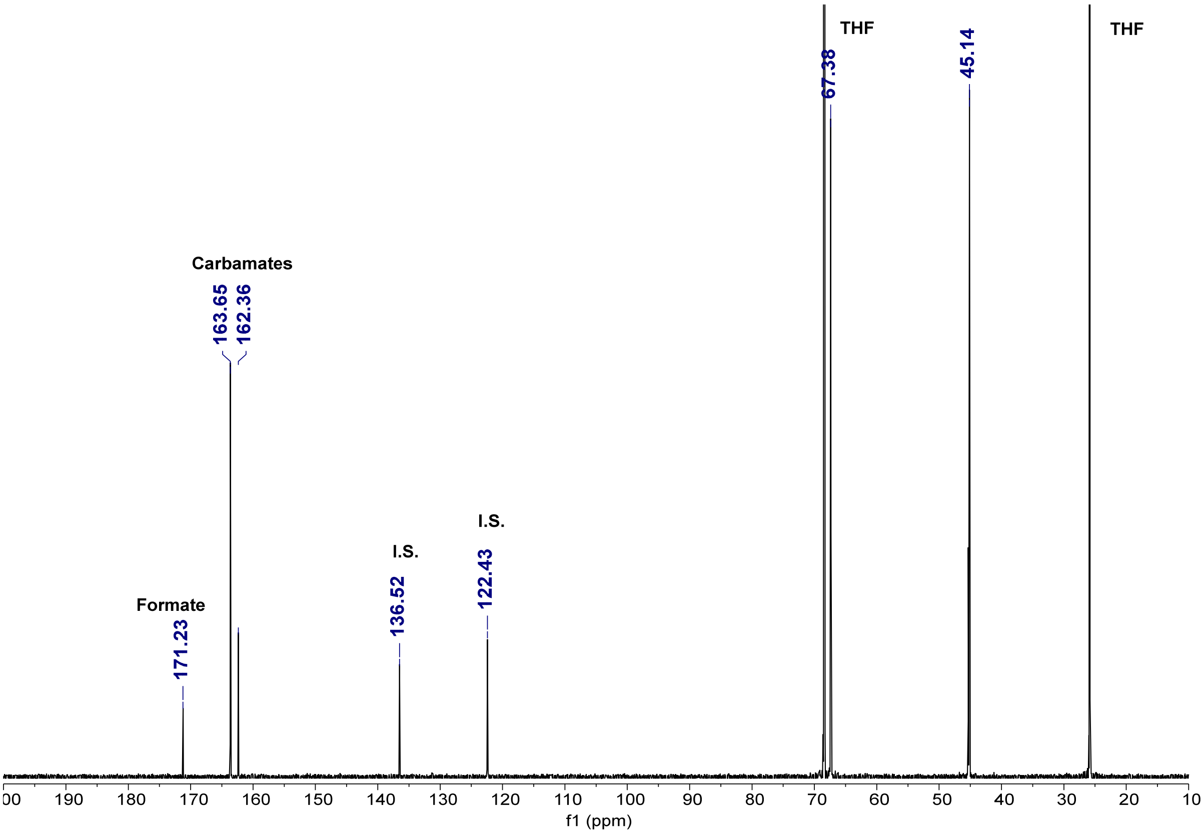

Figure S9. ^{13}C NMR (D_2O) of H_2 production from *N*-formylmorpholine (F_1, 10 mmol), KOH (10 mmol), **Fe-1** (5 μmol). >99% formamide conversion, 99% H_2 yield, >99% H_2 selectivity. Imidazole (2.5 mmol) as internal standard (**I**.S.).

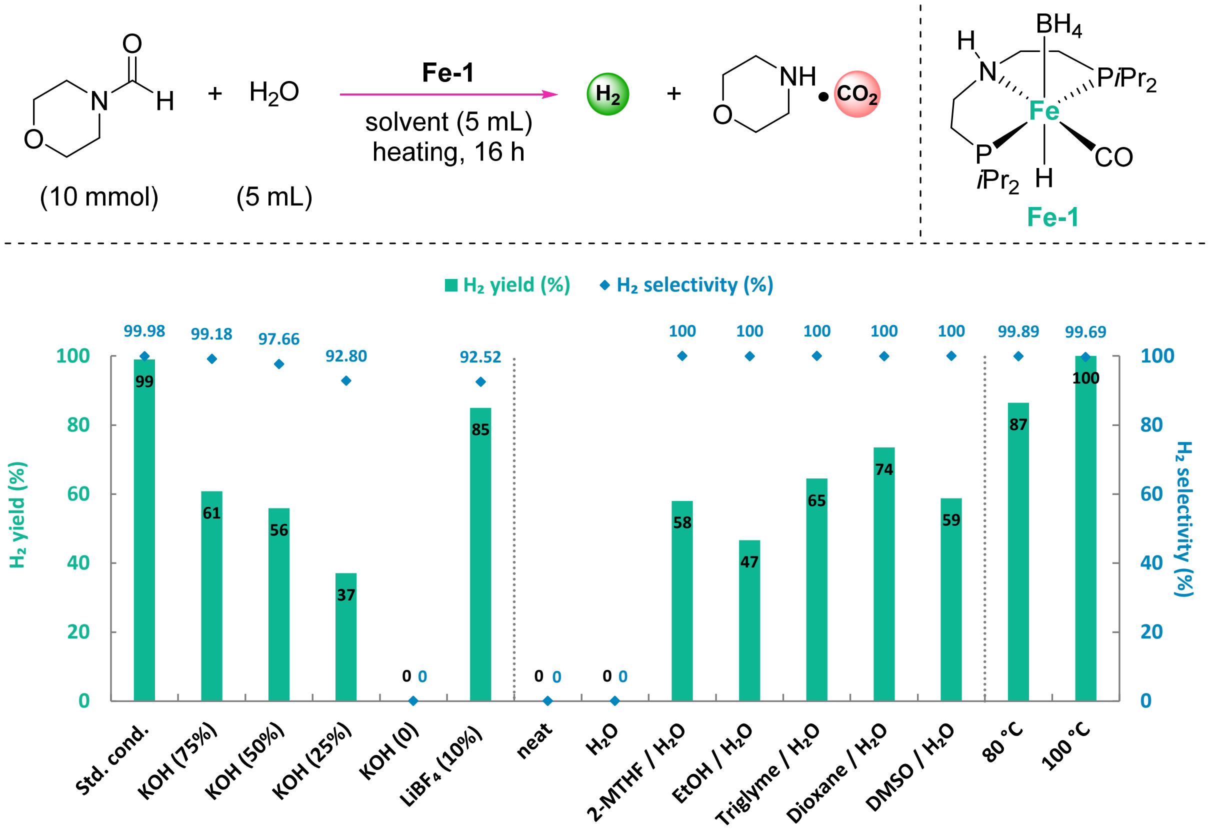

Figure S10. Comparison of the catalytic activity applying different KOH loading (0 to 100 mol%), solvent, and temperature. Standard conditions: *N*-formylmorpholine (F_1, 10 mmol), KOH (0 to 100 mol%), **Fe-1** (5 µmol), organic solvent/H_2O (5/5 mL), 90 °C, 16 h. The dotted lines serve as guides to the eye.

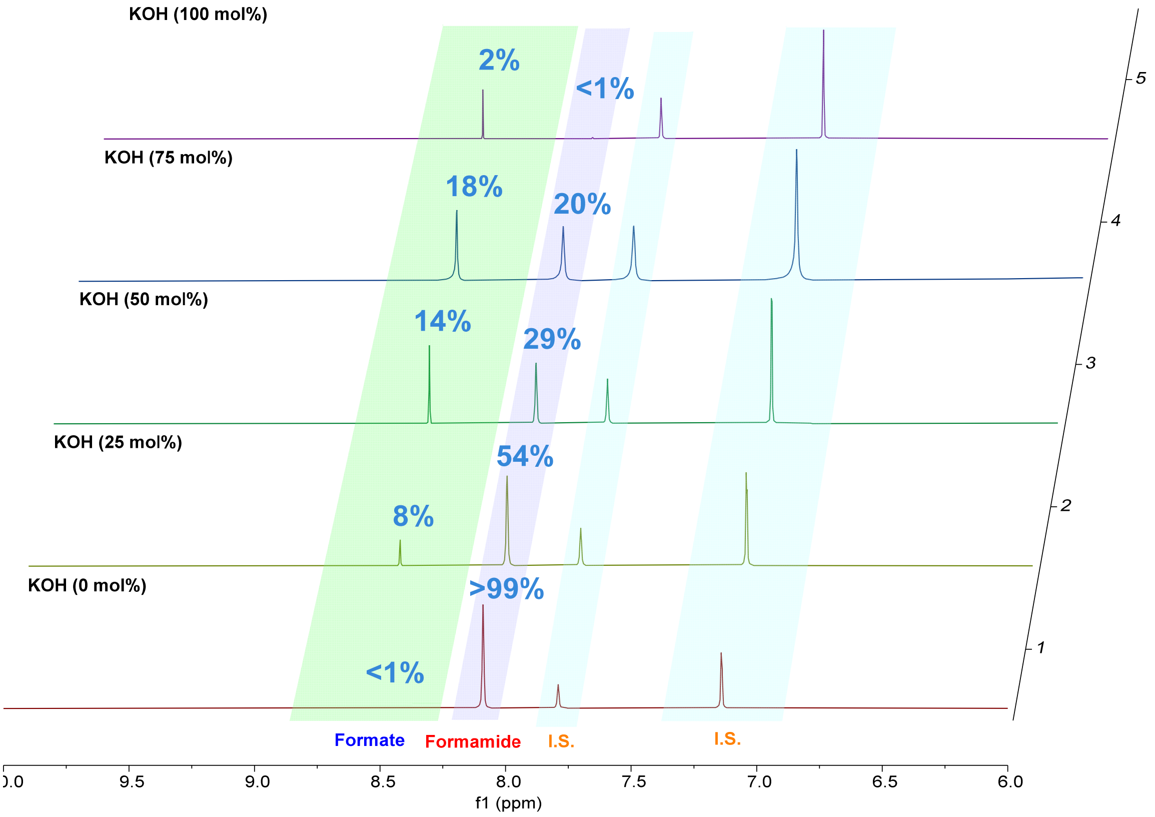

Figure S11. ^{1}H NMR monitoring on the effect of KOH loading (0 to 100 mol%) in the catalytic dehydrogenation starting from *N*-formylmorpholine. Yields (%) of formate and formamide are shown in blue color. Imidazole (2.5 mmol) as internal standard (I.S.).

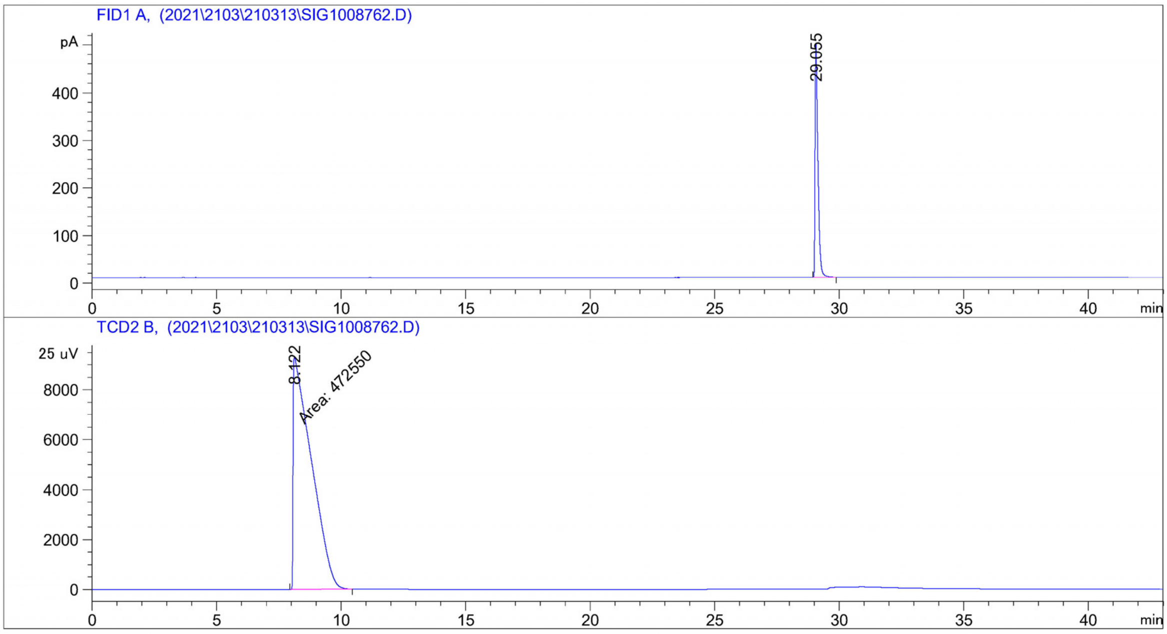


```
=====================================================================
                      External Standard Report
=====================================================================

Sorted By             :      Retention Time
Calib. Data Modified  :      7/27/2022 8:49:02 PM
Multiplier            :      1.0000
Dilution              :      1.0000
Use Multiplier & Dilution Factor with ISTDs

Signal 1: FID1 A,
Signal 2: TCD2 B,

RetTime Sig Type     Area      Amt/Area     Amount    Grp    Name
 [min]                                      [vol%]
-------|--|------|----------|----------|----------|--|---------------
  2.884  2             -          -          -          H2
  8.972  2             -          -          -          Ar
 12.095  2             -          -          -          CO
 21.000  2             -          -          -          CH4
 27.726  2             -          -          -          CO2
```

Figure S12. GC chromatogram of blank reaction in the absence of catalyst. Argon was reported with retention time at 8.122 min.

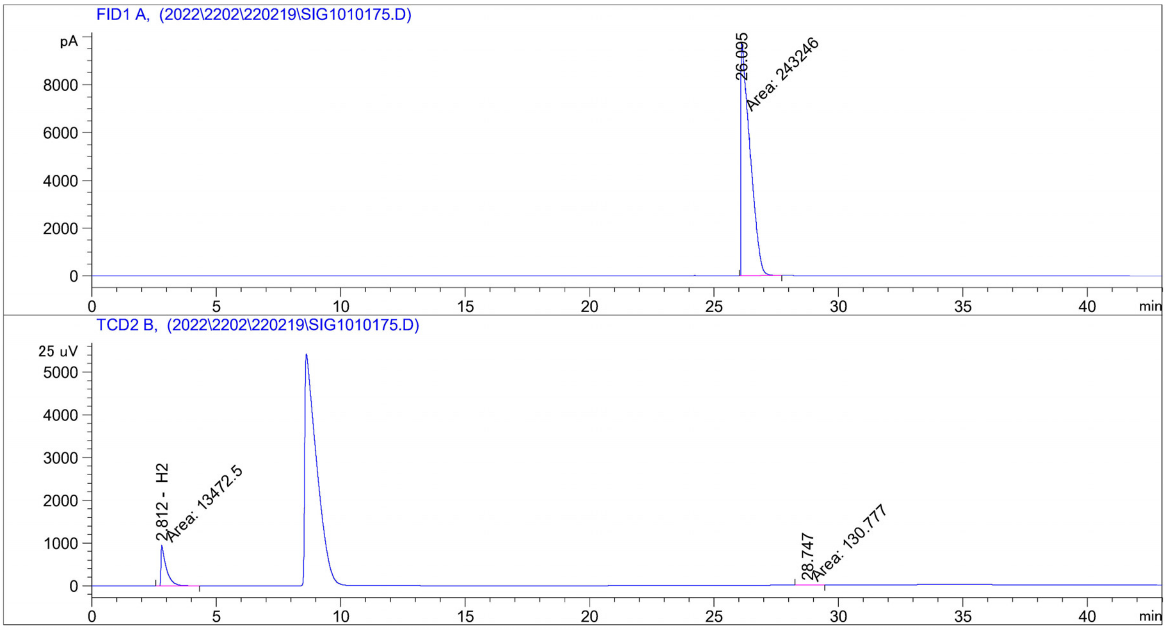

===
External Standard Report
===

Sorted By : Retention Time
Calib. Data Modified : 7/27/2022 8:49:02 PM
Multiplier : 1.0000
Dilution : 1.0000
Use Multiplier & Dilution Factor with ISTDs

Signal 1: FID1 A,
Signal 2: TCD2 B,

RetTime [min]	Sig	Type	Area	Amt/Area	Amount [vol%]	Grp	Name
2.812	2	MM	1.34725e4	4.37829e-3	58.98651		H2
8.972	2		-	-	-		Ar
12.095	2		-	-	-		CO
21.000	2		-	-	-		CH4
27.726	2		-	-	-		CO2

Figure S13. GC chromatogram of hydrogen production (**Fe-1**, F_1, KOH).

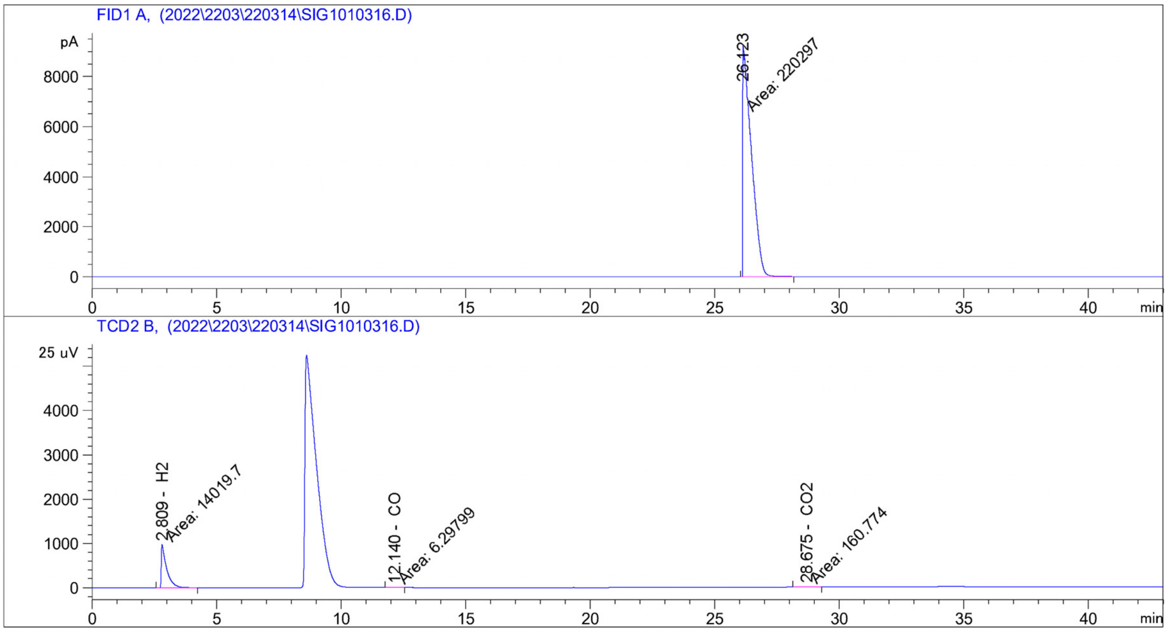


```
=====================================================================
                      External Standard Report
=====================================================================

Sorted By             :      Retention Time
Calib. Data Modified  :      3/14/2022 10:47:01 AM
Multiplier            :      1.0000
Dilution              :      1.0000
Use Multiplier & Dilution Factor with ISTDs

Signal 1: FID1 A,
Signal 2: TCD2 B,

RetTime Sig Type     Area      Amt/Area     Amount   Grp   Name
 [min]                                      [vol%]
-------|--|------|----------|----------|----------|--|---------------
  2.809  2 MM     1.40197e4  4.36818e-3   61.24048     H2
  8.050  2           -          -          -           Ar
 12.140  2 MM       6.29799    0.00000    0.00000      CO
 21.000  2           -          -          -           CH4
 28.675  2 MM     160.77365 1.08608e-4 1.74613e-2      CO2
```

Figure S14. GC chromatogram of hydrogen production (**Fe-2**, F_1, KOH).

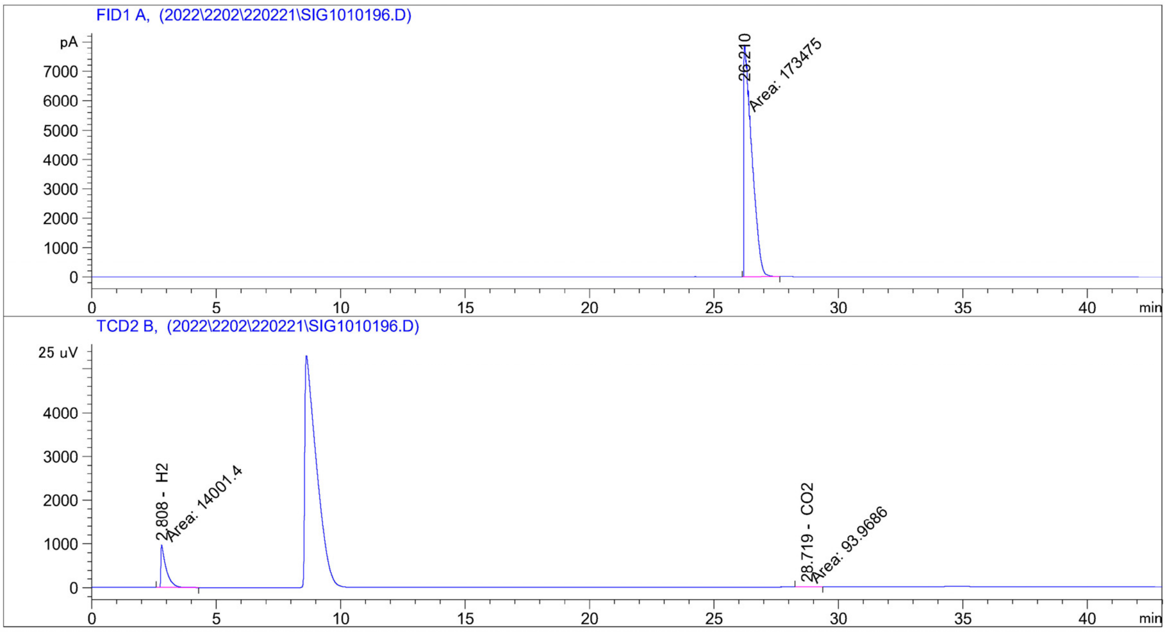


```
=====================================================================
                      External Standard Report
=====================================================================

Sorted By             :      Retention Time
Calib. Data Modified  :      11/6/2022 3:09:06 PM
Multiplier            :      1.0000
Dilution              :      1.0000
Use Multiplier & Dilution Factor with ISTDs

Signal 1: FID1 A,
Signal 2: TCD2 B,

RetTime Sig Type     Area     Amt/Area     Amount   Grp   Name
 [min]                                     [vol%]
-------|--|------|----------|----------|----------|--|---------------
  2.808  2 MM     1.40014e4  4.36852e-3   61.16512    H2
  8.050  2            -          -          -         Ar
 12.162  2            -          -          -         CO
 21.000  2            -          -          -         CH4
 28.719  2 MM       93.96856 7.96793e-5 7.48735e-3    CO2
```

Figure S15. GC chromatogram of hydrogen production (**Fe-3**, F_1, KOH).

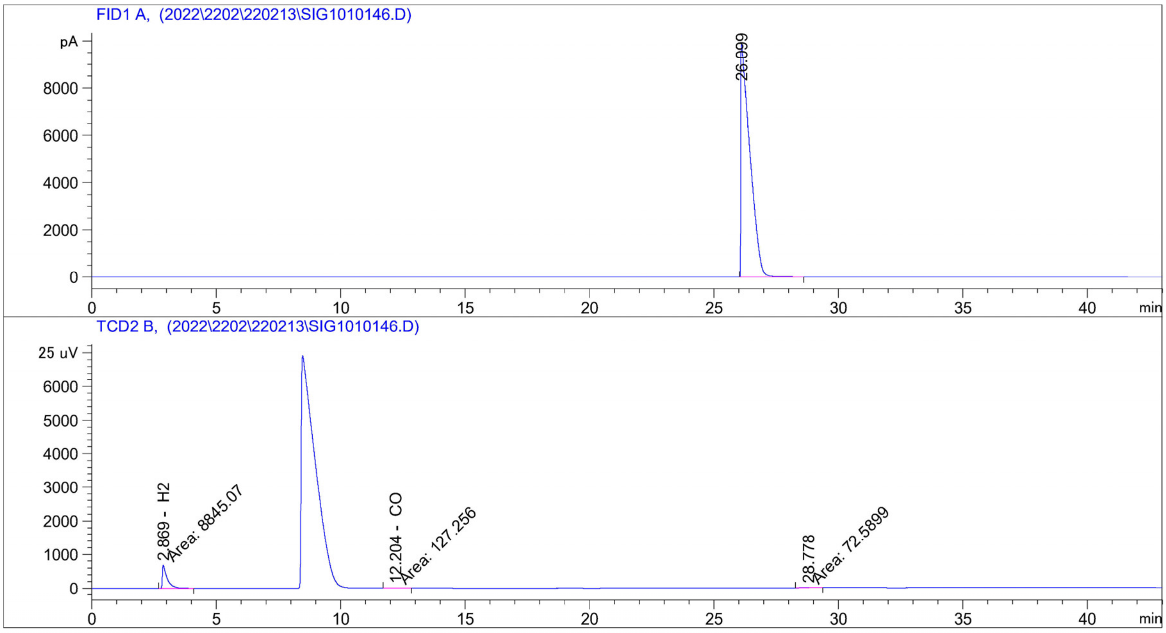


```
=====================================================================
                      External Standard Report
=====================================================================

Sorted By             :      Retention Time
Calib. Data Modified  :      7/27/2022 8:49:02 PM
Multiplier            :      1.0000
Dilution              :      1.0000
Use Multiplier & Dilution Factor with ISTDs

Signal 1: FID1 A,
Signal 2: TCD2 B,

RetTime Sig Type     Area      Amt/Area     Amount   Grp   Name
 [min]                                      [vol%]
-------|--|------|----------|----------|----------|--|---------------
  2.869  2 MM     8845.06543 4.46788e-3   39.51869    H2
  8.972  2             -          -          -        Ar
 12.204  2 MM      127.25586 1.79984e-4 2.29041e-2    CO
 21.000  2             -          -          -        CH4
 27.726  2             -          -          -        CO2
```

Figure S16. GC chromatogram of hydrogen production (**Mn-1**, F_1, KOH).

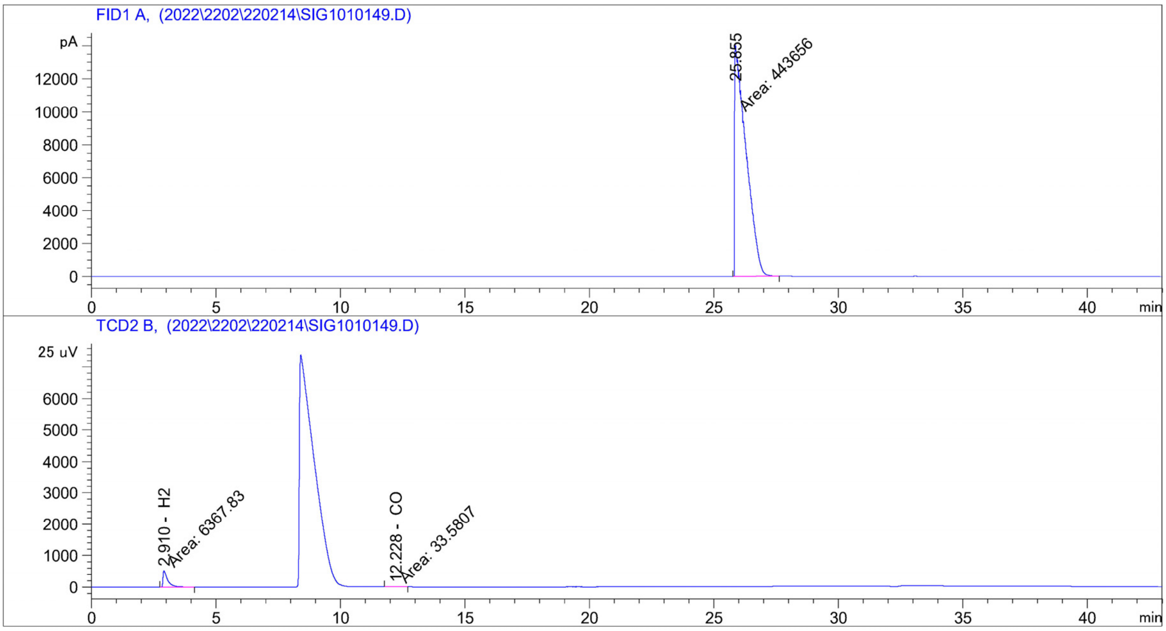

===
External Standard Report
===

```
Sorted By             :      Retention Time
Calib. Data Modified  :      11/6/2022 3:15:45 PM
Multiplier            :      1.0000
Dilution              :      1.0000
Use Multiplier & Dilution Factor with ISTDs

Signal 1: FID1 A,
Signal 2: TCD2 B,
```

RetTime [min]	Sig	Type	Area	Amt/Area	Amount [vol%]	Grp	Name
2.910	2	MM	6367.83154	4.51936e-3	28.77852		H2
8.050	2		-	-	-		Ar
12.228	2	MM	33.58073	7.07227e-5	2.37492e-3		CO
21.000	2		-	-	-		CH4
28.719	2		-	-	-		CO2

Figure S17. GC chromatogram of hydrogen production (**Mn-2**, F_1, KOH).

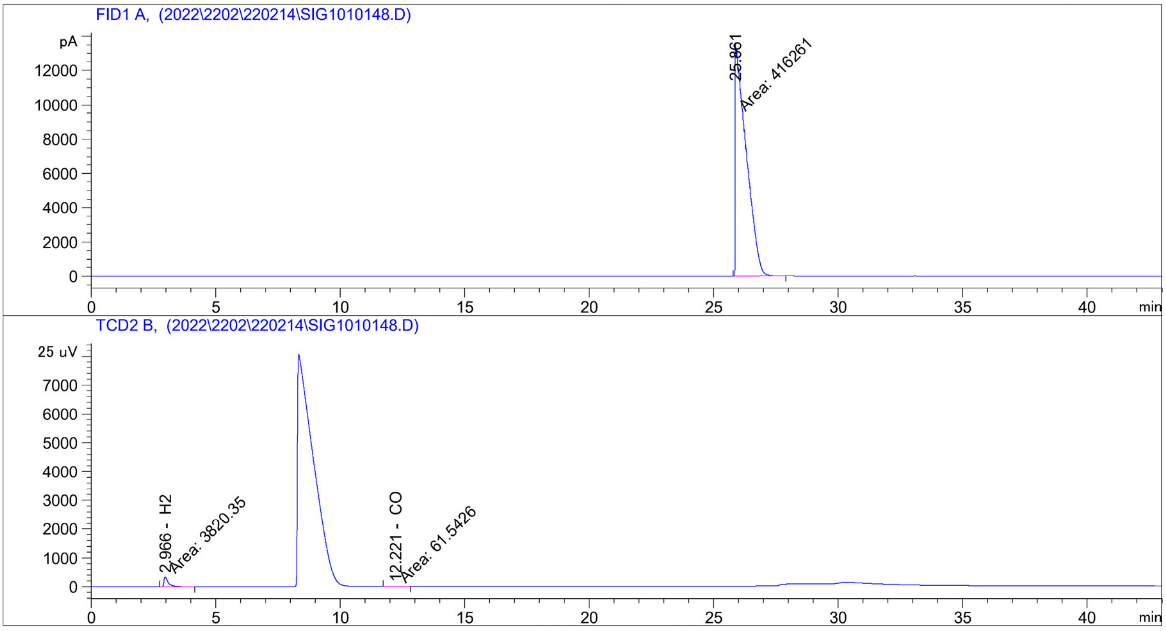


```
=====================================================================
                      External Standard Report
=====================================================================

Sorted By             :      Retention Time
Calib. Data Modified  :      11/6/2022 3:18:27 PM
Multiplier            :      1.0000
Dilution              :      1.0000
Use Multiplier & Dilution Factor with ISTDs

Signal 1: FID1 A,
Signal 2: TCD2 B,

RetTime Sig Type     Area      Amt/Area     Amount   Grp   Name
 [min]                                      [vol%]
-------|--|------|----------|----------|----------|--|---------------
  2.966  2 MM     3820.35205 4.57604e-3   17.48207     H2
  8.050  2             -          -          -         Ar
 12.221  2 MM       61.54265 1.38166e-4 8.50309e-3     CO
 21.000  2             -          -          -         CH4
 28.719  2             -          -          -         CO2
```

Figure S18. GC chromatogram of hydrogen production (**Mn-3**, F_1, KOH).

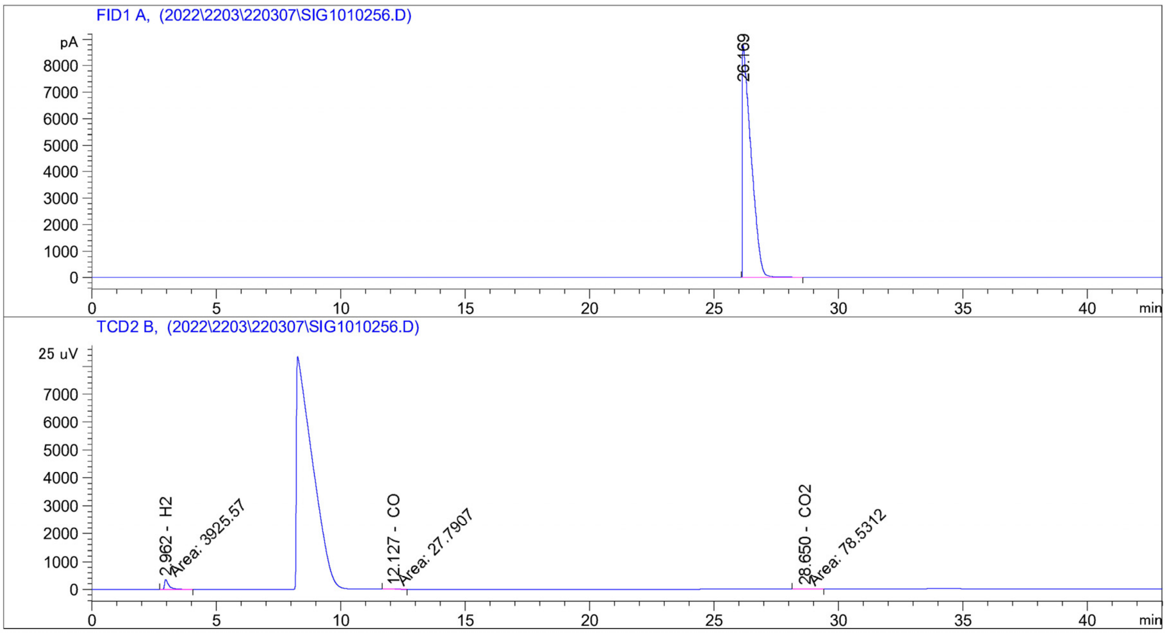

===

External Standard Report

===

Sorted By : Retention Time
Calib. Data Modified : 3/7/2022 5:14:21 PM
Multiplier : 1.0000
Dilution : 1.0000
Use Multiplier & Dilution Factor with ISTDs

Signal 1: FID1 A,
Signal 2: TCD2 B,

RetTime [min]	Sig	Type	Area	Amt/Area	Amount [vol%]	Grp	Name
2.962	2	MM	3925.57129	4.57358e-3	17.95393		H2
8.050	2		-	-	-		Ar
12.127	2	MM	27.79072	3.97956e-5	1.10595e-3		CO
21.000	2		-	-	-		CH4
28.650	2	MM	78.53123	6.59939e-5	5.18258e-3		CO2

Figure S19. GC chromatogram of hydrogen production (**Fe-1**, F_1, Lys).

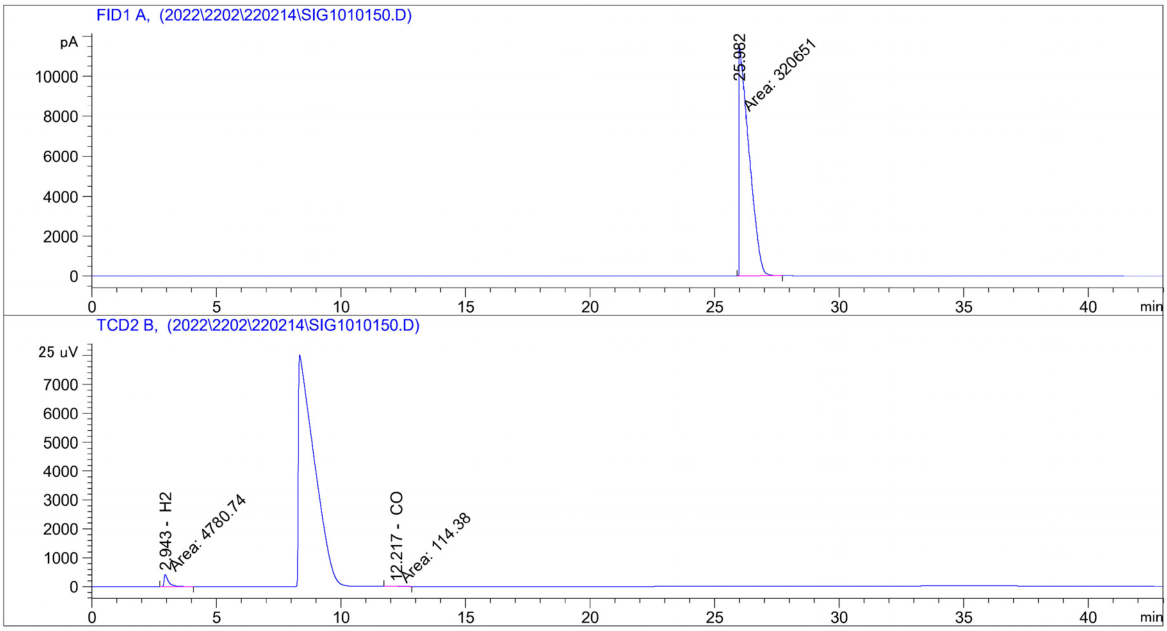

===

External Standard Report

===

Sorted By : Retention Time
Calib. Data Modified : 11/16/2022 10:24:45 AM
Multiplier : 1.0000
Dilution : 1.0000
Use Multiplier & Dilution Factor with ISTDs

Signal 1: FID1 A,
Signal 2: TCD2 B,

RetTime [min]	Sig	Type	Area	Amt/Area	Amount [vol%]	Grp	Name
2.943	2	MM	4780.73535	4.55408e-3	21.77185		H2
8.050	2		-	-	-		Ar
12.217	2	MM	114.38049	1.75576e-4	2.00825e-2		CO
21.000	2		-	-	-		CH4
27.940	2		-	-	-		CO2

Figure S20. GC chromatogram of hydrogen production (**Fe-1**, F_1, Arg).

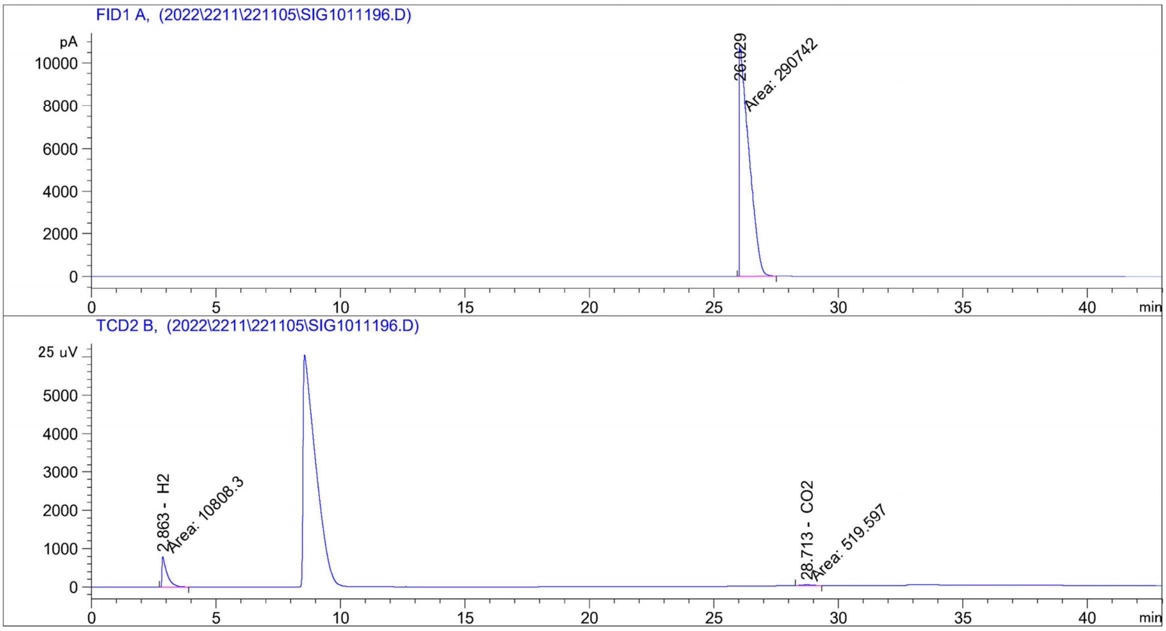


```
=====================================================================
                       External Standard Report
=====================================================================

Sorted By             :      Retention Time
Calib. Data Modified  :      11/5/2022 12:48:11 PM
Multiplier            :      1.0000
Dilution              :      1.0000
Use Multiplier & Dilution Factor with ISTDs

Signal 1: FID1 A,
Signal 2: TCD2 B,

RetTime Sig Type     Area      Amt/Area     Amount   Grp   Name
 [min]                                      [vol%]
-------|--|------|----------|----------|----------|--|---------------
  2.863  2 MM     1.08083e4  4.42893e-3   47.86914     H2
  8.050  2             -          -          -         Ar
 12.175  2             -          -          -         CO
 21.000  2             -          -          -         CH4
 28.713  2 MM      519.59662 1.36714e-4 7.10360e-2     CO2
```

Figure S21. GC chromatogram of hydrogen production (**Fe-1**, MA, KOH).

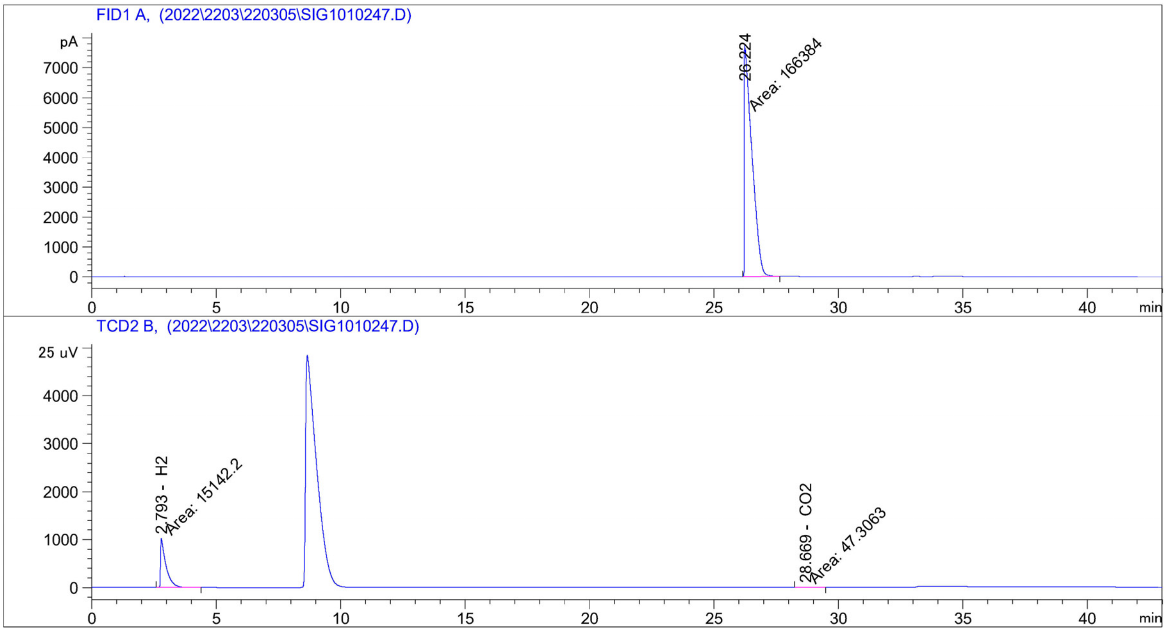


```
=====================================================================
                     External Standard Report
=====================================================================

Sorted By             :      Retention Time
Calib. Data Modified  :      7/27/2022 8:49:02 PM
Multiplier            :      1.0000
Dilution              :      1.0000
Use Multiplier & Dilution Factor with ISTDs

Signal 1: FID1 A,
Signal 2: TCD2 B,

RetTime Sig Type     Area     Amt/Area     Amount   Grp   Name
 [min]                                     [vol%]
-------|--|------|----------|----------|----------|--|---------------
  2.793  2 MM     1.51422e4  4.34773e-3   65.83434     H2
  8.972  2           -          -            -         Ar
 12.095  2           -          -            -         CO
 21.000  2           -          -            -         CH4
 28.669  2 MM       47.30633 1.10087e-5 5.20780e-4     CO2
```

Figure S22. GC chromatogram of hydrogen production (**Fe-1**, DMF, KOH).

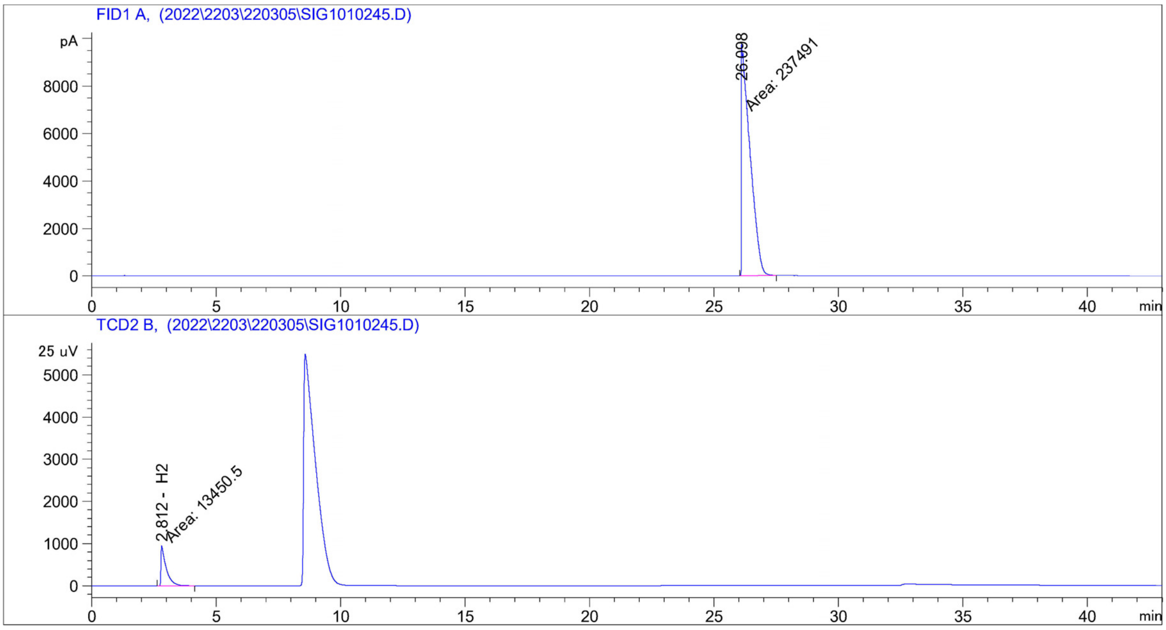

===

External Standard Report

===

Sorted By : Retention Time
Calib. Data Modified : 7/27/2022 8:49:02 PM
Multiplier : 1.0000
Dilution : 1.0000
Use Multiplier & Dilution Factor with ISTDs

Signal 1: FID1 A,
Signal 2: TCD2 B,

RetTime [min]	Sig	Type	Area	Amt/Area	Amount [vol%]	Grp	Name
2.812	2	MM	1.34505e4	4.37870e-3	58.89559		H2
8.972	2		-	-	-		Ar
12.095	2		-	-	-		CO
21.000	2		-	-	-		CH4
27.726	2		-	-	-		CO2

Figure S23. GC chromatogram of hydrogen production (**Fe-1**, F_2, KOH).

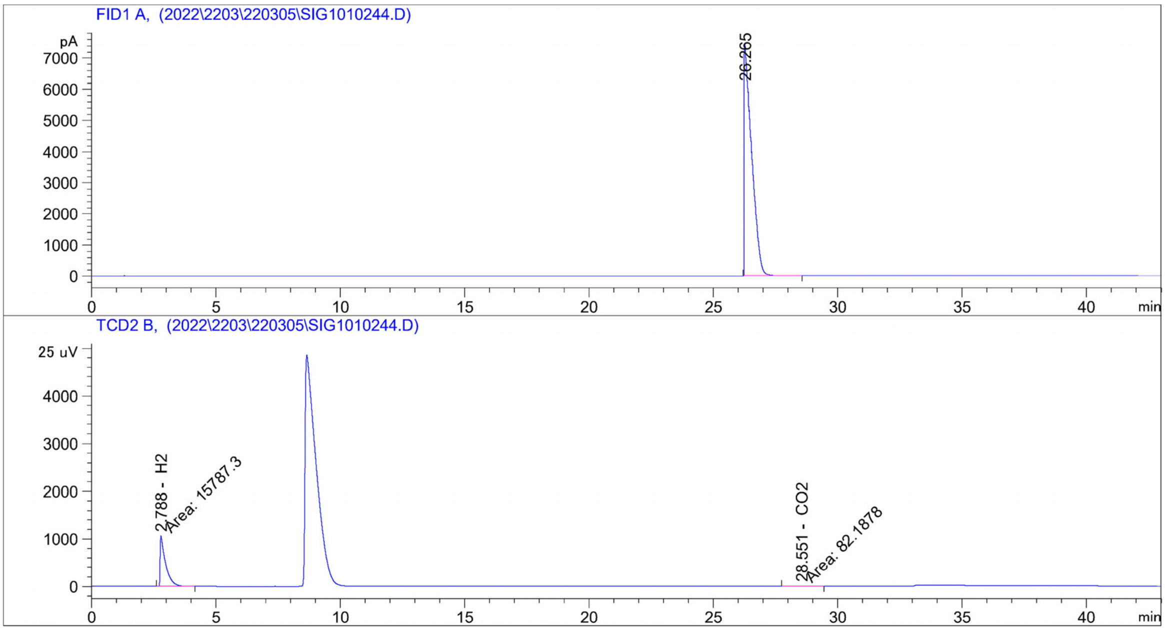


```
=====================================================================
                      External Standard Report
=====================================================================

Sorted By             :      Retention Time
Calib. Data Modified  :      7/27/2022 8:49:02 PM
Multiplier            :      1.0000
Dilution              :      1.0000
Use Multiplier & Dilution Factor with ISTDs

Signal 1: FID1 A,
Signal 2: TCD2 B,
```

RetTime [min]	Sig	Type	Area	Amt/Area	Amount [vol%]	Grp	Name
2.788	2	MM	1.57873e4	4.33615e-3	68.45590		H2
8.972	2		-	-	-		Ar
12.095	2		-	-	-		CO
21.000	2		-	-	-		CH4
28.551	2	MM	82.18775	6.97001e-5	5.72849e-3		CO2

Figure S24. GC chromatogram of hydrogen production (**Fe-1**, F_3, KOH).

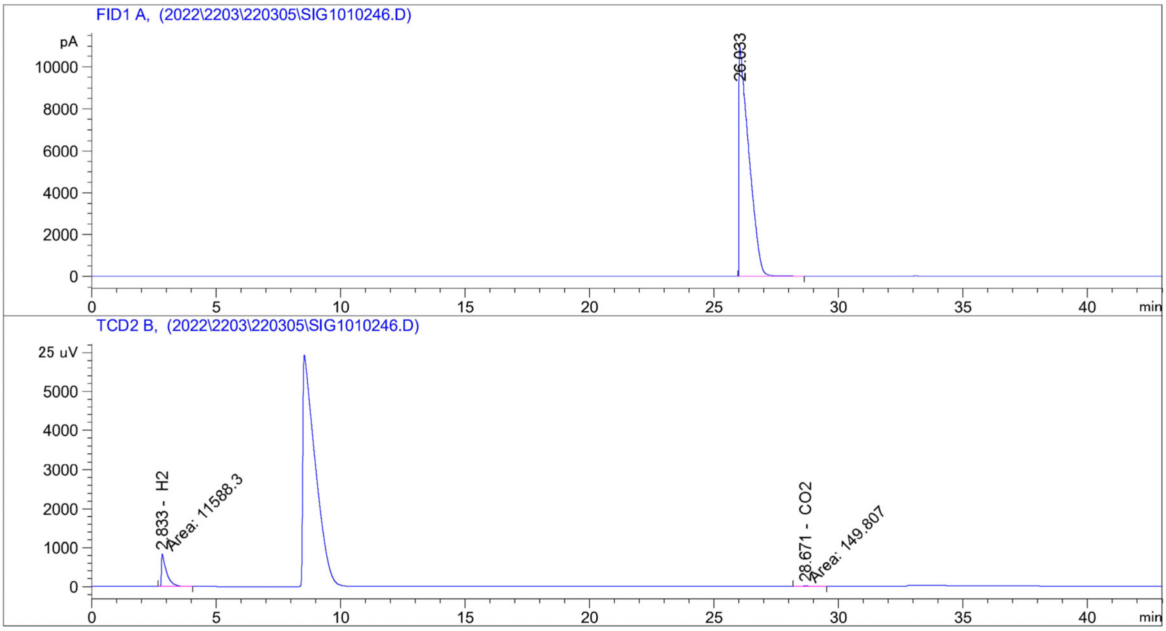

===

External Standard Report

===

Sorted By : Retention Time
Calib. Data Modified : 3/6/2022 10:12:49 AM
Multiplier : 1.0000
Dilution : 1.0000
Use Multiplier & Dilution Factor with ISTDs

Signal 1: FID1 A,
Signal 2: TCD2 B,

RetTime [min]	Sig	Type	Area	Amt/Area	Amount [vol%]	Grp	Name
2.833	2	MM	1.15883e4	4.41385e-3	51.14921		H2
8.050	2		-	-	-		Ar
12.147	2		-	-	-		CO
21.000	2		-	-	-		CH4
28.671	2	MM	149.80696	1.05629e-4	1.58240e-2		CO2

Figure S25. GC chromatogram of hydrogen production (**Fe-1**, F_4, KOH).

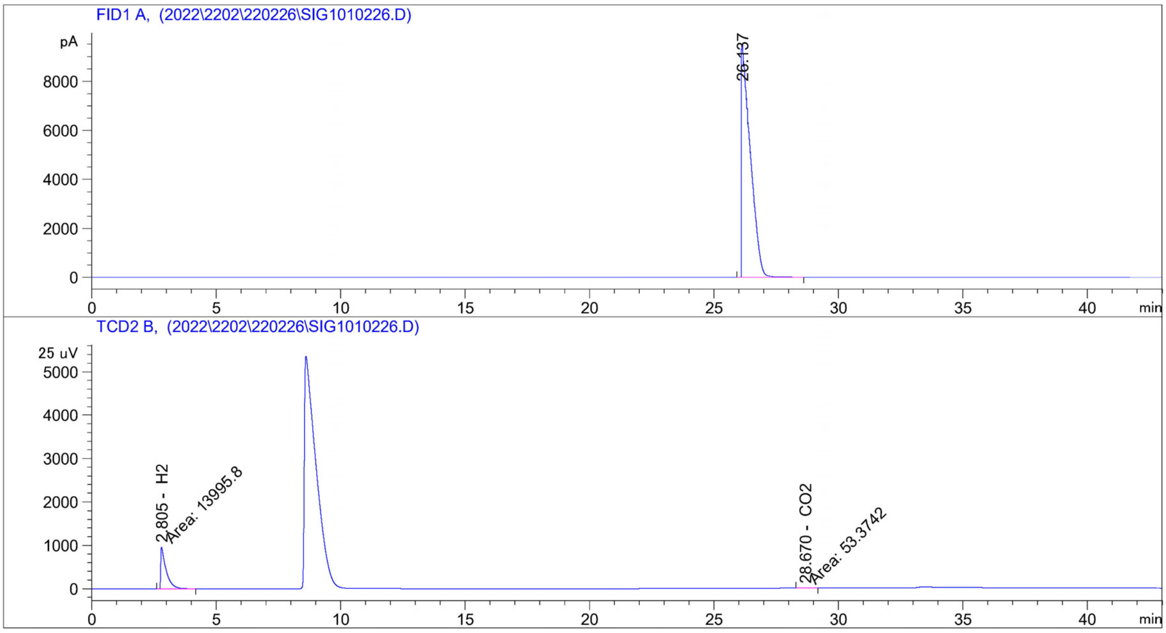


```
=====================================================================
                      External Standard Report
=====================================================================

Sorted By             :      Retention Time
Calib. Data Modified  :      7/27/2022 8:49:02 PM
Multiplier            :      1.0000
Dilution              :      1.0000
Use Multiplier & Dilution Factor with ISTDs

Signal 1: FID1 A,
Signal 2: TCD2 B,

RetTime Sig Type    Area      Amt/Area     Amount   Grp   Name
 [min]                                     [vol%]
-------|--|------|----------|----------|----------|--|---------------
  2.805  2 MM    1.39958e4  4.36862e-3   61.14217    H2
  8.972  2          -          -           -         Ar
 12.095  2          -          -           -         CO
 21.000  2          -          -           -         CH4
 28.670  2 MM      53.37418 2.67300e-5 1.42669e-3    CO2
```

Figure S26. GC chromatogram of hydrogen production (**Fe-1**, FA, KOH, A_1).

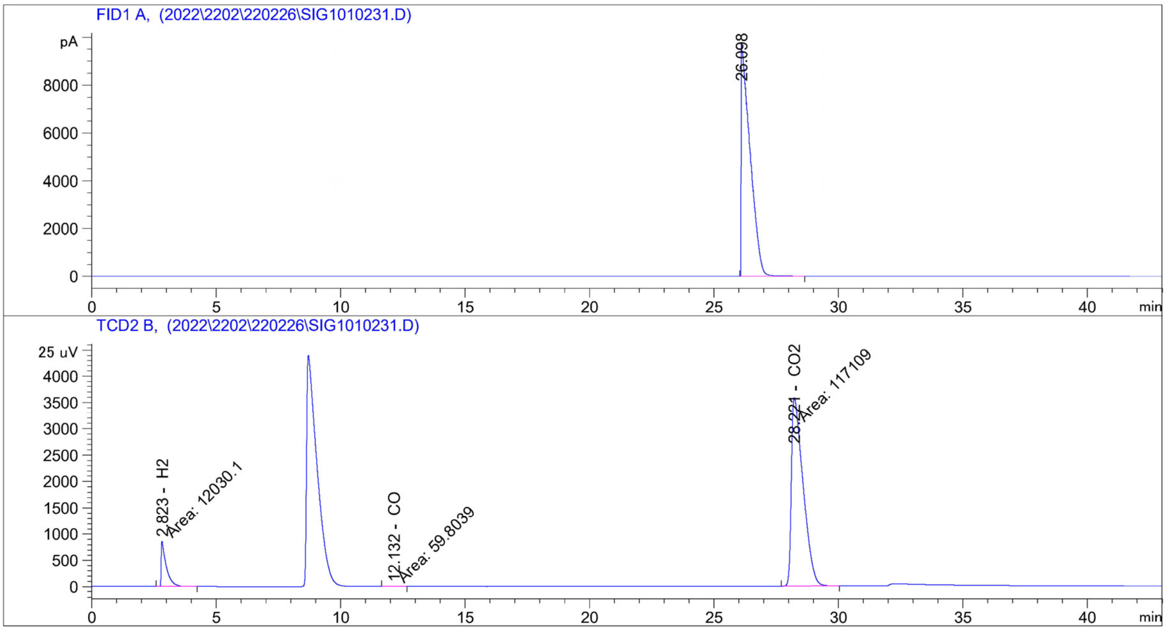


```
===================================================================
                     External Standard Report
===================================================================

Sorted By             :      Retention Time
Calib. Data Modified  :      7/27/2022 8:49:02 PM
Multiplier            :      1.0000
Dilution              :      1.0000
Use Multiplier & Dilution Factor with ISTDs

Signal 1: FID1 A,
Signal 2: TCD2 B,
```

RetTime [min]	Sig	Type	Area	Amt/Area	Amount [vol%]	Grp	Name
2.823	2	MM	1.20301e4	4.40541e-3	52.99741		H2
8.972	2		-	-	-		Ar
12.132	2	MM	59.80387	1.35811e-4	8.12202e-3		CO
21.000	2		-	-	-		CH4
28.221	2	MM	1.17109e5	1.51229e-4	17.71024		CO2

Figure S27. GC chromatogram of hydrogen production (**Fe-1**, FA, A_1).

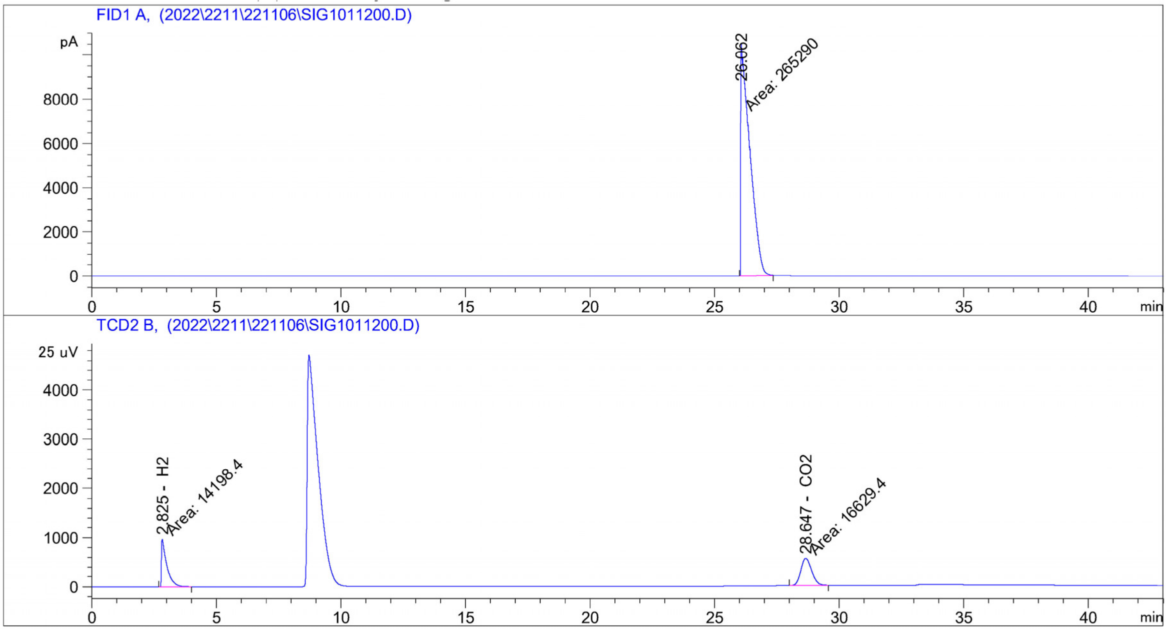


```
=====================================================================
                      External Standard Report
=====================================================================

Sorted By             :      Retention Time
Calib. Data Modified  :      11/6/2022 10:49:01 AM
Multiplier            :      1.0000
Dilution              :      1.0000
Use Multiplier & Dilution Factor with ISTDs

Signal 1: FID1 A,
Signal 2: TCD2 B,

RetTime Sig Type     Area      Amt/Area     Amount   Grp   Name
 [min]                                      [vol%]
-------|--|------|----------|----------|----------|--|---------------
  2.825  2 MM     1.41984e4  4.36490e-3   61.97446     H2
  8.050  2             -          -           -        Ar
 12.162  2             -          -           -        CO
 21.000  2             -          -           -        CH4
 28.647  2 MM     1.66294e4  1.49178e-4    2.48074     CO2
```

Figure S28. GC chromatogram of hydrogen production (**Fe-1**, FA, KOH).

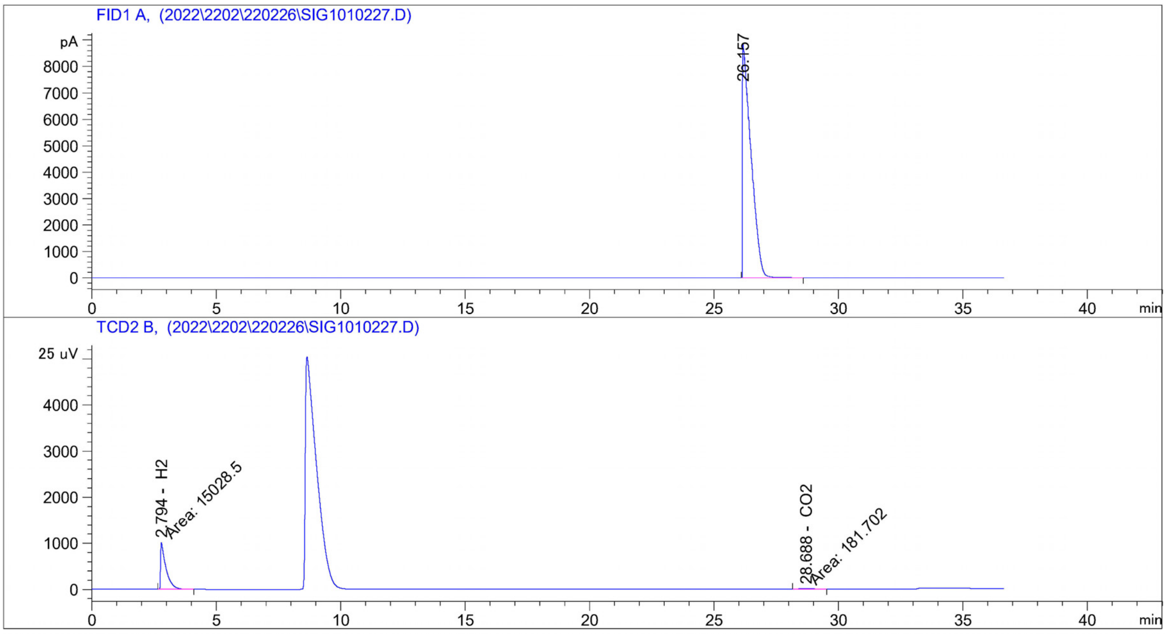

===

External Standard Report

===

Sorted By : Retention Time
Calib. Data Modified : 7/27/2022 8:49:02 PM
Multiplier : 1.0000
Dilution : 1.0000
Use Multiplier & Dilution Factor with ISTDs

Signal 1: FID1 A,
Signal 2: TCD2 B,

RetTime [min]	Sig	Type	Area	Amt/Area	Amount [vol%]	Grp	Name
2.794	2	MM	1.50285e4	4.34978e-3	65.37058		H2
8.972	2		-	-	-		Ar
12.095	2		-	-	-		CO
21.000	2		-	-	-		CH4
28.688	2	MM	181.70160	1.13295e-4	2.05859e-2		CO2

Figure S29. GC chromatogram of hydrogen production (**Fe-1**, $KHCO_2$, A_1).

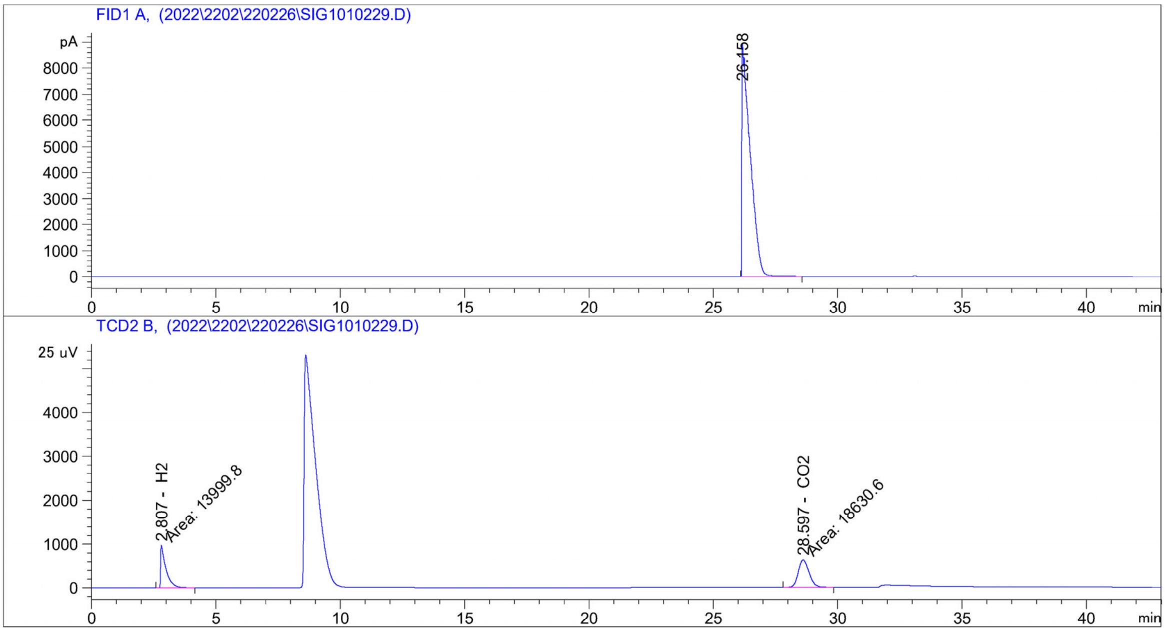


```
=====================================================================
                      External Standard Report
=====================================================================

Sorted By             :      Retention Time
Calib. Data Modified  :      7/27/2022 8:49:02 PM
Multiplier            :      1.0000
Dilution              :      1.0000
Use Multiplier & Dilution Factor with ISTDs

Signal 1: FID1 A,
Signal 2: TCD2 B,

RetTime Sig Type     Area      Amt/Area     Amount    Grp   Name
 [min]                                       [vol%]
-------|--|------|----------|----------|----------|--|---------------
  2.807  2 MM     1.39998e4  4.36854e-3   61.15887     H2
  8.972  2            -          -            -        Ar
 12.095  2            -          -            -        CO
 21.000  2            -          -            -        CH4
 28.597  2 MM     1.86306e4  1.49254e-4    2.78068     CO2
```

Figure S30. GC chromatogram of hydrogen production (**Fe-1**, $KHCO_2$).

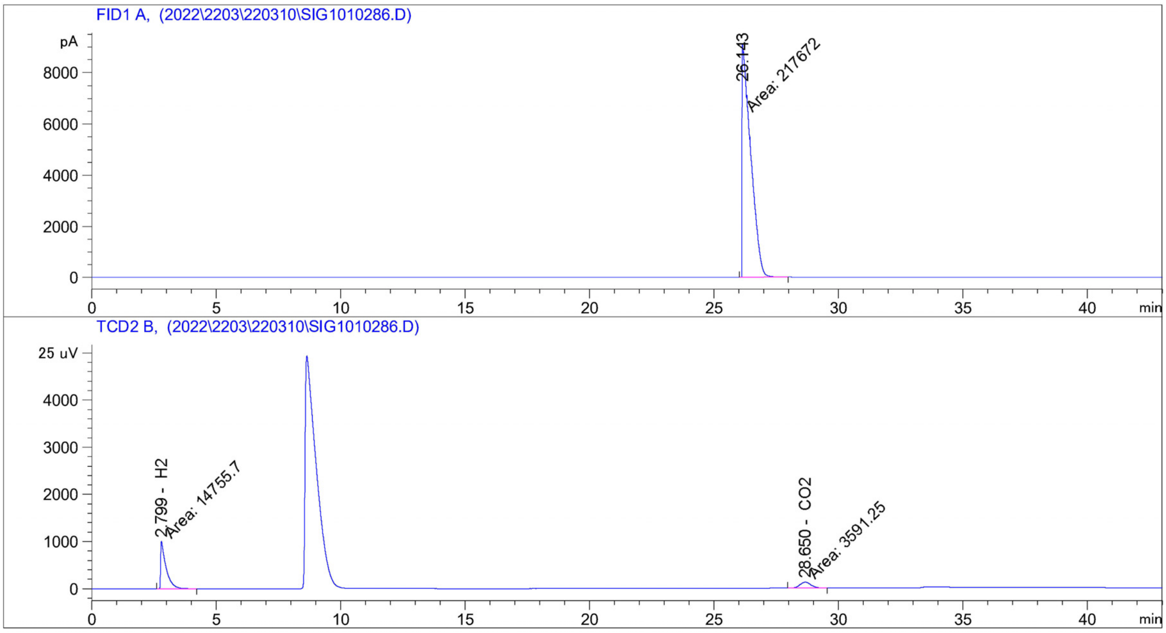


```
=====================================================================
                      External Standard Report
=====================================================================

Sorted By              :      Retention Time
Calib. Data Modified   :      3/11/2022 12:53:19 PM
Multiplier             :      1.0000
Dilution               :      1.0000
Use Multiplier & Dilution Factor with ISTDs

Signal 1: FID1 A,
Signal 2: TCD2 B,

RetTime Sig Type     Area      Amt/Area     Amount    Grp   Name
 [min]                                     [vol%]
-------|--|------|----------|----------|----------|--|---------------
  2.799  2 MM     1.47557e4  4.35473e-3   64.25718     H2
  8.050  2            -          -           -         Ar
 12.150  2            -          -           -         CO
 21.000  2            -          -           -         CH4
 28.650  2 MM     3591.24561 1.47533e-4 5.29828e-1     CO2
```

Figure S31. GC chromatogram of hydrogen production (**Fe-1**, F_1, KOH (75 mol%)).

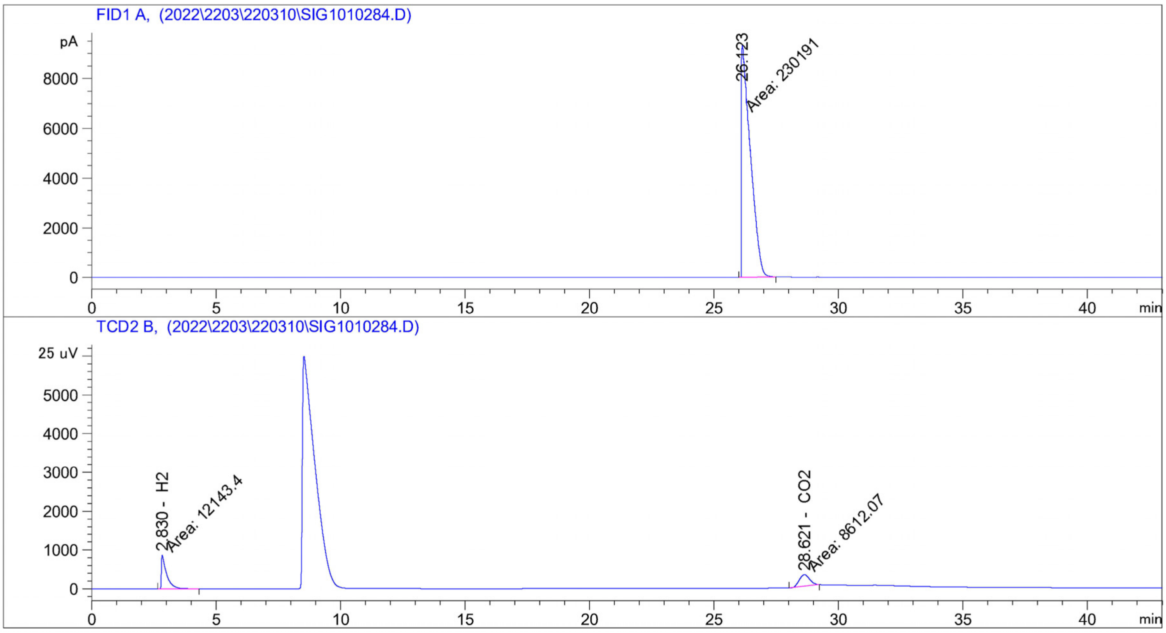


```
=====================================================================
                      External Standard Report
=====================================================================

Sorted By             :      Retention Time
Calib. Data Modified  :      3/11/2022 12:51:17 PM
Multiplier            :      1.0000
Dilution              :      1.0000
Use Multiplier & Dilution Factor with ISTDs

Signal 1: FID1 A,
Signal 2: TCD2 B,

RetTime Sig Type     Area      Amt/Area     Amount   Grp    Name
 [min]                                       [vol%]
-------|--|------|----------|----------|----------|--|---------------
  2.830  2 MM     1.21434e4  4.40325e-3   53.47036     H2
  8.050  2            -          -           -         Ar
 12.150  2            -          -           -         CO
 21.000  2            -          -           -         CH4
 28.621  2 MM     8612.06934 1.48679e-4    1.28043     CO2
```

Figure S32. GC chromatogram of hydrogen production (**Fe-1**, F_1, KOH (50 mol%)).

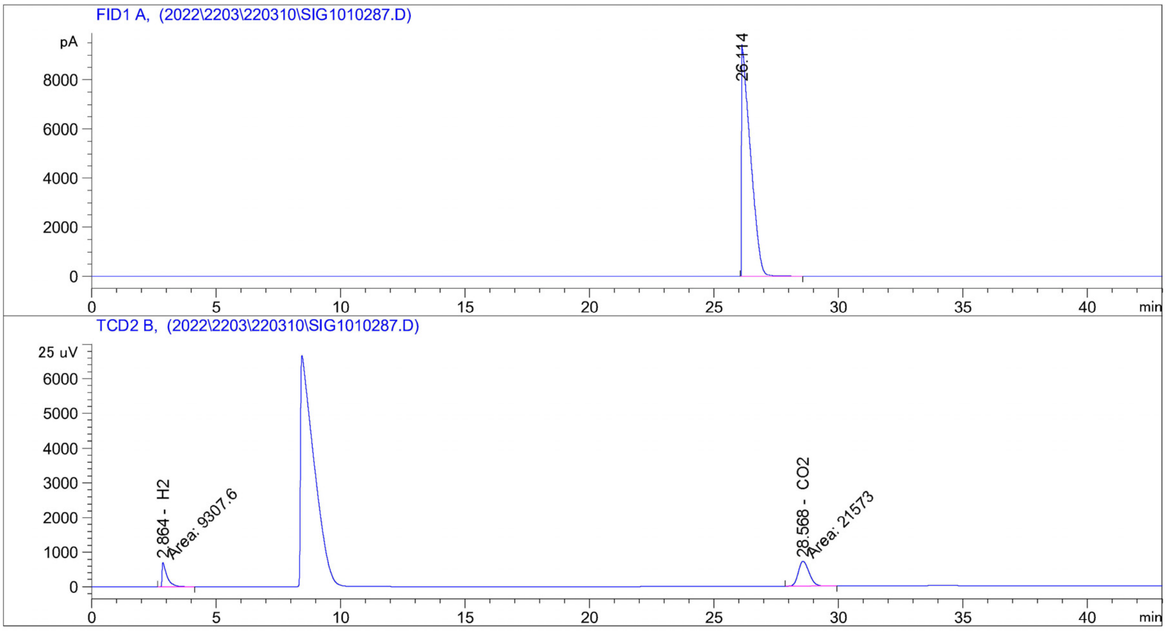

===

External Standard Report

===

Sorted By : Retention Time
Calib. Data Modified : 3/11/2022 12:54:14 PM
Multiplier : 1.0000
Dilution : 1.0000
Use Multiplier & Dilution Factor with ISTDs

Signal 1: FID1 A,
Signal 2: TCD2 B,

RetTime [min]	Sig	Type	Area	Amt/Area	Amount [vol%]	Grp	Name
2.864	2	MM	9307.60254	4.45857e-3	41.49858		H2
8.050	2		-	-	-		Ar
12.150	2		-	-	-		CO
21.000	2		-	-	-		CH4
28.568	2	MM	2.15730e4	1.49351e-4	3.22194		CO2

Figure S33. GC chromatogram of hydrogen production (**Fe-1**, F_1, KOH (25 mol%)).

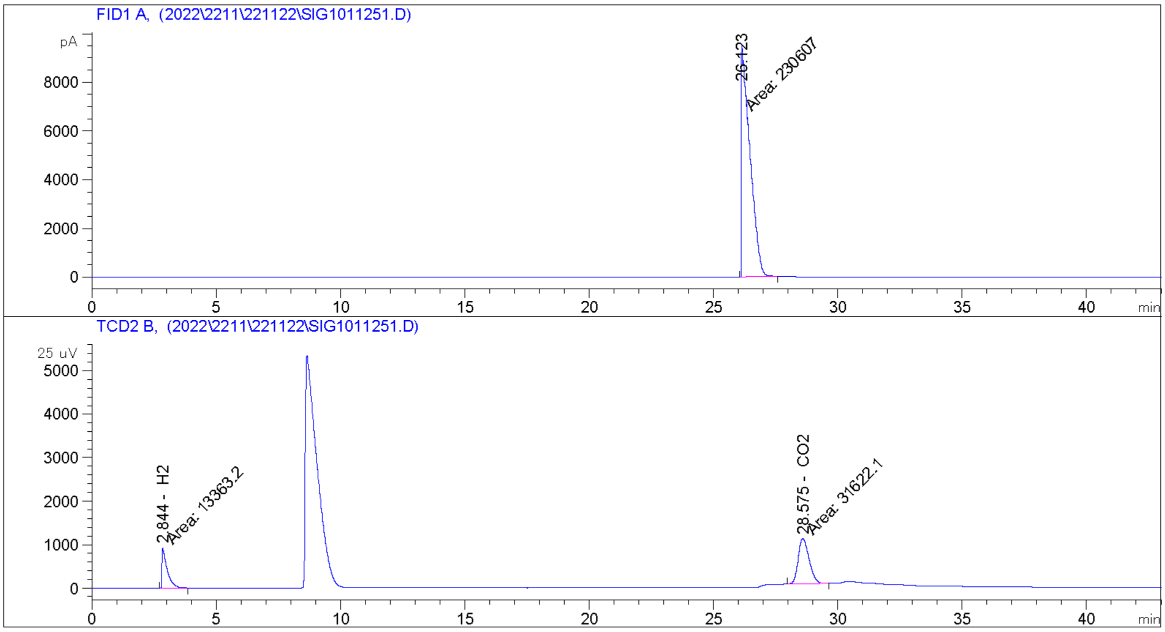


```
=====================================================================
                      External Standard Report
=====================================================================

Sorted By             :      Retention Time
Calib. Data Modified  :      12/4/2022 1:03:40 PM
Multiplier            :      1.0000
Dilution              :      1.0000
Use Multiplier & Dilution Factor with ISTDs

Signal 1: FID1 A,
Signal 2: TCD2 B,
```

RetTime [min]	Sig	Type	Area	Amt/Area	Amount [vol%]	Grp	Name
2.844	2	MM	1.33632e4	4.38032e-3	58.53525		H2
8.050	2		-	-	-		Ar
12.167	2		-	-	-		CO
21.000	2		-	-	-		CH4
28.575	2	MM	3.16221e4	1.49615e-4	4.73115		CO2

Figure S34. GC chromatogram of hydrogen production (**Fe-1**, F_1, KOH, $LiBF_4$ (10 mol%)).

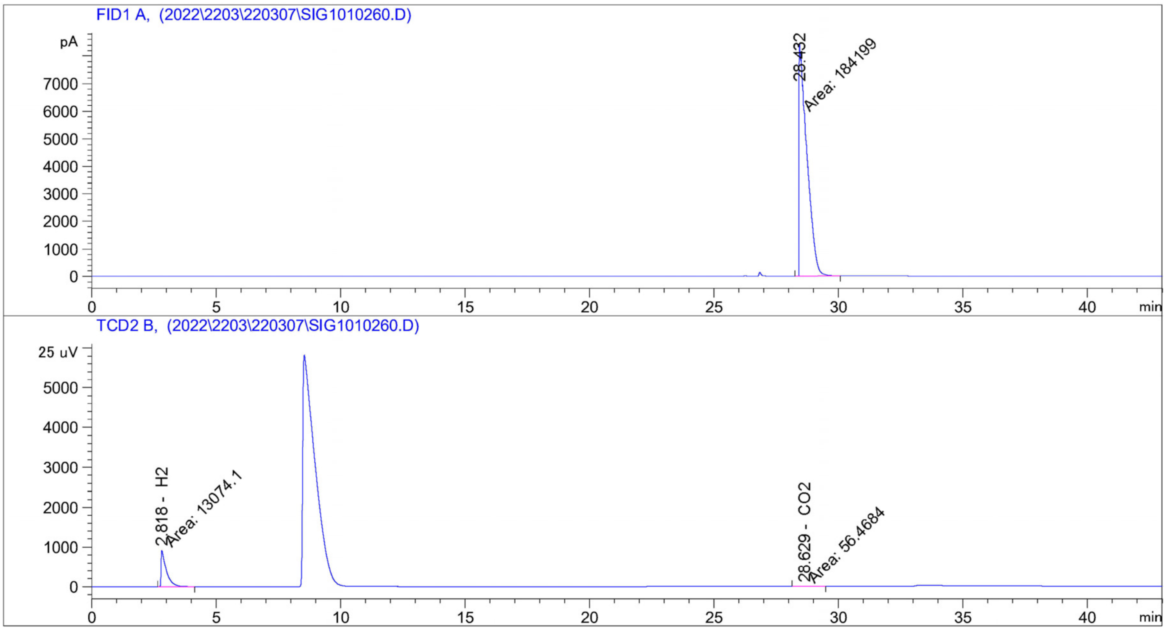

===

External Standard Report

===

Sorted By : Retention Time
Calib. Data Modified : 3/7/2022 5:22:44 PM
Multiplier : 1.0000
Dilution : 1.0000
Use Multiplier & Dilution Factor with ISTDs

Signal 1: FID1 A,
Signal 2: TCD2 B,

RetTime [min]	Sig	Type	Area	Amt/Area	Amount [vol%]	Grp	Name
2.818	2	MM	1.30741e4	4.38571e-3	57.33907		H2
8.050	2		-	-	-		Ar
12.141	2		-	-	-		CO
21.000	2		-	-	-		CH4
28.629	2	MM	56.46844	3.34462e-5	1.88866e-3		CO2

Figure S35. GC chromatogram of hydrogen production (**Fe-1**, F_1, KOH, 2-MTHF/H_2O).

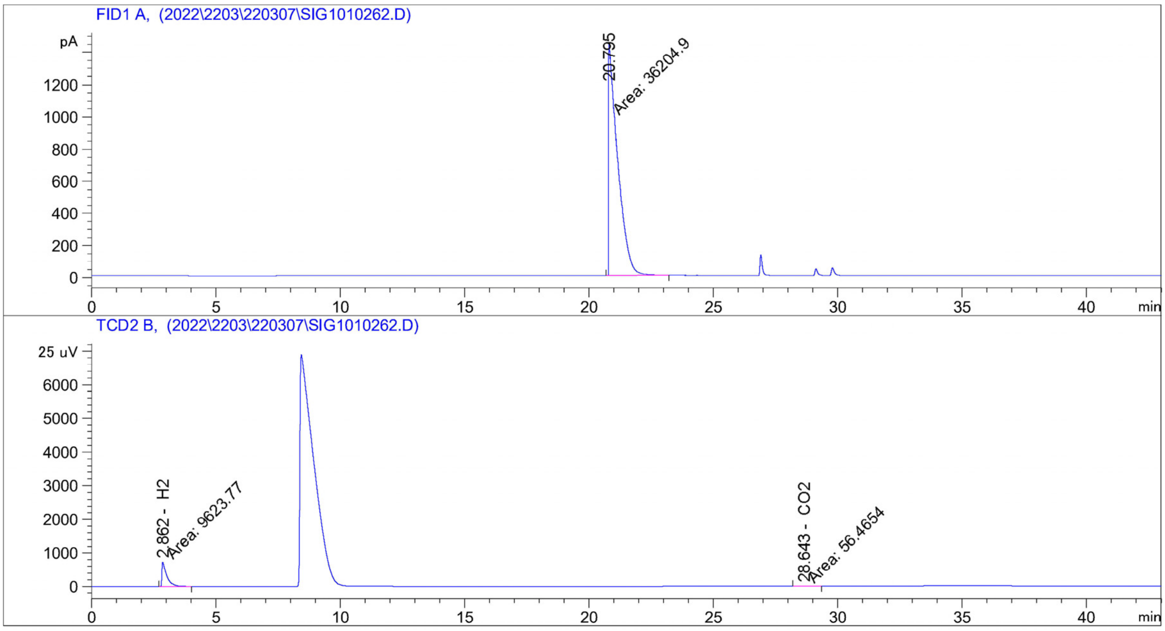

===

External Standard Report

===

Sorted By : Retention Time
Calib. Data Modified : 3/9/2022 11:44:29 AM
Multiplier : 1.0000
Dilution : 1.0000
Use Multiplier & Dilution Factor with ISTDs

Signal 1: FID1 A,
Signal 2: TCD2 B,

RetTime [min]	Sig	Type	Area	Amt/Area	Amount [vol%]	Grp	Name
2.862	2	MM	9623.76758	4.45225e-3	42.84744		H2
8.050	2		-	-	-		Ar
12.103	2		-	-	-		CO
21.000	2		-	-	-		CH4
28.643	2	MM	56.46538	3.34400e-5	1.88820e-3		CO2

Figure S36. GC chromatogram of hydrogen production (**Fe-1**, F_1, KOH, EtOH/H_2O).

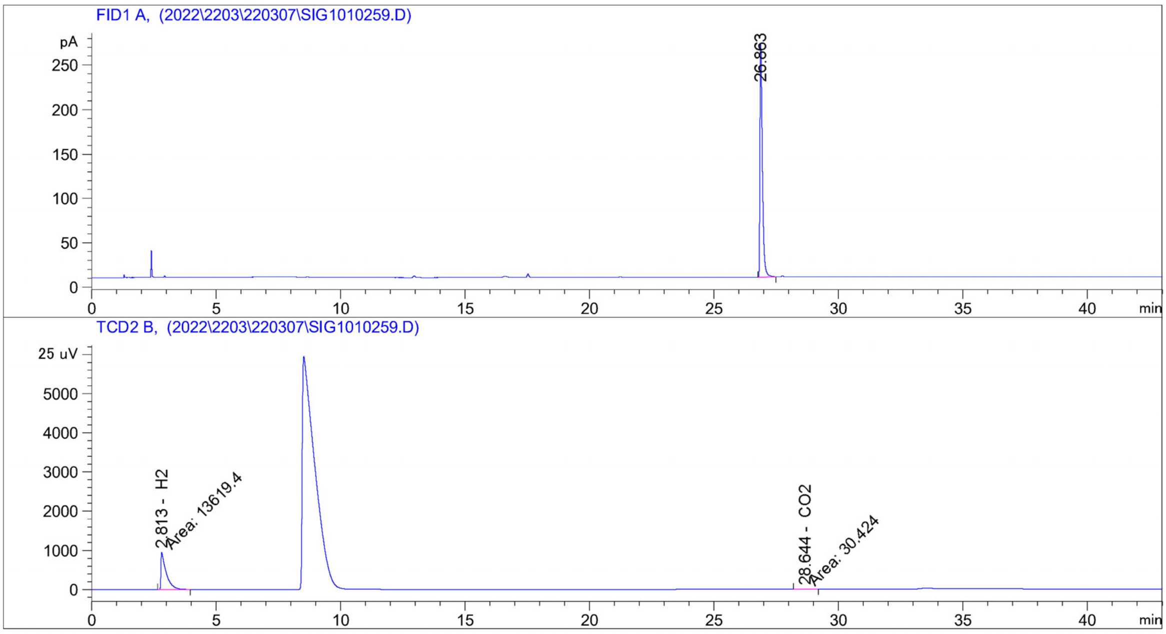

===
External Standard Report
===

Sorted By : Retention Time
Calib. Data Modified : 3/7/2022 5:21:32 PM
Multiplier : 1.0000
Dilution : 1.0000
Use Multiplier & Dilution Factor with ISTDs

Signal 1: FID1 A,
Signal 2: TCD2 B,

RetTime [min]	Sig	Type	Area	Amt/Area	Amount [vol%]	Grp	Name
2.813	2	MM	1.36194e4	4.37557e-3	59.59246		H2
8.050	2		-	-	-		Ar
12.141	2		-	-	-		CO
21.000	2		-	-	-		CH4
28.644	2	MM	30.42404	0.00000	0.00000		CO2

Figure S37. GC chromatogram of hydrogen production (**Fe-1**, F_1, KOH, Tryglyme/H_2O).

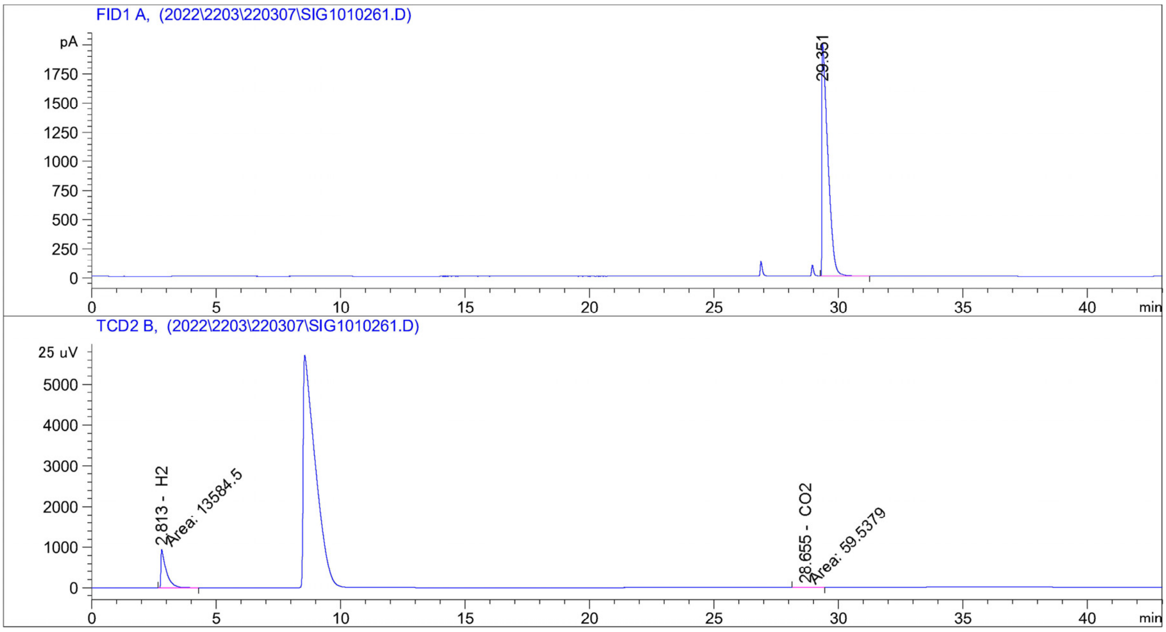


```
=====================================================================
                      External Standard Report
=====================================================================

Sorted By             :      Retention Time
Calib. Data Modified  :      3/7/2022 6:10:18 PM
Multiplier            :      1.0000
Dilution              :      1.0000
Use Multiplier & Dilution Factor with ISTDs

Signal 1: FID1 A,
Signal 2: TCD2 B,

RetTime Sig Type     Area     Amt/Area     Amount    Grp   Name
 [min]                                     [vol%]
-------|--|------|----------|----------|----------|--|---------------
  2.813  2 MM     1.35845e4  4.37621e-3   59.44884     H2
  8.050  2            -          -           -         Ar
 12.141  2            -          -           -         CO
 21.000  2            -          -           -         CH4
 28.655  2 MM       59.53789 3.94189e-5 2.34692e-3     CO2
```

Figure S38. GC chromatogram of hydrogen production (**Fe-1**, F_1, KOH, Dioxane/H_2O).

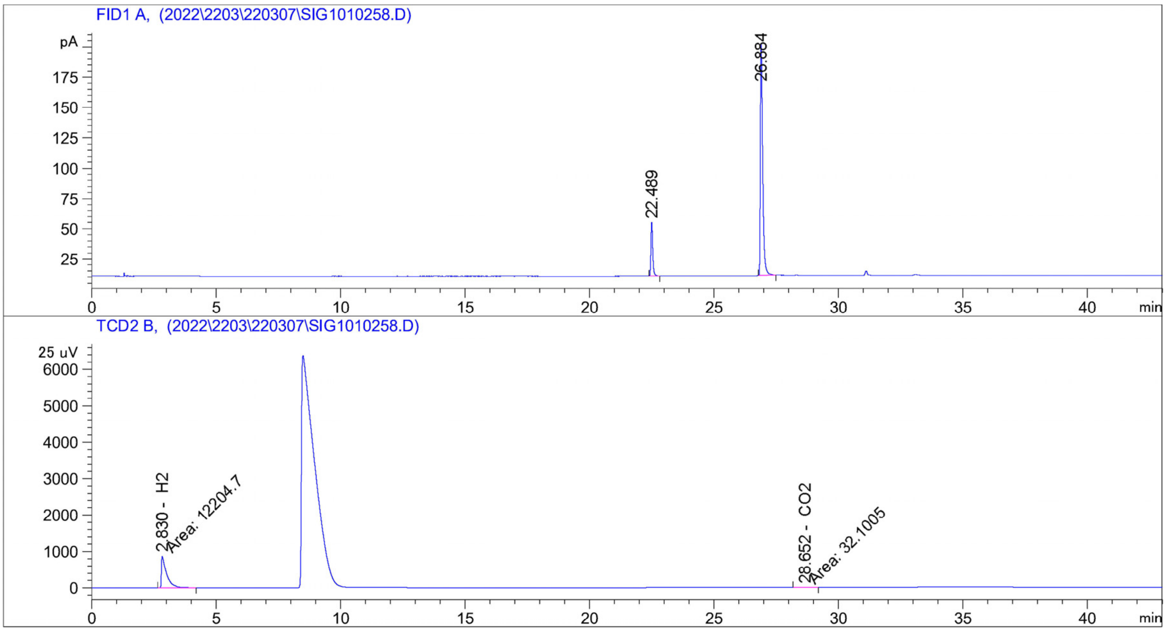

===

External Standard Report

===

Sorted By : Retention Time

Calib. Data Modified : 3/7/2022 5:20:29 PM

Multiplier : 1.0000

Dilution : 1.0000

Use Multiplier & Dilution Factor with ISTDs

Signal 1: FID1 A,

Signal 2: TCD2 B,

RetTime [min]	Sig	Type	Area	Amt/Area	Amount [vol%]	Grp	Name
2.830	2	MM	1.22047e4	4.40209e-3	53.72616		H2
8.050	2		-	-	-		Ar
12.141	2		-	-	-		CO
21.000	2		-	-	-		CH4
28.652	2	MM	32.10046	0.00000	0.00000		CO2

Figure S39. GC chromatogram of hydrogen production (**Fe-1**, F_1, KOH, DMSO /H_2O).

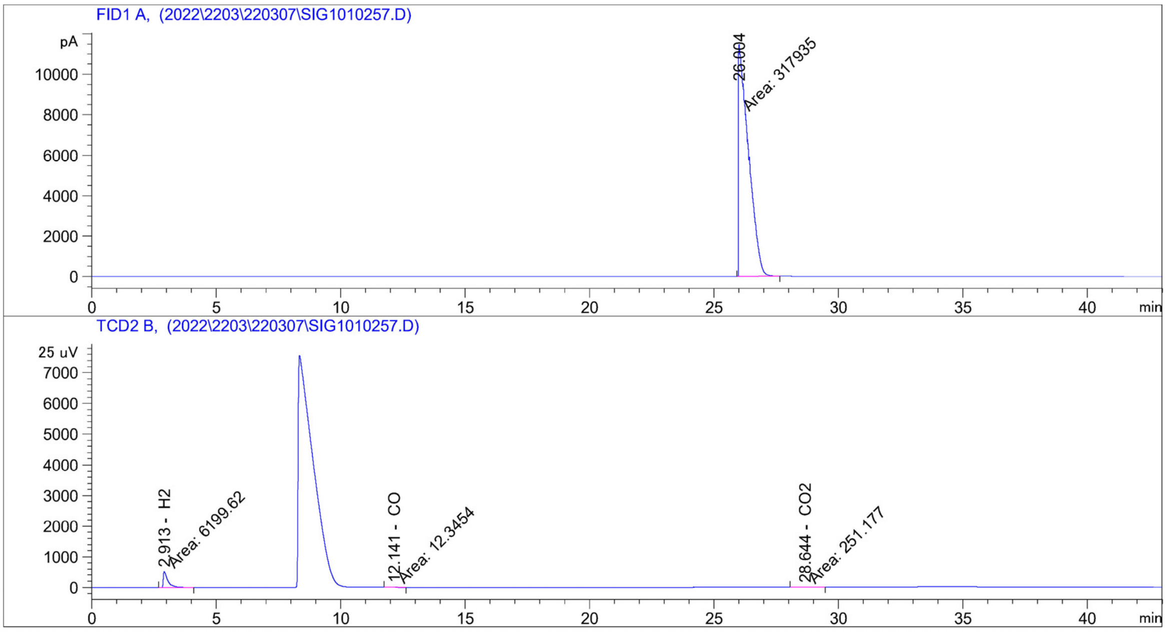


```
=====================================================================
                      External Standard Report
=====================================================================

Sorted By             :      Retention Time
Calib. Data Modified  :      7/27/2022 8:49:02 PM
Multiplier            :      1.0000
Dilution              :      1.0000
Use Multiplier & Dilution Factor with ISTDs

Signal 1: FID1 A,
Signal 2: TCD2 B,
```

RetTime [min]	Sig	Type	Area	Amt/Area	Amount [vol%]	Grp	Name
2.913	2	MM	6199.62305	4.52297e-3	28.04068		H2
8.972	2		-	-	-		Ar
12.141	2	MM	12.34545	0.00000	0.00000		CO
21.000	2		-	-	-		CH4
28.644	2	MM	251.17709	1.23255e-4	3.09588e-2		CO2

Figure S40. GC chromatogram of hydrogen production (**Fe-1**, F_1, KOH, 80 °C).

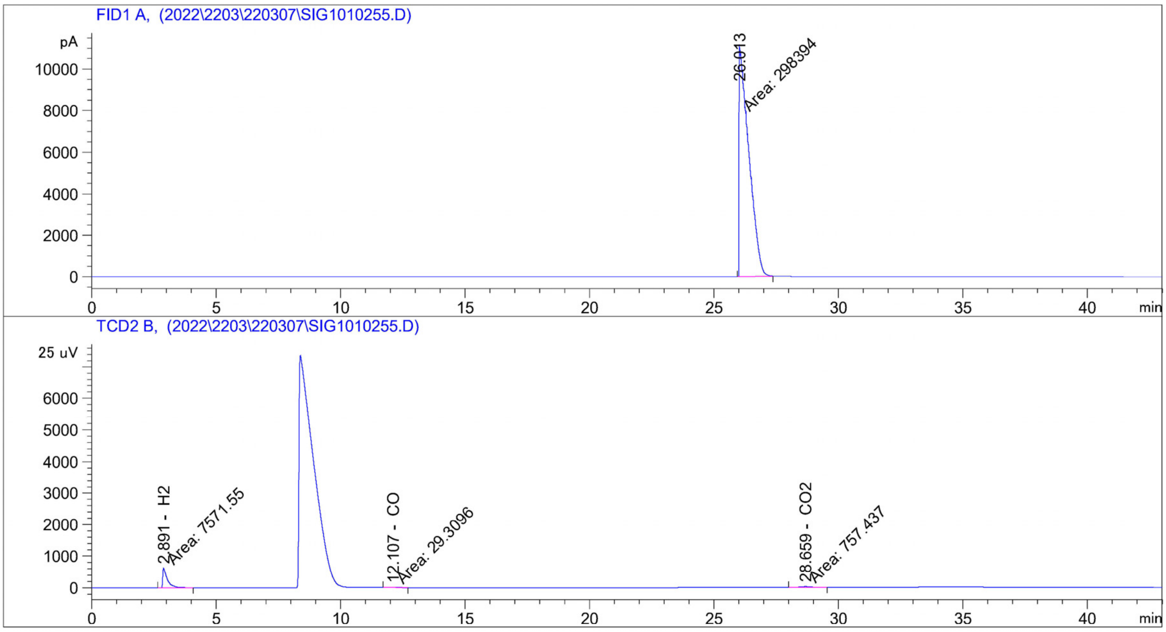


```
=====================================================================
                      External Standard Report
=====================================================================

Sorted By             :      Retention Time
Calib. Data Modified  :      7/27/2022 8:49:02 PM
Multiplier            :      1.0000
Dilution              :      1.0000
Use Multiplier & Dilution Factor with ISTDs

Signal 1: FID1 A,
Signal 2: TCD2 B,
```

RetTime [min]	Sig	Type	Area	Amt/Area	Amount [vol%]	Grp	Name
2.891	2	MM	7571.55420	4.49399e-3	34.02647		H2
8.972	2		-	-	-		Ar
12.107	2	MM	29.30964	4.90912e-5	1.43885e-3		CO
21.000	2		-	-	-		CH4
28.659	2	MM	757.43658	1.40671e-4	1.06549e-1		CO2

Figure S41. GC chromatogram of hydrogen production (**Fe-1**, F_1, KOH, 100 °C).

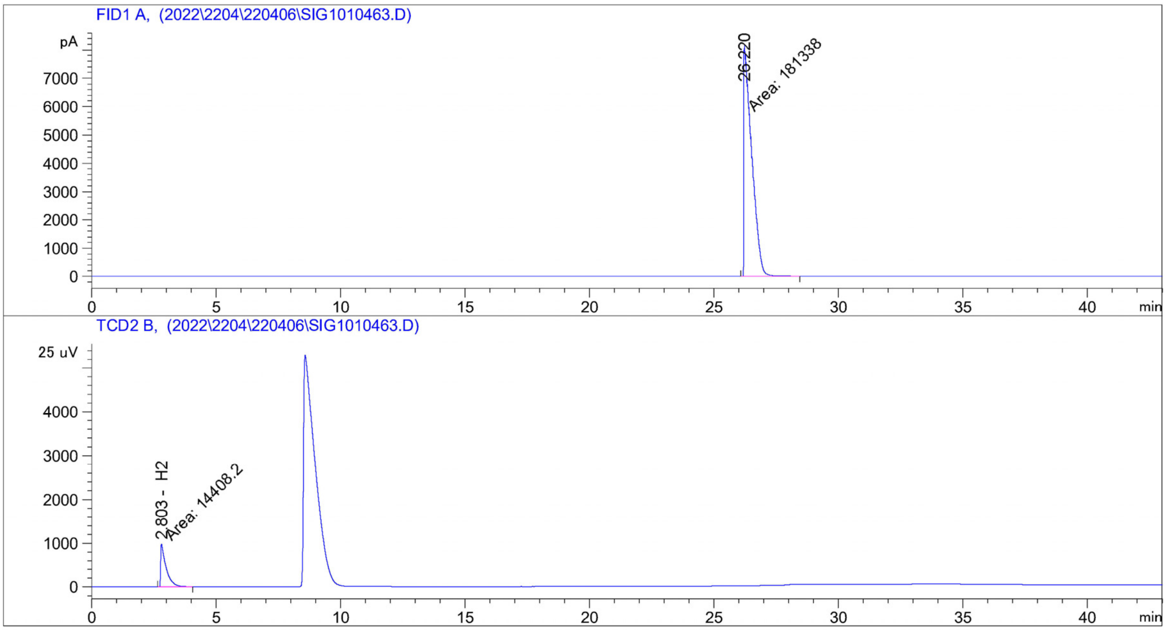


```
=====================================================================
                      External Standard Report
=====================================================================

Sorted By             :      Retention Time
Calib. Data Modified  :      4/6/2022 1:40:28 PM
Multiplier            :      1.0000
Dilution              :      1.0000
Use Multiplier & Dilution Factor with ISTDs

Signal 1: FID1 A,
Signal 2: TCD2 B,

RetTime Sig Type     Area      Amt/Area     Amount    Grp   Name
 [min]                                      [vol%]
-------|--|------|----------|----------|----------|--|---------------
  2.803  2 MM     1.44082e4  4.36106e-3   62.83490     H2
  8.122  2          -          -            -          Ar
 12.120  2          -          -            -          CO
 21.000  2          -          -            -          CH4
 28.644  2          -          -            -          CO2
```

Figure S42. GC chromatogram of hydrogen production (**Fe-1**, $KHCO_2$, A_2).

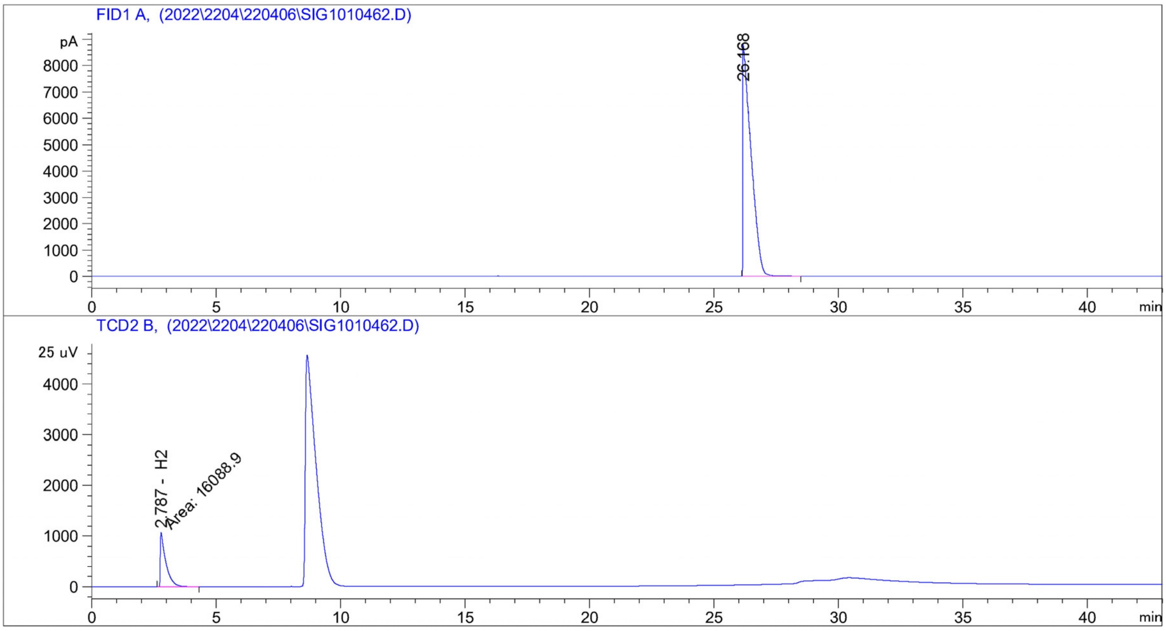


```
=====================================================================
                      External Standard Report
=====================================================================

Sorted By             :      Retention Time
Calib. Data Modified  :      4/6/2022 12:11:42 PM
Multiplier            :      1.0000
Dilution              :      1.0000
Use Multiplier & Dilution Factor with ISTDs

Signal 1: FID1 A,
Signal 2: TCD2 B,

RetTime Sig Type     Area      Amt/Area     Amount    Grp    Name
 [min]                                      [vol%]
-------|--|------|----------|----------|----------|--|---------------
  2.787  2 MM     1.60889e4  4.33078e-3   69.67757     H2
  8.050  2            -          -          -          Ar
 12.098  2            -          -          -          CO
 21.000  2            -          -          -          CH4
 27.901  2            -          -          -          CO2
```

Figure S43. GC chromatogram of hydrogen production (**Fe-1**, $KHCO_2$, A_3).

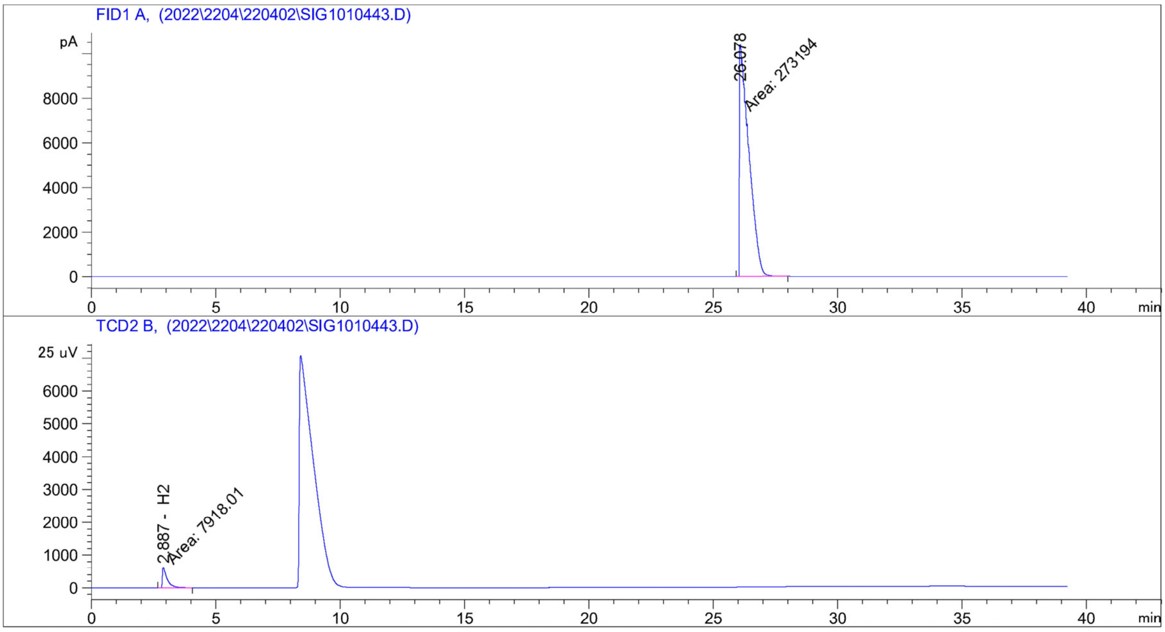

===

External Standard Report

===

Sorted By : Retention Time
Calib. Data Modified : 4/2/2022 3:58:44 PM
Multiplier : 1.0000
Dilution : 1.0000
Use Multiplier & Dilution Factor with ISTDs

Signal 1: FID1 A,
Signal 2: TCD2 B,

RetTime [min]	Sig	Type	Area	Amt/Area	Amount [vol%]	Grp	Name
2.887	2	MM	7918.00879	4.48681e-3	35.52663		H2
8.050	2		-	-	-		Ar
12.106	2		-	-	-		CO
21.000	2		-	-	-		CH4
28.611	2		-	-	-		CO2

Figure S44. GC chromatogram of hydrogen production (**Fe-1**, $KHCO_2$, DBU).

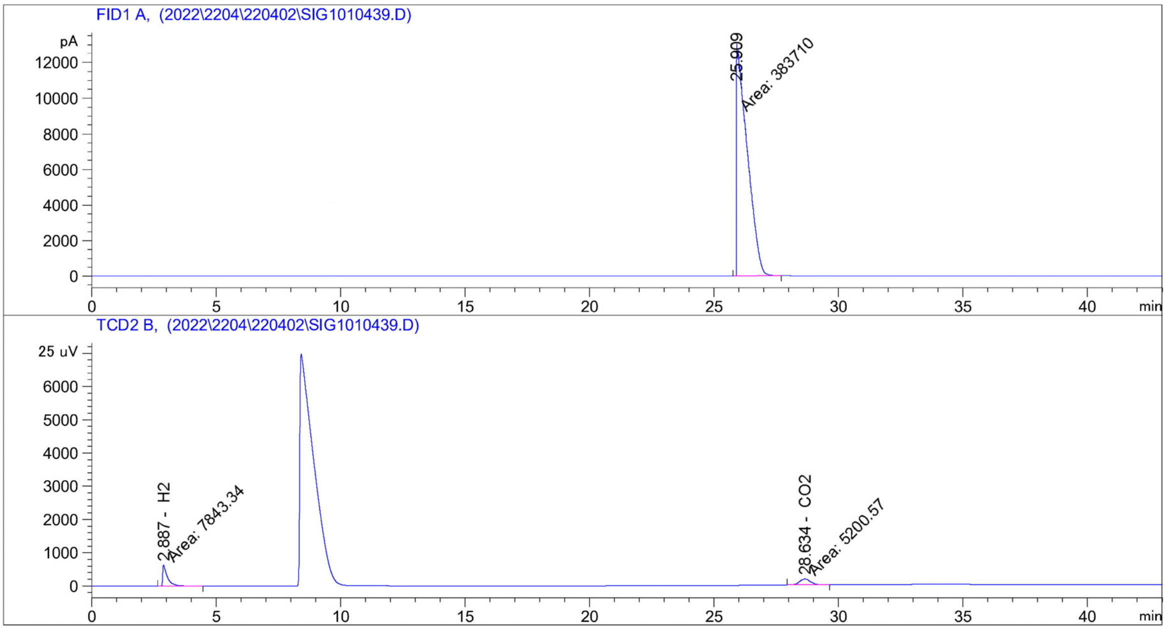


```
=====================================================================
                      External Standard Report
=====================================================================

Sorted By             :      Retention Time
Calib. Data Modified  :      4/2/2022 12:53:07 PM
Multiplier            :      1.0000
Dilution              :      1.0000
Use Multiplier & Dilution Factor with ISTDs

Signal 1: FID1 A,
Signal 2: TCD2 B,

RetTime Sig Type     Area      Amt/Area     Amount    Grp    Name
 [min]                                      [vol%]
-------|--|------|----------|----------|----------|--|---------------
  2.887   2 MM     7843.33984 4.48836e-3   35.20370     H2
  8.050   2             -          -          -         Ar
 12.106   2             -          -          -         CO
 21.000   2             -          -          -         CH4
 28.634   2 MM     5200.57080 1.48124e-4 7.70328e-1     CO2
```

Figure S45. GC chromatogram of hydrogen production (**Fe-1**, $KHCO_2$, DABCO).

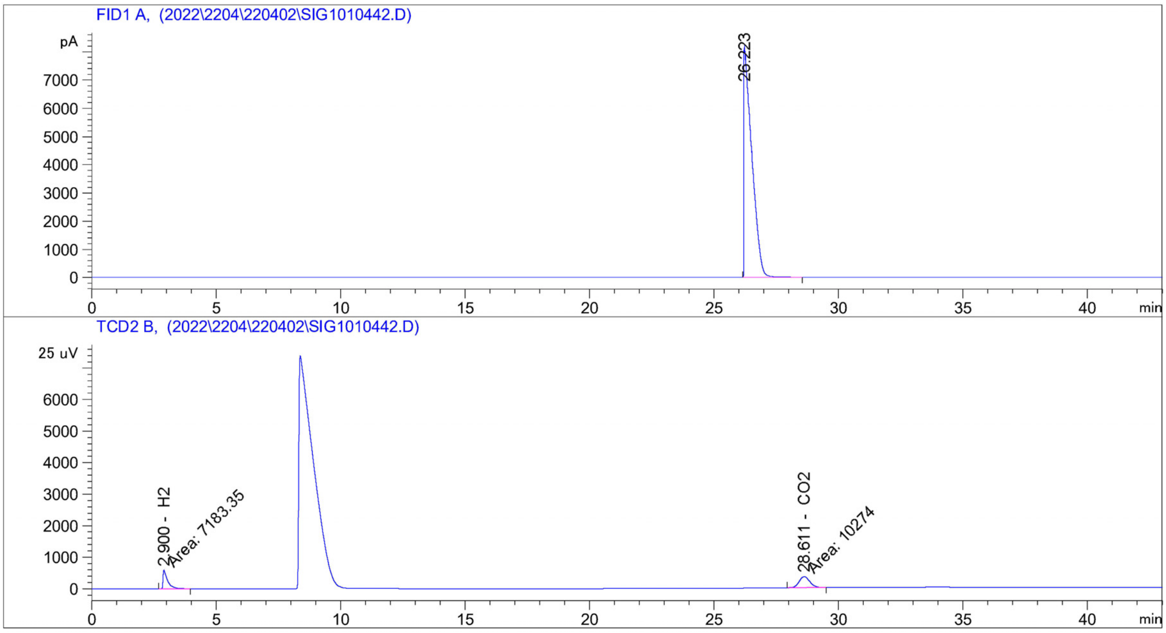

===

External Standard Report

===

Sorted By : Retention Time
Calib. Data Modified : 4/2/2022 3:57:05 PM
Multiplier : 1.0000
Dilution : 1.0000
Use Multiplier & Dilution Factor with ISTDs

Signal 1: FID1 A,
Signal 2: TCD2 B,

RetTime [min]	Sig	Type	Area	Amt/Area	Amount [vol%]	Grp	Name
2.900	2	MM	7183.35059	4.50209e-3	32.34010		H2
8.050	2		-	-	-		Ar
12.106	2		-	-	-		CO
21.000	2		-	-	-		CH4
28.611	2	MM	1.02740e4	1.48829e-4	1.52907		CO2

Figure S46. GC chromatogram of hydrogen production (**Fe-1**, $KHCO_2$, THA).

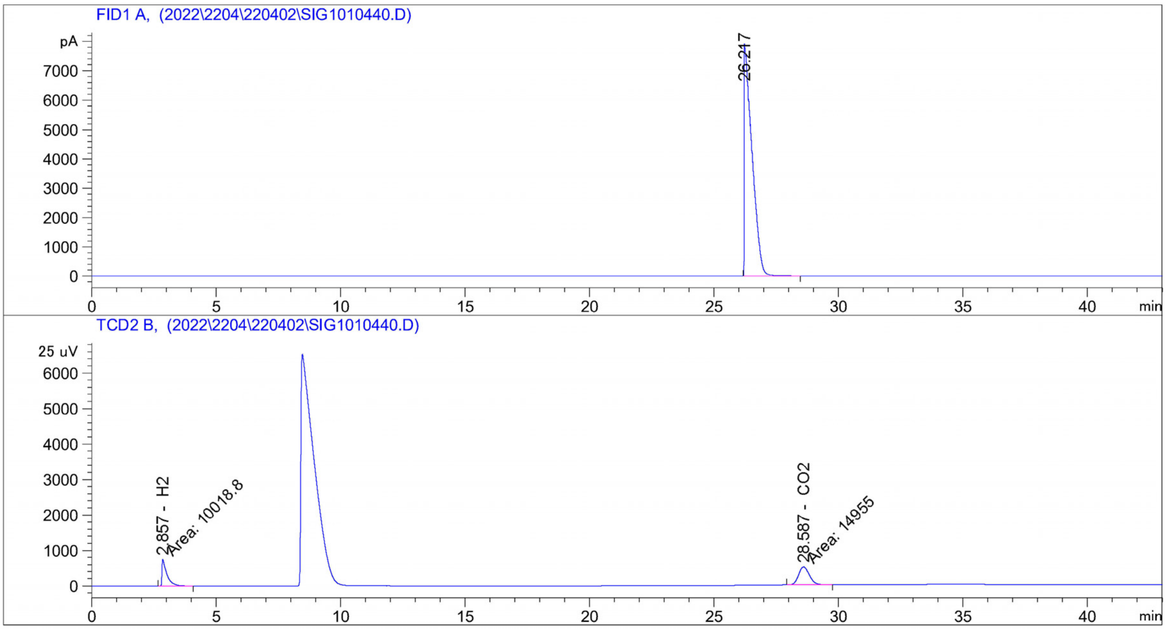


```
=====================================================================
                       External Standard Report
=====================================================================

Sorted By              :      Retention Time
Calib. Data Modified   :      4/2/2022 3:54:24 PM
Multiplier             :      1.0000
Dilution               :      1.0000
Use Multiplier & Dilution Factor with ISTDs

Signal 1: FID1 A,
Signal 2: TCD2 B,

RetTime Sig Type     Area      Amt/Area     Amount    Grp   Name
 [min]                                      [vol%]
-------|--|------|----------|----------|----------|--|---------------
  2.857  2 MM     1.00188e4  4.44442e-3   44.52755     H2
  8.050  2            -          -           -         Ar
 12.106  2            -          -           -         CO
 21.000  2            -          -           -         CH4
 28.587  2 MM     1.49550e4  1.49106e-4    2.22989     CO2
```

Figure S47. GC chromatogram of hydrogen production (**Fe-1**, $KHCO_2$, DMOA).

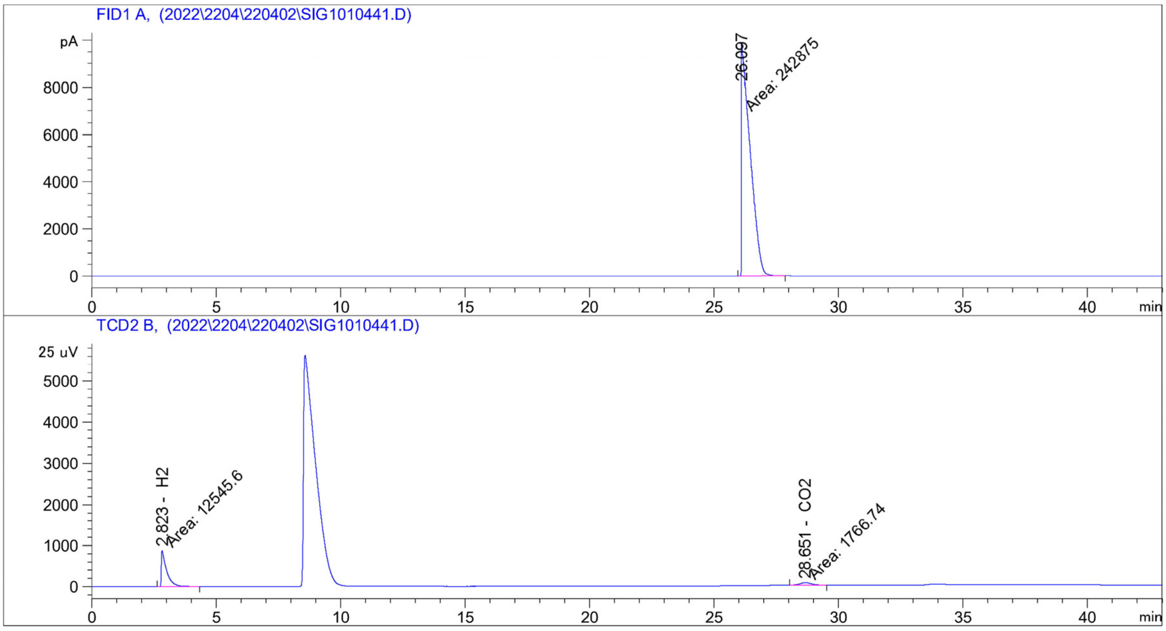


```
=====================================================================
                      External Standard Report
=====================================================================

Sorted By             :      Retention Time
Calib. Data Modified  :      4/2/2022 3:56:10 PM
Multiplier            :      1.0000
Dilution              :      1.0000
Use Multiplier & Dilution Factor with ISTDs

Signal 1: FID1 A,
Signal 2: TCD2 B,
```

RetTime [min]	Sig	Type	Area	Amt/Area	Amount [vol%]	Grp	Name
2.823	2	MM	1.25456e4	4.39563e-3	55.14594		H2
8.050	2		-	-	-		Ar
12.106	2		-	-	-		CO
21.000	2		-	-	-		CH4
28.651	2	MM	1766.73901	1.45622e-4	2.57276e-1		CO2

Figure S48. GC chromatogram of hydrogen production (**Fe-1**, $KHCO_2$, Lys).

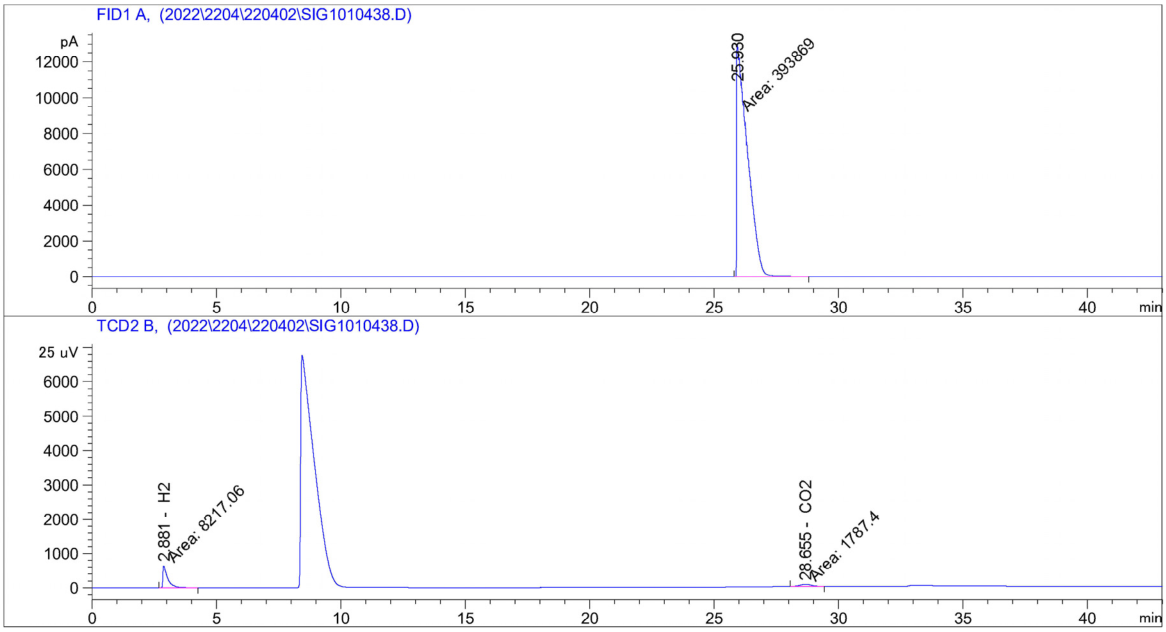


```
=====================================================================
                      External Standard Report
=====================================================================

Sorted By             :      Retention Time
Calib. Data Modified  :      4/2/2022 12:52:11 PM
Multiplier            :      1.0000
Dilution              :      1.0000
Use Multiplier & Dilution Factor with ISTDs

Signal 1: FID1 A,
Signal 2: TCD2 B,
```

RetTime [min]	Sig	Type	Area	Amt/Area	Amount [vol%]	Grp	Name
2.881	2	MM	8217.06250	4.48067e-3	36.81791		H2
8.050	2		-	-	-		Ar
12.106	2		-	-	-		CO
21.000	2		-	-	-		CH4
28.655	2	MM	1787.39709	1.45665e-4	2.60361e-1		CO2

Figure S49. GC chromatogram of hydrogen production (**Fe-1**, $KHCO_2$, Arg).

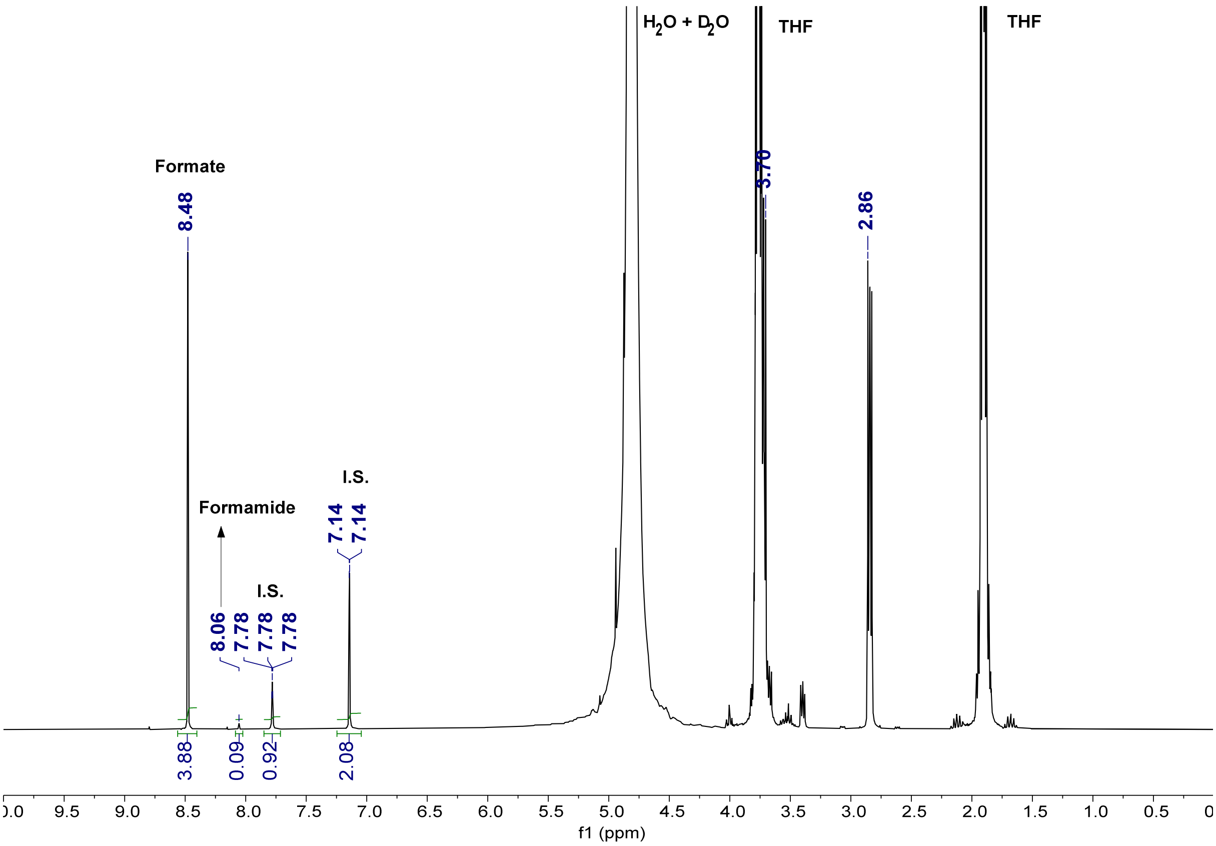

Figure S50. Typical ^{1}H NMR (D_2O) of hydrogenation of $KHCO_3$ to formate in the presence of morpholine (A_1) catalyzed by **Fe-1**. Formate (97%) and formamide (2%) were obtained as products. Imidazole (2.5 mmol) as internal standard (I.S.).

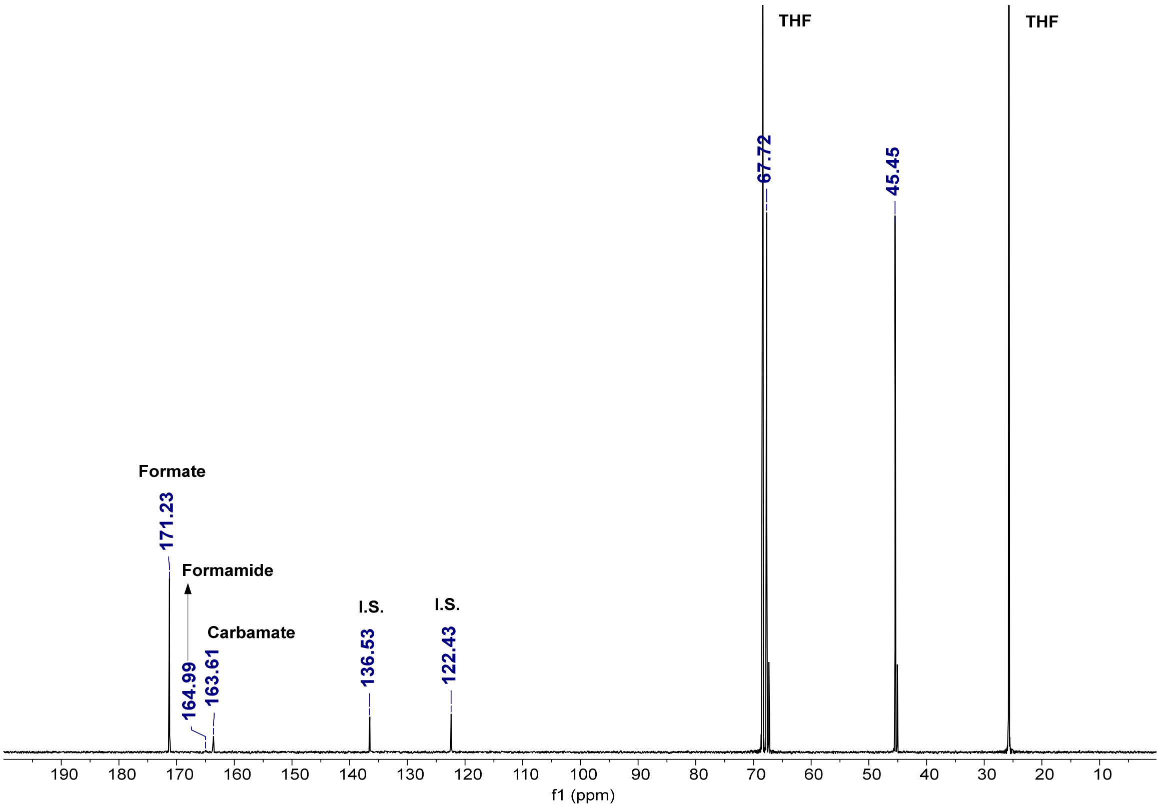

Figure S51. Typical ^{13}C NMR (D_2O) of hydrogenation of $KHCO_3$ to formate in the presence of morpholine (A_1) catalyzed by **Fe-1**. Imidazole (2.5 mmol) as internal standard (I.S.).

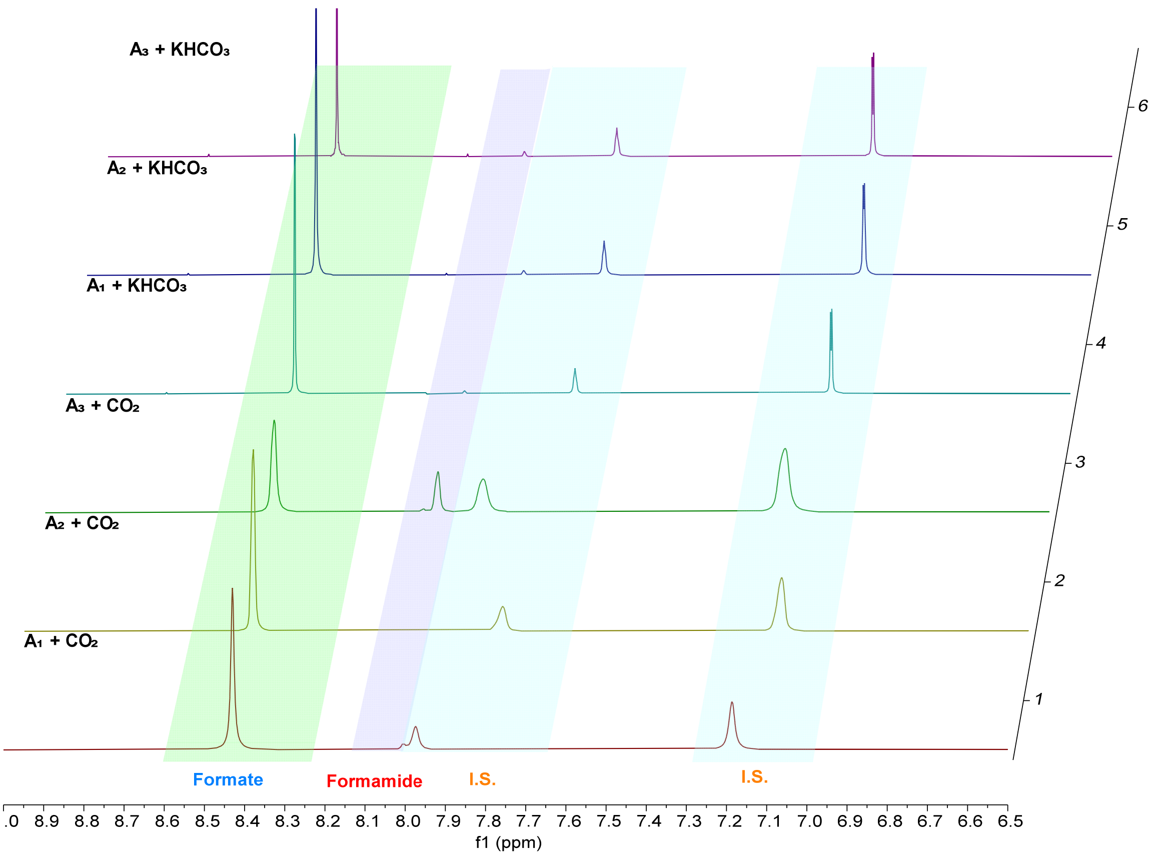

Figure S52. ^{1}H NMR (D_2O) of hydrogenation of CO_2 or potassium bicarbonate in the presence of amines (A_1, A_2, A_3, 10 mmol). Imidazole (2.5 mmol) as internal standard (I.S.). Chemical shifts were calibrated based on formate (8.43 ppm) in D_2O. The chemical shift of internal standard imidazole is slightly shifted due to different presented pH of the reaction solutions.

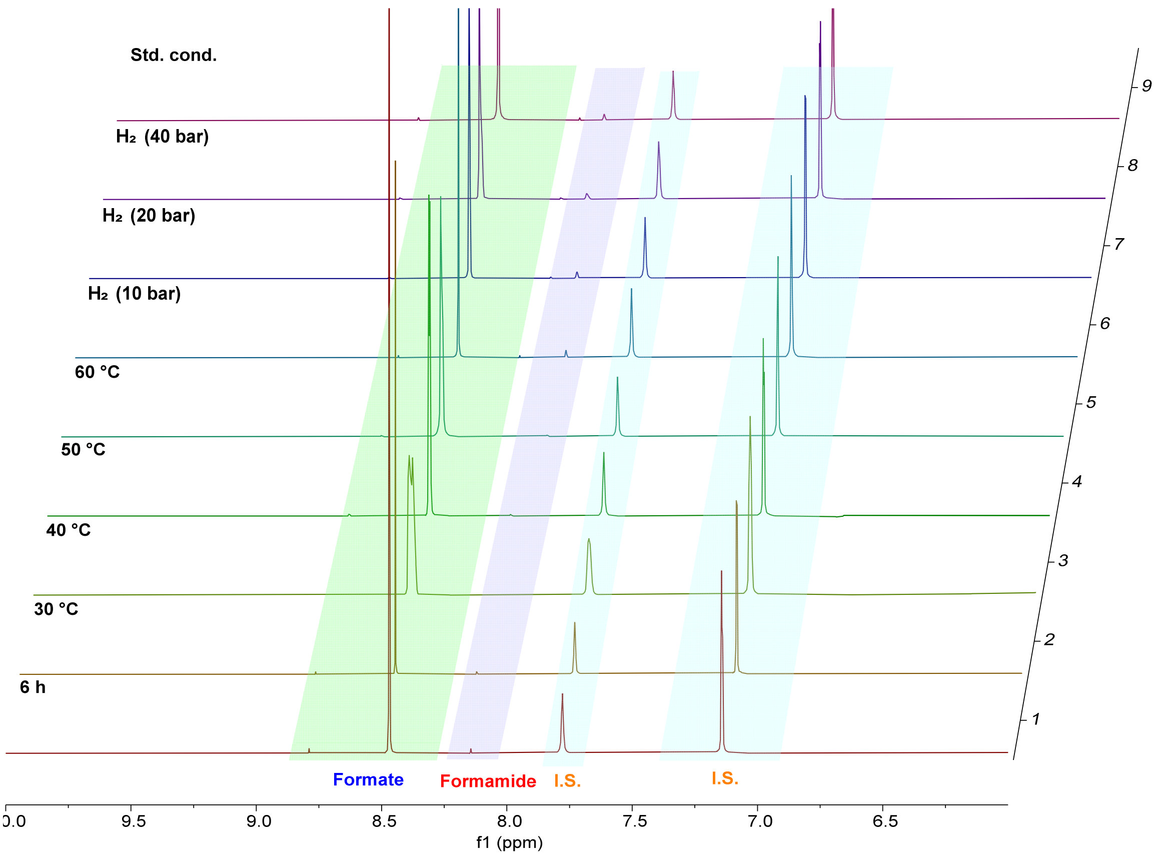

Figure S53. ^{1}H NMR (D_2O) of variation of reaction parameters in hydrogenation of $KHCO_3$ with morpholine (A_1). Imidazole (2.5 mmol) as internal standard (I.S.). Standard conditions: morpholine (A_1, 10 mmol), $KHCO_3$ (10 mmol), **Fe-1** (5 μmol, 500 ppm), H_2 (60 bar), THF/H_2O (5/5 mL), 90 °C, 12 h.

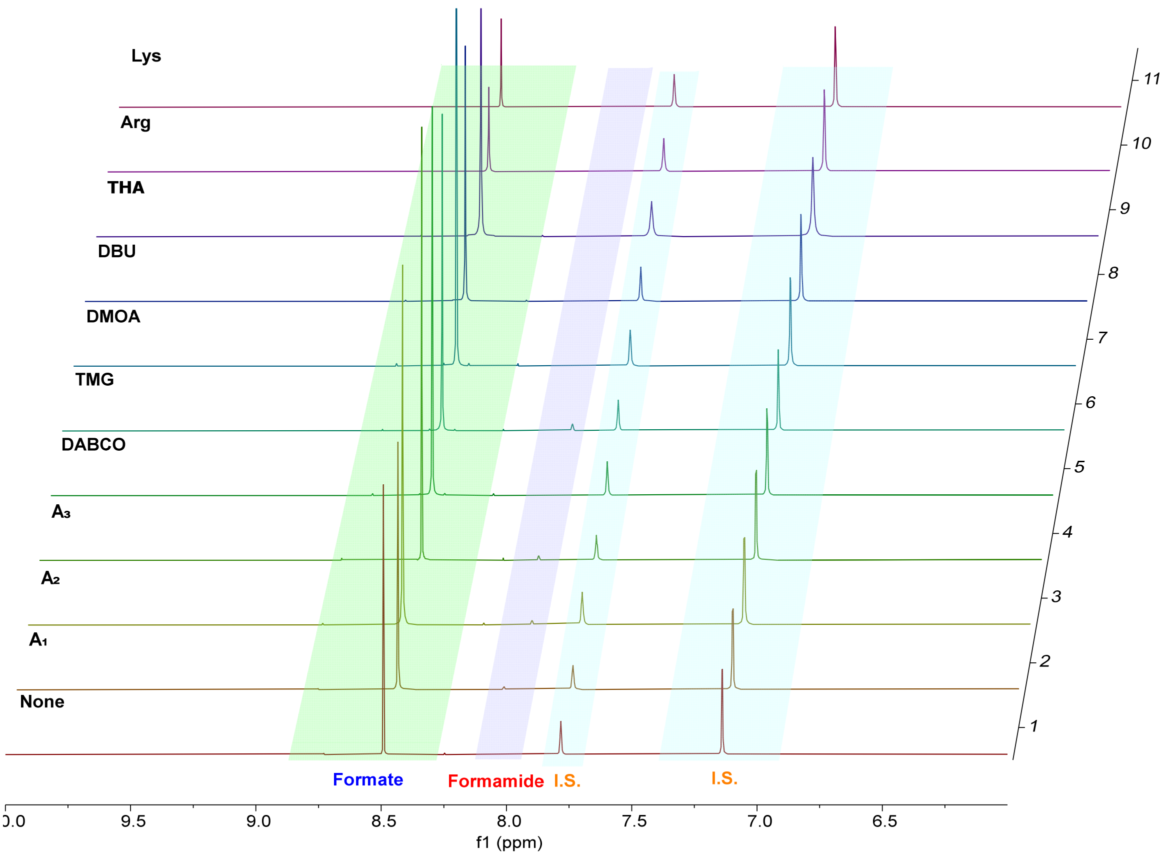

Figure S54. ^{1}H NMR (D_2O) of hydrogenation of potassium bicarbonate (10 mmol) in the presence of various amines (10 mmol). Imidazole (2.5 mmol) as internal standard (I.S.).

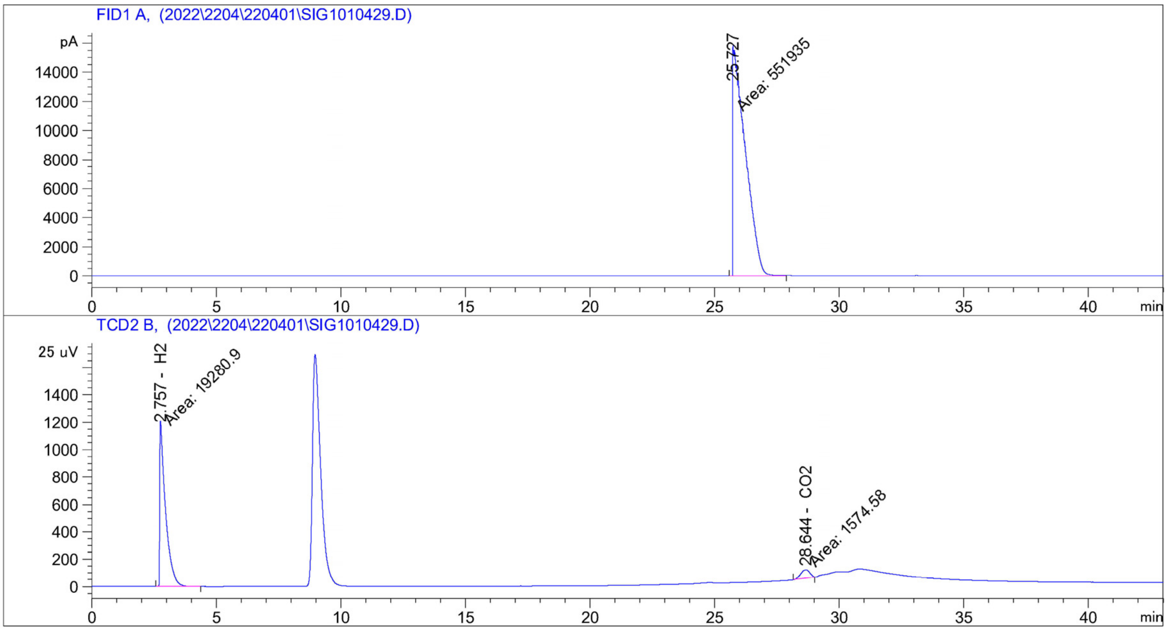

===

External Standard Report

===

Sorted By : Retention Time
Calib. Data Modified : 4/1/2022 12:59:56 PM
Multiplier : 1.0000
Dilution : 1.0000
Use Multiplier & Dilution Factor with ISTDs

Signal 1: FID1 A,
Signal 2: TCD2 B,

RetTime [min]	Sig	Type	Area	Amt/Area	Amount [vol%]	Grp	Name
2.757	2	MM	1.92809e4	4.27549e-3	82.43507		H2
8.050	2		-	-	-		Ar
12.113	2		-	-	-		CO
21.000	2		-	-	-		CH4
28.644	2	MM	1574.58203	1.45167e-4	2.28577e-1		CO2

Figure S55. GC chromatogram of hydrogen storage-release cycles applying *N*-formylmorpholine (F_1, 10 mmol).

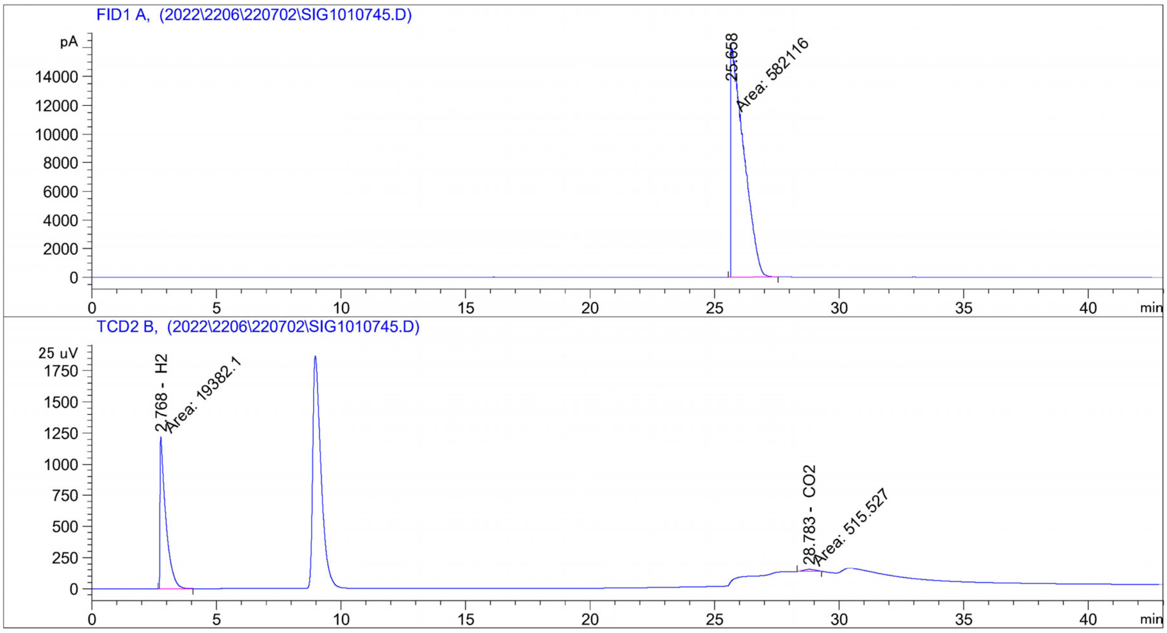


```
=====================================================================
                      External Standard Report
=====================================================================

Sorted By             :      Retention Time
Calib. Data Modified  :      7/13/2022 4:47:50 PM
Multiplier            :      1.0000
Dilution              :      1.0000
Use Multiplier & Dilution Factor with ISTDs

Signal 1: FID1 A,
Signal 2: TCD2 B,
```

RetTime [min]	Sig	Type	Area	Amt/Area	Amount [vol%]	Grp	Name
2.768	2	MM	1.93821e4	4.27378e-3	82.83494		H2
8.828	2		-	-	-		Ar
12.155	2		-	-	-		CO
21.000	2		-	-	-		CH4
28.783	2	MM	515.52661	1.36614e-4	7.04283e-2		CO2

Figure S56. GC chromatogram of hydrogen storage-release cycles applying 1,4-diformylpiperazine (F_4, 5 mmol).

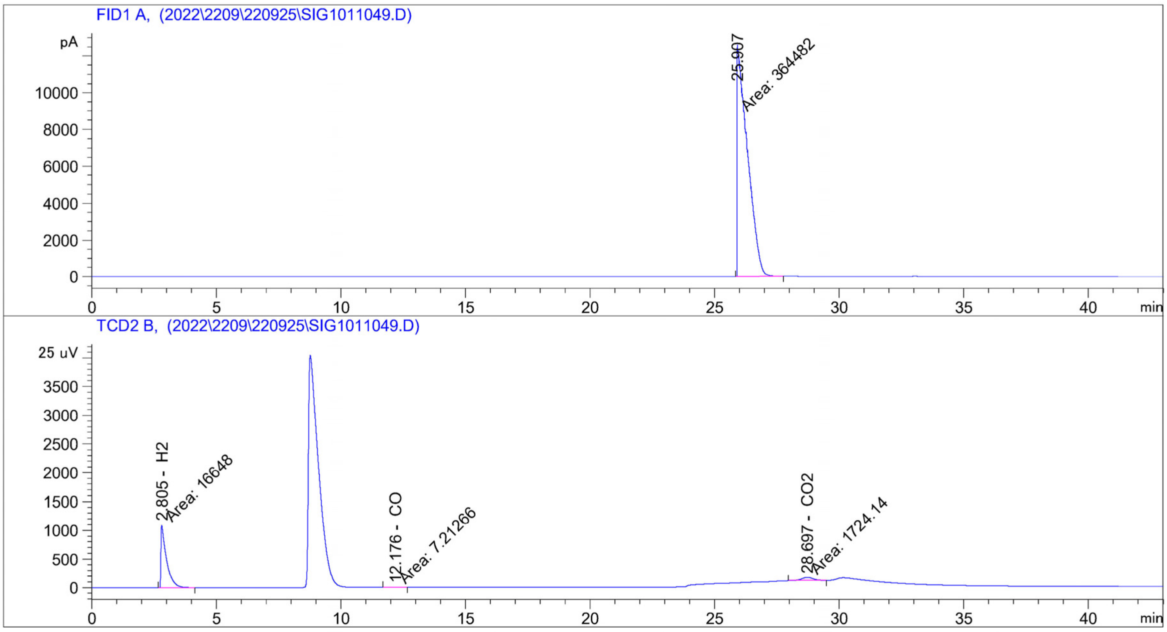


```
=====================================================================
                      External Standard Report
=====================================================================

Sorted By             :      Retention Time
Calib. Data Modified  :      9/29/2022 1:11:13 PM
Multiplier            :      1.0000
Dilution              :      1.0000
Use Multiplier & Dilution Factor with ISTDs

Signal 1: FID1 A,
Signal 2: TCD2 B,
```

RetTime [min]	Sig	Type	Area	Amt/Area	Amount [vol%]	Grp	Name
2.805	2	MM	1.66480e4	4.32089e-3	71.93403		H2
8.050	2		-	-	-		Ar
12.176	2	MM	7.21266	0.00000	0.00000		CO
21.000	2		-	-	-		CH4
28.697	2	MM	1724.14136	1.45530e-4	2.50914e-1		CO2

Figure S57. GC chromatogram of hydrogen storage-release cycles applying *N*-formylmorpholine (F_1, 50 mmol).

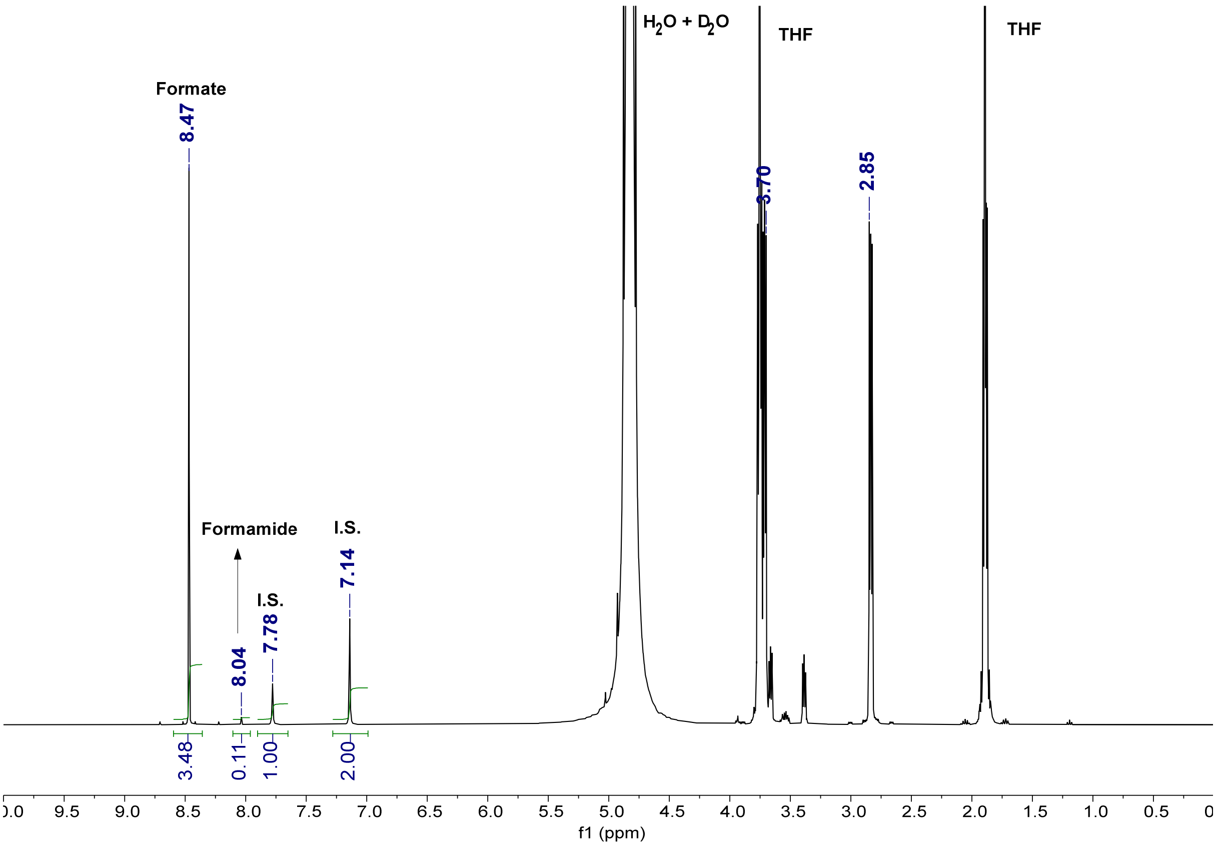

Figure S58. ^{1}H NMR (D_2O) after 10 H_2 storage-release cycles (hydrogenation step) applying *N*-formylmorpholine (F_1, 10 mmol). Imidazole (2.5 mmol) as internal standard.

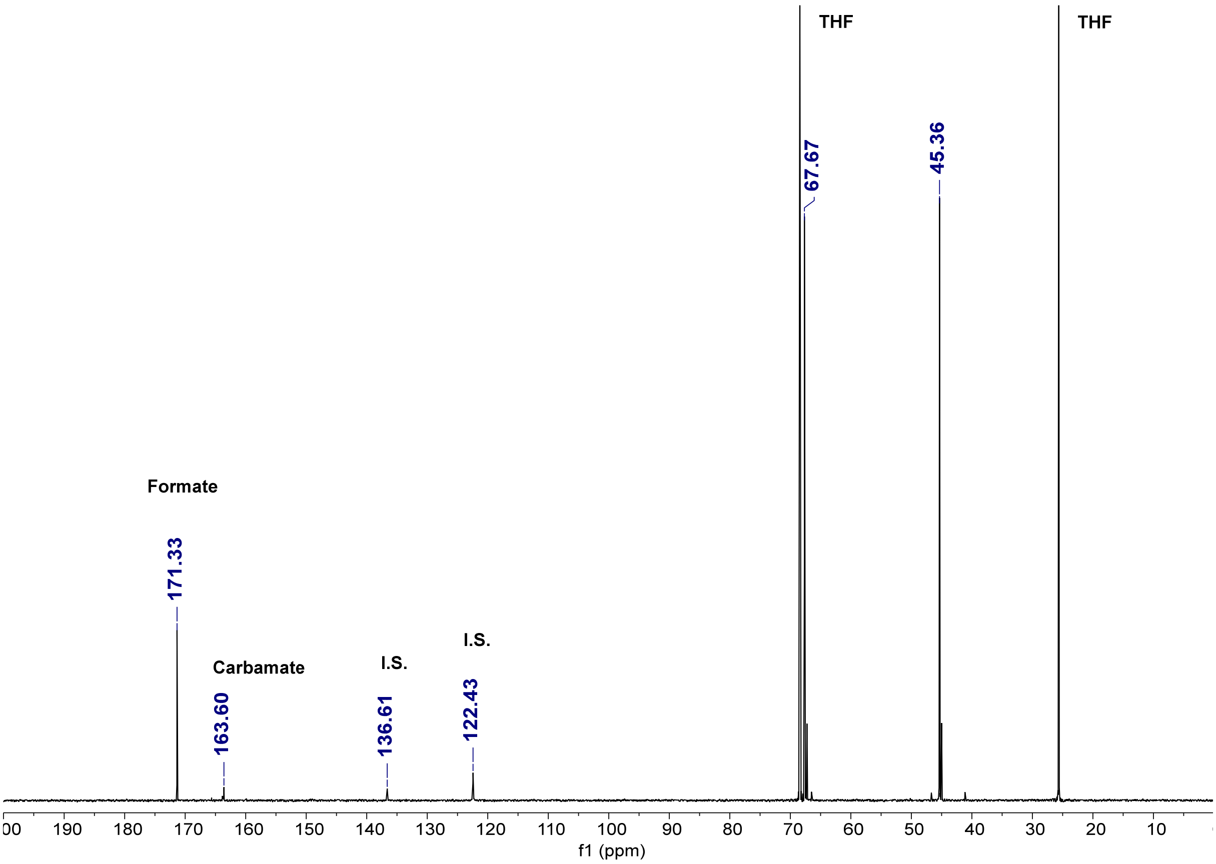

Figure S59. ^{13}C NMR (D_2O) after 10 H_2 storage-release cycles (hydrogenation step) applying *N*-formylmorpholine (F_1, 10 mmol). Imidazole (2.5 mmol) as internal standard.

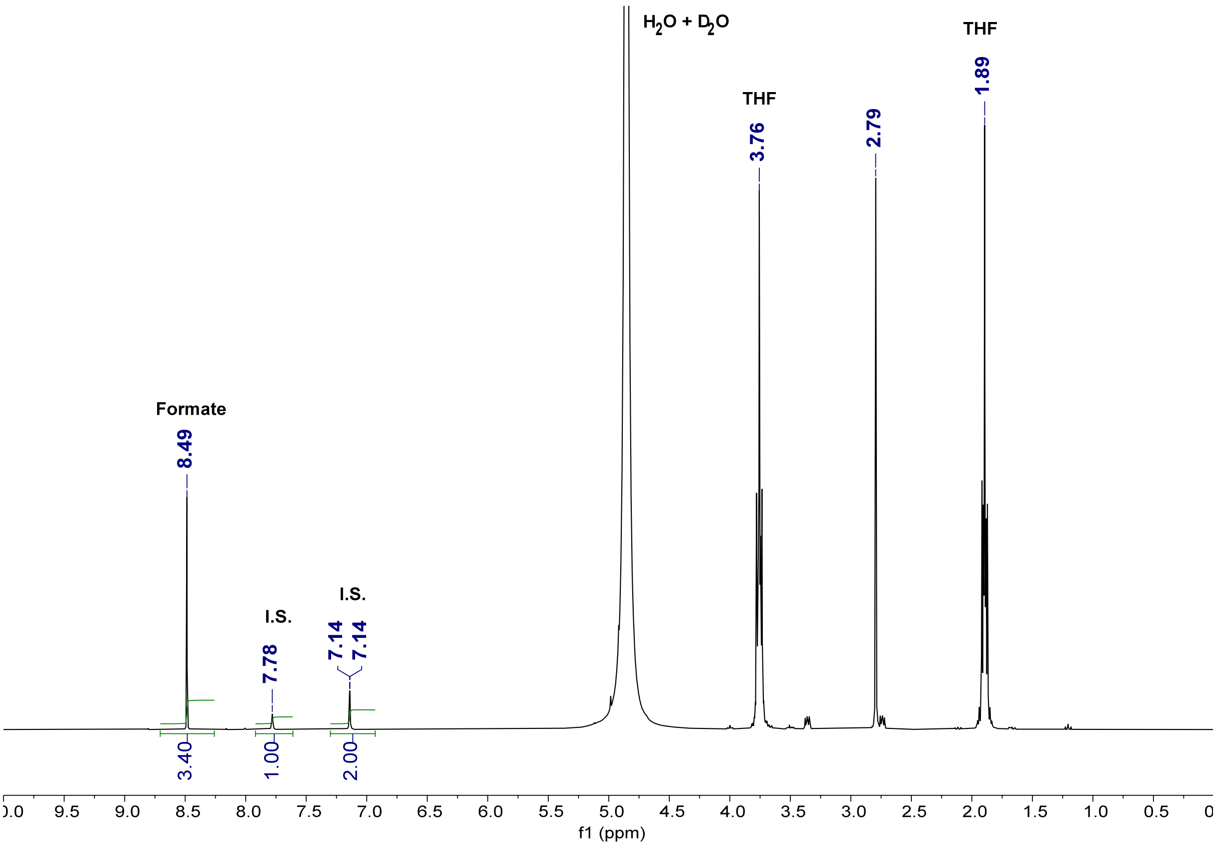

Figure S60. ^{1}H NMR (D_2O) after 10 H_2 storage-release cycles (hydrogenation step) applying 1,4-diformylpiperazine (F_4, 5 mmol). Imidazole (2.5 mmol) as internal standard.

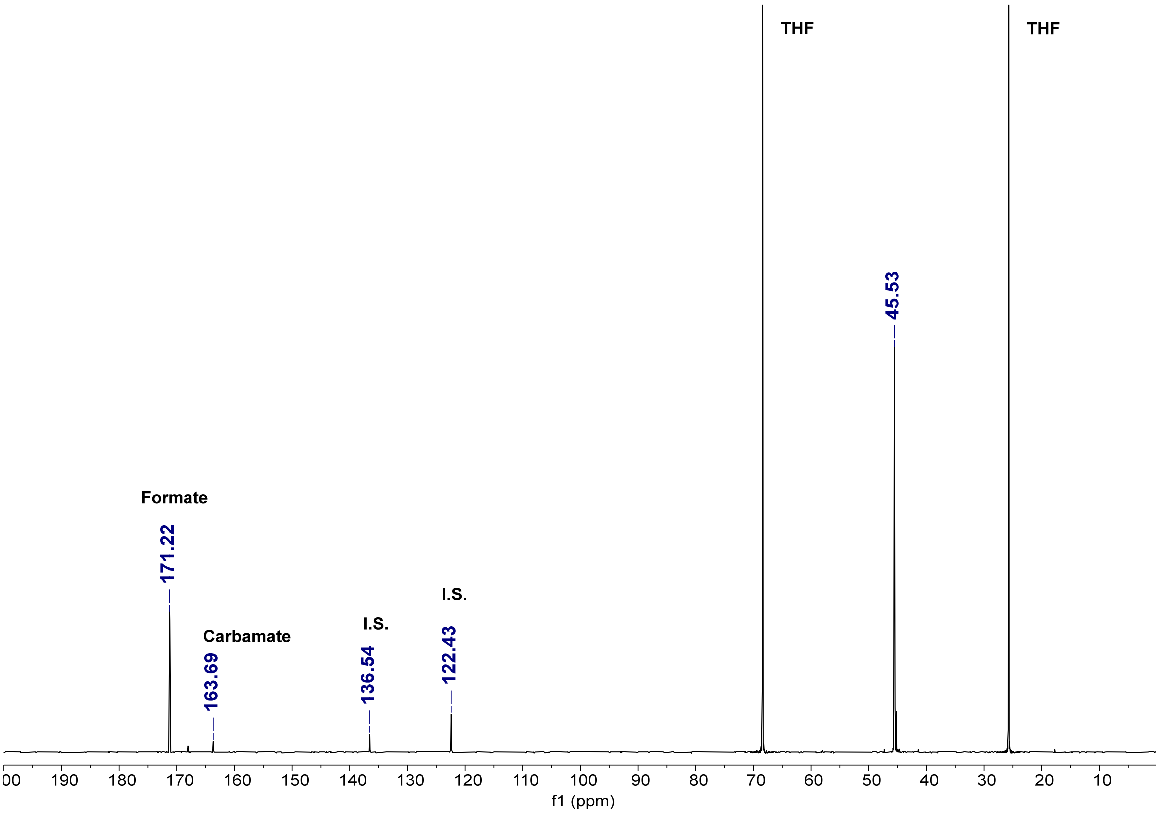

Figure S61. ^{13}C NMR (D_2O) after 10 H_2 storage-release cycles (hydrogenation step) applying 1,4-diformylpiperazine (F_4, 5 mmol). Imidazole (2.5 mmol) as internal standard.

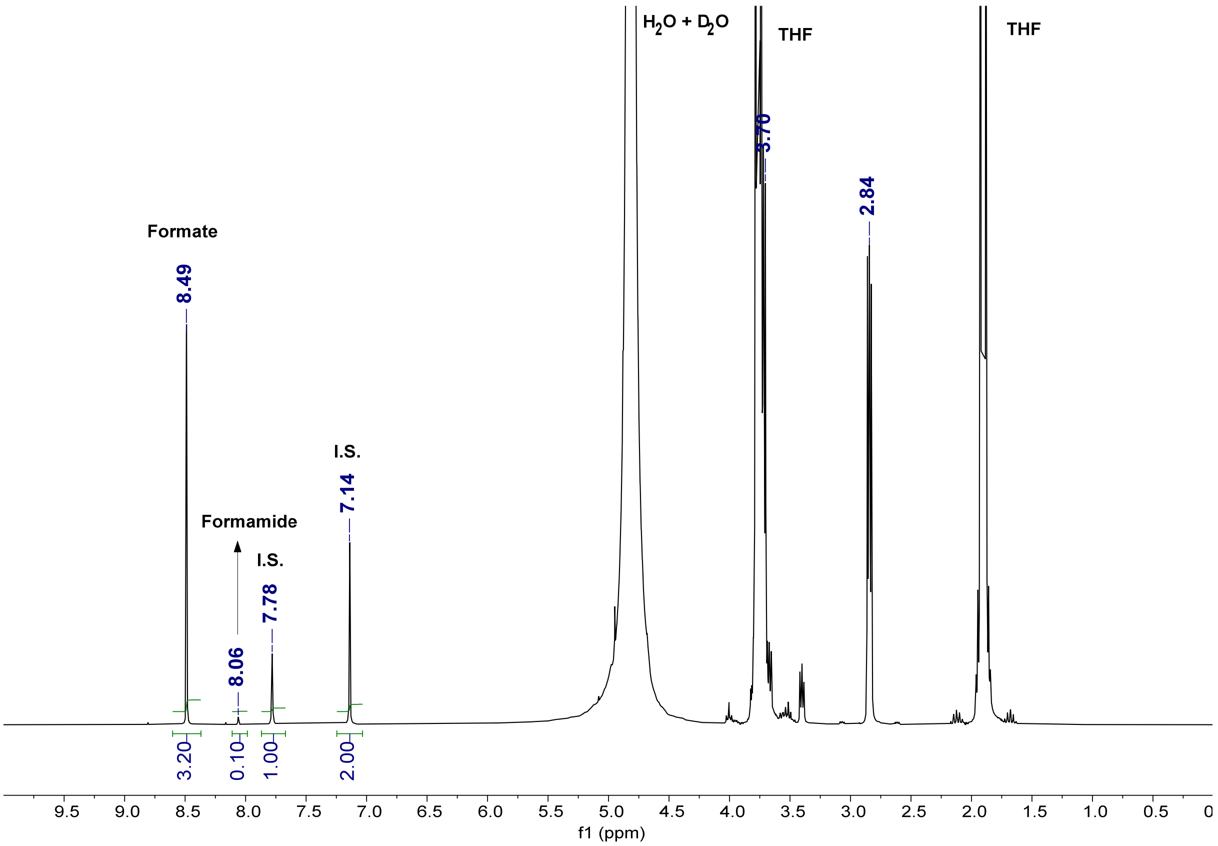

Figure S62. ^{1}H NMR (D_2O) after 10 H_2 storage-release cycles (hydrogenation step) applying *N*-formylmorpholine (F_1, 50 mmol). Imidazole (12.5 mmol) as internal standard.

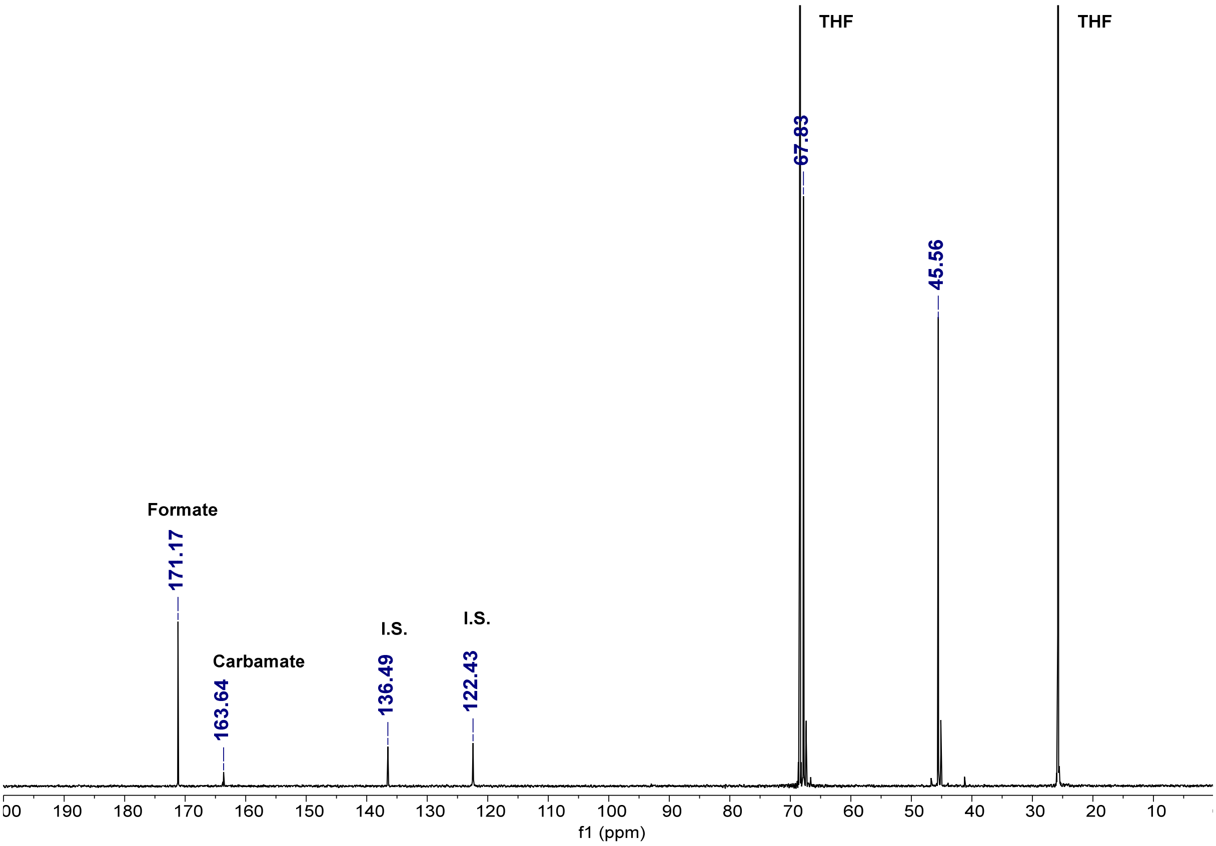

Figure S63. ^{13}C NMR (D_2O) after 10 H_2 storage-release cycles (hydrogenation step) applying *N*-formylmorpholine (F_1, 50 mmol). Imidazole (12.5 mmol) as internal standard.

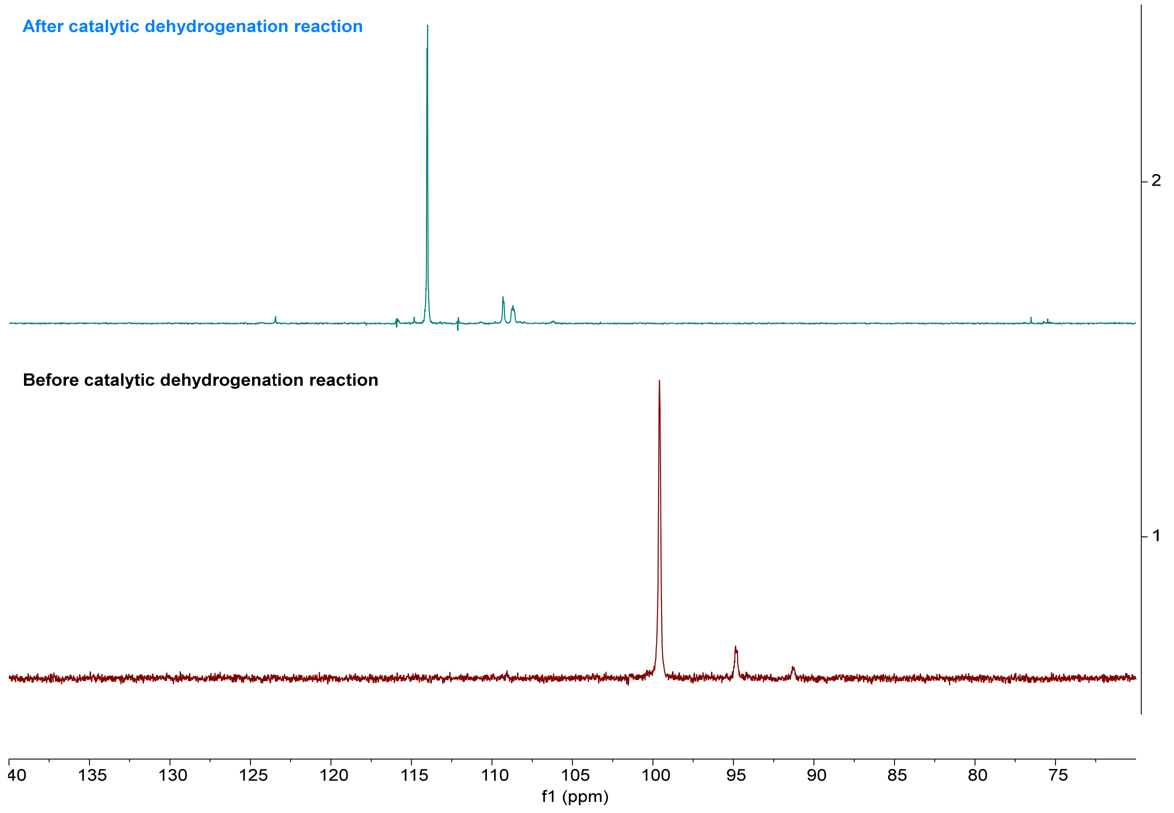

Figure S64. $^{31}P\{^1H\}$ NMR (THF-d^8/D_2O) before and after catalytic dehydrogenation reactions. Conditions: **Fe-1** (10.0 mg, 24.7 μmol), *N*-formylmorpholine (F_1, 0.1 mmol), KOH (0.1 mmol), 90 °C, 16 h.

Table S1. Time dependent production of hydrogen storage and release reactions catalysed by **Fe-1**.

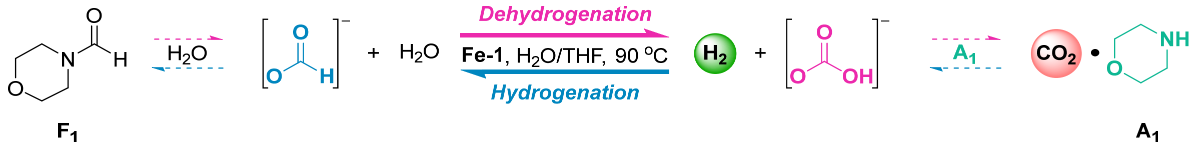

Entry	Reaction	Time (h)	Formate yield (%)	Formamide yield (%)	H_2 yield (%)	H_2 selectivity (%)
1	Hydrogenation	3	65	1	*n/a*	*n/a*
2		6	86	1	*n/a*	*n/a*
3		12	94	6	*n/a*	*n/a*
4	Dehydrogenation	4	*n/a*	*n/a*	29	99.97
5		8	*n/a*	*n/a*	49	100
6		16	*n/a*	*n/a*	99	99.98

General conditions: Hydrogenation: morpholine (A_1, 10 mmol), **Fe-1** (5 μmol), THF/H_2O (5/5 mL), CO_2/H_2 (20/60 bar), 90 °C. Dehydrogenation: *N*-formylmorpholine (F_1, 10 mmol), KOH (10 mmol), **Fe-1** (5 μmol), THF/H_2O (5/5 mL), 90 °C. n/a: not applicable.

Table S2. H_2 evolution in the H_2 storage-release cycles applying formamides.

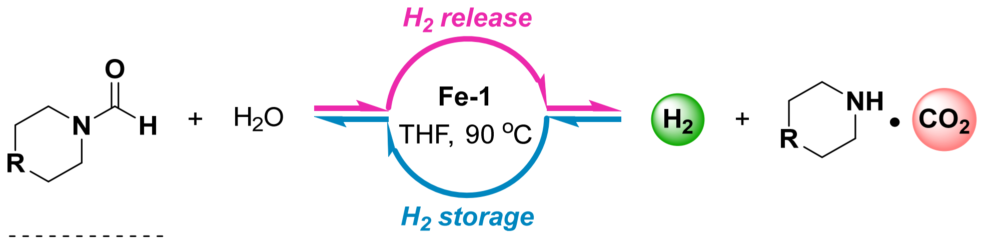

R = O, *N*-formylmorpholine (**F$_1$**)
R = N-CHO, 1,4-diformylpiperazine (**F$_4$**)

Substrate / Cycle	F$_1$ (10 mmol)		F$_4$ (5 mmol)		F$_1$ (50 mmol)	
	Yield %	Selectivity %	Yield %	Selectivity %	Yield %	Selectivity %
1	99	99.37	100	99.42	86	99.96
2	85	99.51	82	99.88	82	99.95
3	84	99.51	75	99.45	78	99.65
4	89	99.06	77	99.65	76	99.51
5	82	99.65	80	99.70	72	99.36
6	83	99.02	81	99.54	70	99.94
7	84	99.57	77	99.59	73	99.93
8	82	99.72	70	99.91	75	99.42
9	85	99.53	77	99.74	72	99.91
10	83	99.69	75	99.45	70	99.90

[a] Yield of H_2 is calculated by (mmol H_2)/(mmol formyl group in formamides)×100%.

Supplementary references

1. Chakraborty, S. *et al.* Iron-Based Catalysts for the Hydrogenation of Esters to Alcohols. *J. Am. Chem. Soc.* **136**, 7869–7872, (2014).
2. Bielinski, E. A. *et al.* Lewis Acid-Assisted Formic Acid Dehydrogenation Using a Pincer-Supported Iron Catalyst. *J. Am. Chem. Soc.* **136**, 10234–10237, (2014).
3. Alberico, E. *et al.* Selective Hydrogen Production from Methanol with a Defined Iron Pincer Catalyst under Mild Conditions. *Angew. Chem. Int. Ed.* **52**, 14162–14166, (2013).
4. Werkmeister, S. *et al.* Hydrogenation of Esters to Alcohols with a Well-Defined Iron Complex. *Angew. Chem. Int. Ed.* **53**, 8722–8726, (2014).
5. Elangovan, S. *et al.* Selective catalytic hydrogenations of nitriles, ketones, and aldehydes by well-defined manganese pincer complexes. *J. Am. Chem. Soc.* **138**, 8809–8814, (2016).
6. Elangovan, S. *et al.* Efficient and selective *N*-alkylation of amines with alcohols catalysed by manganese pincer complexes. *Nat. Commun.* **7**, 12641, (2016).
7. Kallmeier, F., Irrgang, T., Dietel, T. & Kempe, R. Highly Active and Selective Manganese C=O Bond Hydrogenation Catalysts: The Importance of the Multidentate Ligand, the Ancillary Ligands, and the Oxidation State. *Angew. Chem. Int. Ed.* **55**, 11806–11809, (2016).
8. Zhou, W. *et al.* Cobalt-Catalyzed Aqueous Dehydrogenation of Formic Acid. *Chem. Eur. J.* **25**, 8459–8464, (2019).
9. Alberico, E. *et al.* HCOOH disproportionation to MeOH promoted by molybdenum PNP complexes. *Chem. Sci.* **12**, 13101–13119, (2021).
10. Kar, S., Sen, R., Goeppert, A. & Prakash, G. K. S. Integrative CO_2 Capture and Hydrogenation to Methanol with Reusable Catalyst and Amine: Toward a Carbon Neutral Methanol Economy. *J. Am. Chem. Soc.* **140**, 1580–1583, (2018).
11. Wei, D., Junge, H. & Beller, M. An amino acid based system for CO_2 capture and catalytic utilization to produce formates. *Chem. Sci.* **12**, 6020–6024, (2021).
